# Origin and Evolution of Allopolyploid Wheatgrass *Elymus fibrosus* (Schrenk) Tzvelev (Poaceae: Triticeae) Reveals the Effect of Its Origination on Genetic Diversity

**DOI:** 10.1371/journal.pone.0167795

**Published:** 2016-12-09

**Authors:** De-Chuan Wu, Deng-Min He, Hai-Lan Gu, Pan-Pan Wu, Xu Yi, Wei-Jie Wang, Han-Feng Shi, De-Xiang Wu, Genlou Sun

**Affiliations:** 1 College of Agronomy, Anhui Agricultural University, Hefei, Anhui, China; 2 Biology Department, Saint Mary’s University, Halifax, NS, Canada; Murdoch University, AUSTRALIA

## Abstract

Origin and evolution of tetraploid *Elymus fibrosus* (Schrenk) Tzvelev were characterized using low-copy nuclear gene *Rpb2* (the second largest subunit of RNA polymerase II), and chloroplast region *trnL*–*trnF* (spacer between the tRNA Leu (UAA) gene and the tRNA-Phe (GAA) gene). Ten accessions of *E*. *fibrosus* along with 19 *Elymus* species with **StH** genomic constitution and diploid species in the tribe Triticeae were analyzed. Chloroplast *trnL*–*trnF* sequence data suggested that *Pseudoroegneria* (**St** genome) was the maternal donor of *E*. *fibrosus*. *Rpb2* data confirmed the presence of **StH** genomes in *E*. *fibrosus*, and suggested that **St** and **H** genomes in *E*. *fibrosus* each is more likely originated from single gene pool. Single origin of *E*. *fibrosus* might be one of the reasons causing genetic diversity in *E*. *fibrosus* lower than those in *E*. *caninus* and *E*. *trachycaulus*, which have similar ecological preferences and breeding systems with *E*. *fibrosus*, and each was originated from multiple sources. Convergent evolution of **St** and **H** copy *Rpb2* sequences in some accessions of *E*. *fibrosus* might have occurred during the evolutionary history of this allotetraploid.

## Introduction

The tribe Triticeae includes not only the world’s most economically important cereal crops and forage grasses, but also troublesome weeds distributed all over the world. Within this tribe, three quarters of the species are polyploids [1].

*Elymus* L. as delimited by Löve [2] is the largest genus with exclusively allopolyploids including approximately 150 species. Due to the abundance of polyploid species in *Elymus* and closely related diploid species from various taxa in Triticeae available, *Elymus* is an ideal model for examining the impact of polyploidization on speciation, and the role of alloployploidy as a driver of plant diversification [3]. Cytogenetically, five basic genomes (**St**, **H**, **Y**, **P**, and **W**) (genome symbols follow Wang et al., [4]) have been assigned to the species in this genus [5, 6]. The majorities of species are tetraploids and characterized by having either **StH** or the **StY** genomic combination. For **StH** genome species, the **St** genome was suggested maternally donated by *Pseudoroegneria* (Nevski) Á. Löve [7–13]. The **H** genome was derived from *Hordeum* L. The **StH** genome *Elymus* species have adapted to an enormously wide range of climates and habitats, making them of special interest for speciation and ecological research [3].

*Elymus fibrosus* (Schrenk) Tzvel. is one of **StH** genome species with a predominantly self-pollinating, distributed in Russia and northern Scandinavia [14]. It grows on wet meadows, riverside sand and pebbles, and among shrubs. It is usually found growing alone or sympatrically with *E*. *caninus*, *E*. *sibiricus* L., and *E*. *repens* (L.) Gould. It has been reported low genetic variation and deficiency in heterozygosity in *E*. *fibrosus* population, which might be caused by a potential bottleneck [15, 16].

Phylogenetic analyses have demonstrated multiple origins of the **H** and **St** haplome in the **StH** tetraploid species of *Elymus* [17–22] and reticulate evolution in the *Elymus* [17, 18, 20–22]**.** The studies on genetic diversity of *E*. *caninus* [23, 24], *E*. *fibrosus* [15, 16], *E*. *alaskanus* [25–28], and *E*. *trachycaulus* complex [29, 30] have shown that despite some of these four species having similar ecological preferences and breeding systems, their population structure and genetic variation deviated highly, and each species possesses a unique pattern of genetic variation. Multiple origins are often considered as a potential source for increasing genetic variation in polyploids [31]. Our previous studies have shown multiple origins of allopolyploid wheatgrass *E*. *caninus* [21] and *E*. *trachycaulus* [22]. *Elymus fibrosus* is a tetraploid containing genomes of a *Pseudoroegneria* species (**St** genome) and a wild *Hordeum* species (**H** genome) [1], but its origin was rarely explored at molecular level.

In this study, single copy nuclear gene, the second largest subunit of RNA polymerase II (*Rpb2*), and chloroplast DNA *TrnL–trnF* region (spacer between the tRNA-Leu (UAA) gene and the tRNA-Phe (GAA) gene) were used to explore the origin of tetraploid *E*. *fibrosus*. The effect of origination on genetic diversity of this species was discussed.

## Materials and Methods

### Plant materials and DNA extraction

DNA from ten accessions of *E*. *fibrosus* species was extracted from fresh young leaf tissues using the method of Junghans and Metzlaff [32], and amplified using low copy nuclear gene *Rpb2* and chloroplast *TrnL-F* sequences. *Rpb2* and *TrnL-F* sequences from 19 *Elymus* **StH** genome species and some diploid Triticeae species representing the **St**, **H**, **I**, **Xa**, **Xu**, **W**, **P**, **E** and **V** genomes included in the analyses were downloaded from GenBank (NCBI) or obtained from the published data [17, 21, 33]. Accession number, genomic constitution, geographical origin, and GenBank identification number of these materials are listed in Table 1. The voucher specimens of *E*. *fibrosus* were deposited at Anhui Agricultural University.

**Table 1:** Taxa from *Bromus*, *Aegilops*, *Eremopyrum*, *Heteranthelium*, *Psathyrostachys*, *Secale*, *Taeniatherum*, *Agropyron*, *Australopyrum*, *Dasypyrum*, *Thinopyrum*, *Pseudoroegneria*, *Hordeum* and *Elymus* used in this study, their origin, accession number and GenBank sequence number.

Species	Accession No.	Genome	Origin	*RPB2*	*TrnL/F*
*B*. *sterilis* L.	PI 229595		Iran	HQ231839	
*B*. *tectorum* L.			NA	-	AB732928
*Aegilops sharonensis* Eig.	PI584396	S^1^	Israel		EU013659
*Aegilops speltoides* Tausch	Morrison s.n.	S	NA	-	AF519112
*Eremopyrum bonaepartis* (Spreng.) Nevski	H5554	F	NA	-	AF519148
*Eremopyrum orientale* (L.) Jaub. & Spach	H 5555	F	NA	-	AF519151
*Heteranthelium piliferum* (Banks & Sol.) Hochst.	PI 402352	Q	Iran	-	AF519153
*Henrardia persica* (Boiss.) C.E.Hubb.					AF519152
*Psathyrostachys juncea* (Fischer) Nevski	PI206684	Ns	Turkey	-	AF519170
*Psathyrostachys fragilis* (Boiss.) Nevski					AF519169
*Secale cereale* L.	Kellogg s.n.	R	NA	-	AF519162
*Taeniatherum caput-medusae* (L.) Nevski	MB-106-41-79	Ta	NA	-	AF519164
*Agropyron cristatum* (L.) Gaertn.	PI 383534	P	Kars, Turkey	EU187438	-
					AF519115
*Ag*. *mongolicum* Keng	D2774	P	NA	-	AF519117
*Aust*. *retrofractum* (Vickery) Á. Löve	Crane 86146	W	NA	-	AF519118
*Aust*. *retrofractum* (Vickery) Á. Löve	PI 533014	W	New South Wales, Australia	EU187482	
	PI 547363	W	New South Wales, Australia	EU187470	
*Dasypyrum villosum* (L.) Candargy	PI251478	V	Turkey	-	AF519128
*Thinopyrum elongatum* (Host) D.R.Dewey	PI 142012	E^e^	Russia Federation	EU187439	-
*Thinopyrum elongatum* (Host) D.R.Dewey	PI531719	E^e^	France	-	AF519166
*Thinopyrum bessarabicum* (Savul. & Rayss) Á.Löve	PI531711	Eb	Estonia	-	AF519165
*Thinopyrum bessarabicum* (Savul. & Rayss) Á. Löve	PI 531712	E^b^	Estonia	EU187474	
*Thinopyrum* intermedium (Host.) Barkworth & D.R. Dewey					DQ912410
*H*. *vulgare* L.	HT025	I	NA	-	AJ969295
*H*. *spontaneum* K. Koch	HT025	I	NA	-	AJ969296
*H*. *bulbosum* L.	PI 440417	I	NA	-	AF519122
*H*. *marinum* Huds	PI 304346	Xa	California, USA	-	AF519124
*H*. *marinum* subsp. *gussoneanum* (Parlatore) Thellung		Xa	NA	-	AB732935
*H*. *murinum* L.	PI 247054	Xu	California, USA	-	AF519125
*H*. *muticum* J. Presl	HT043	H	NA	-	AJ969330
*H*. *pusillum* Nutt.		H	NA	-	AB732937
	H2024	H	U.S.A	+	
*H*. *comosum* J. Presl	HT060	H	NA	-	AJ969362
*H*. *euclaston* Steud.	H2148	H	Uruguay	+	
*H*. *intercedens* Nevski	H1941	H	U.S.A	+	
*H*. *pubiflorum* Hook. f	HT075	H	NA	-	FM163499
*H*. *bogdanii* Wilensky	H4014	H	NA	+	
*H*. *bogdanii* Wilensky	PI531761	H	China	-	AY740789
*H*. *roshevitzii* Bowden	HT005	H	NA	-	AJ969271
	H9152	H	NA	+	-
*H*. *chilense* Roem. and Schult.	HT053	H	NA	-	AJ969351
	H1816	H	Chile	+	
*H*. *patagonicum* (Haumann) Covas	HT046	H	NA	-	AJ969336
*H*. *brachyantherum* subsp. *californicum* (Covas & Stebbins) Bothmer et al.		H	NA	-	KF600706
*H*. *brachyantherum* Nevski		H	NA	-	AJ969314
*P*. *libanotica* (Hack.) D. R. Dewey	PI 228391	St	Iran	-	AF519156
	PI 228390	St	NA	+	
*P*. *spicata* (Pursh) Á. Löve	PI 610986	St	Utah, United States	-	AF519158
	PI 537379	St	NA	+	
	PI 516184	St	NA	+	
	D2839	St	NA	-	AF519160
*P*. *stipifolia* (Czern. ex Nevski) Á. Löve	PI 440095	St	Russian Federation	+	EU617255
*P*. *strigosa* (M. Bieb.) Á. Löve	PI 531752	St	Estonia	HQ231850	EU617284
	PI 499637	St	China	-	EU617269
	PI531753	St	Estonia	-	EU617283
*P*. *gracillima* (Nevski) Á.Löve	PI 440000	St	Stavropol, Russian Federation	-	EU617289
*P*. *tauri* (Boiss. & Balansa) Á. Löve	PI 401326	St	NA	+	
*Elymus alaskanus* (Scribn. & Merr.) Á. Löve	H10476	StH	NA	+	
*Elymus Canadensis* L.					AF519133
	PI 531576	StH	NA	+	
*Elymus caninus* (L.) L.		StH	NA		KF600685
	H3169	StH	NA	EF596700	
*Elymus dentatus* (Hook. f.) Tzvelev					DQ159290
	PI 628702	StH	NA	EF596744,EF596769	
*Elymus elymoides* (Raf.) Swezey					AF519135
*Elymus confusus* (Roshev.) Tzvelev	W6 21505	StH	NA	+	
*Elymus glaucus* Buckley					AF519138
	PI 232258	StH	NA	EF596757	
*Elymus hystrix* L.					AF519139
	H5495	StH	NA	EF596765	
*Elymus lanceolatus* (Scribn. & J. G. Sm.) Gould					AF519140
	PI 236663	StH	NA	EF596739	
*Elymus mutabilis* (Drobow) Tzvelev					DQ159291
*Elymus virescens* Piper	H10584	StH	NA	EF596742,EF596766	
*Elymus virginicus* L.		StH	NA		AF519145
	PI 436946	StH	NA	EF596759	
*Elymus wiegandii* Fernald	PI 531708	StH	NA	EF596740,EF596758	
*Elymus sibiricus* L.					KF905221
	PI 499461	StH	NA	EF596741,EF596763	
*Elymus scabriglumis* (Hack.) Á. Löve	PI 331168	StHH	NA	EF596756	
*Elymus trachycaulus* (Link) Gould ex Shinners	PI372500	StH	Northwest Territory, Canada	-	AF519141
*Elymus trachycaulus* (Link) Gould ex Shinners	H3526	StH	Nerungri, Russia	EF596743,EF596764	
*E*. *transbaicalensis* (Nevski) Tzvelev	H10391	StH	Siberia, Russia	EF596762,EF596745	
*Elymus wawawaiensis* Ined.		StH	NA		AF519147
	PI 506262	StH	NA	EU187441	
*Elymus firbrosus* (Schrenk) Tzvelev	PI439999	StH	Russian Federation	+, +	+
	PI 345585	StH	Former Soviet Union	+	+
	PI 564930	StH	Russian Federation	+,+	+
	PI564933	StH	Kazakhstan	+, +	+
	PI 598465	StH	Russian Federation	+, +	+
	PI 406467	StH	Former Soviet Union	+, +	+
	PI 531609	StH	Germany	+,+	+
	PI 406448	StH	Former Soviet Union	+, +	+
	PI 564932	StH	Russian Federation	+, +	+
	H10339	StH	Pelkosniemi, Finland	EF596773	-

NA: information not available

+: sequence present

-: sequence absent

### DNA amplification and sequencing

The single and low copy nuclear gene *Rpb2* and cpDNA gene *TrnL-F* sequences were amplified by polymerase chain reaction (PCR) using the primers P6F and P6FR [33], and *TrnL* and *TrnF* [9], respectively. The sequences of *Rpb2* and *TrnL-F* region were amplified in a 20 μl reaction containing 20 ng template DNA, 0.25 mM dNTP, 2.0 mM MgCl2, 0.25 μM of each primer and 2.0 U *Taq* polymerase (TransGen, Beijing, China). The amplification profiles for the *Rpb2* gene and *TrnL-F* were described in Zuo et al. [22]. PCR products were purified using the EasyPure Quick Gel Extraction Kit (TransGen, Beijing, China) according to manufacturer’s instruction.

The amplified PCR products of *Rpb*2 gene were cloned into the pGEM-easy T vector (Promega Corporation, Madison, Wis., USA), and transformed into *E*. *coli* competent cell DH5α according to the manufacturer’s instruction (TransGen, Beijing, China). At least 10 clones from each accession were screened and sequenced. Both the PCR products and positive colonies were commercially sequenced by the Shanghai Sangon Biological Engineering & Technology Service Ltd (Shanghai, China). Each PCR product amplified by cpDNA primer *TrnL-F* was independently amplified twice in order to avoid any error which would be induced by *Taq* DNA polymerase during PCR amplification, since *Taq* error that cause substitution is mainly random and it is unlikely that any two sequences would share identical *Taq* errors to create a false synapomorphy.

### Data analysis

The chromatograph of each automated sequence was visually checked. Multiple sequences were aligned using Clustal X [34] with default parameters, and additional manual editing to minimize gaps using GeneDoc program. Maximum-parsimony (MP) analysis was performed using the computer program PAUP ver. 4 beta 10 [35]. All characters were specified as unweighted and unordered, and gaps were not included in the analysis. A heuristic search using the Tree Bisection-Reconnection (TBR) option with MulTrees on, and ten replications of random addition sequences with the stepwise addition option were used to generate most-parsimonious trees. A strict consensus tree was generated from the obtained multiple parsimonious trees. Overall character congruence was estimated by the consistency index (CI), and the retention index (RI). The robustness of clades was inferred using bootstrap values calculated with 1000 replications [36].

In addition to maximum parsimony analysis, Bayesian analysis was also performed. Models of sequence evolution were tested for each data set using PhyML 3.0 program [37]. The general time-reversible (GTR) [38] substitution model led to a largest ML score for both *Rpb2* and *TrnL-F* compared to the other 7 substitution models (JC69, K80, F81, F84, HKY85, TN93 and custom), and was chosen in the Bayesian analysis using MrBayes 3.1 [39].

MrBayes 3.1 was run with the program’s standard setting of two analyses in parallel, each with four chains, and estimates convergence of results by calculating standard deviation of split frequencies between analyses. When 571,000 generations for *Rpb2* data and 1,788,000 generations for *TrnL-F* were reached, the standard deviation of split frequencies fell below 0.01. Samples were taken every 1000 generations under the GTR model with gamma-distributed rate variation across sites and a proportion of invariable sites. The first 25% of samples from each run were discarded as burn-in to ensure the stationarity of the chains. Bayesian posterior probability (PP) values were calculated from a majority rule consensus tree generated from the remaining sampled trees.

## Result

### Rpb2 sequence and phylogeny analysis

The amplified patterns from tetraploid *E*. *fibrosus* species showed two bands with sizes of approximately 900 bp and 1000 bp, respectively, which corresponded well with previous report by Sun et al. [17]. Extensive sequence variations were detected between the sequences from the **St** and **H** genomes. Sequence alignment with the **St**, **H**, **W** and **E** genomic species indicated that the 900 bp and 1000 bp amplified in tetraploid *E*. *fibrosus* corresponded to the size of sequences from **H** and **St** genome, respectively.

A total of 58 sequences were used for phylogenetic analysis, which included 18 sequences from 10 accessions of *E*. *fibrosus*, 22 sequences from 16 other *Elymus* species with **StH** genome, and 17 sequences from diploid species in the tribe Triticeae with **H**, **E**, **P**, **St** and **W** genomes, and one sequence from *Bromus sterilis* that was used as an outgroup.

**Fig 1:**
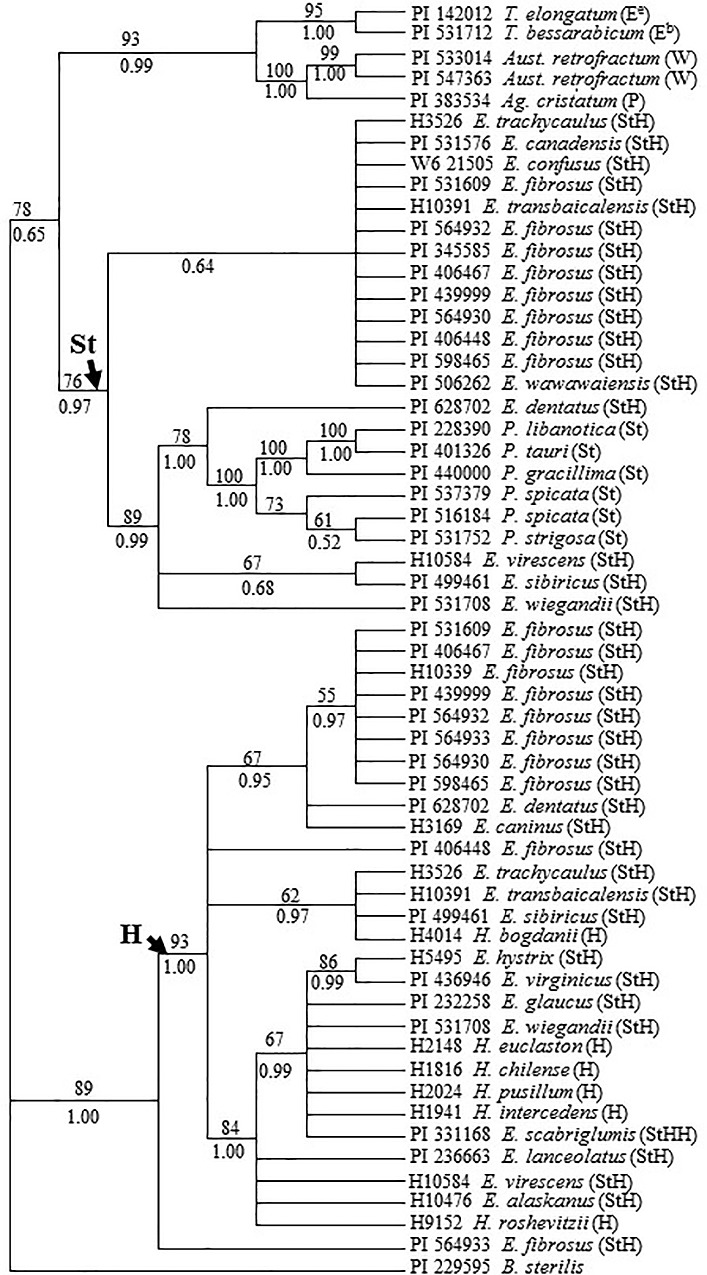
Strict consensus tree generated from 544 parsimonious trees based on *Rpb*2 sequence data which was conducted using heuristic search with TBR branch swapping. Numbers above and below branches are bootstrap values from MP and Bayesian posterior probability (PP) values, respectively. *Bromus sterilis* was used as an outgroup. Consistency index (CI) = 0.807, retention index (RI) = 0.929.

Total of 732 characters were used for phylogenetic analysis, including 385 constant, 122 parsimony uninformative and 225 parsimony informative characters. Maximum parsimony analysis of these 58 *Rpb2* sequences generated 544 equally most parsimonious trees with CI = 0.807 and RI = 0.929. The separated Bayesian analyses using GTR model resulted in identical trees with arithmetic mean log-likelihood values of -4011.69 and -4014.63. The tree topology generated by Bayesian analyses using the GTR model is similar to those generated by maximum parsimony. Consensus strict tree generated from maximum parsimonious trees with Bayesian PP and maximum parsimony bootstrap (1000 replicates) value is shown in Fig 1.

Two distinct copies of sequences were obtained for 8 out of 10 tetraploid *E*. *fibrosus* accessions that were amplified and sequenced (Table 1). Phylogenetic analysis well separated the two copies of sequences from each accession into two different clades, one in the **H** genome clade, another in the **St** genome clade with exception of two sequences from the accession PI 564933 (Fig 1). Of two copies of sequences from accession PI 564933 recovered, one was grouped into the **H** clade in 93% bootstrap support and 1.00 PP, another was sister to the **H** clade in 89% bootstrap support and 1.00 PP (Fig 1). Only one copy of sequence each from accession H10339 and PI 345585 was recovered. The sequences from the accession H10339 was placed into the **H** clade, while the sequence from PI 345585 was grouped into the **St** clade.

Within the **St** clade, all sequences from *E*. *fibrosus* were grouped together with the sequences from *E*. *canadensis*, *E*. *confusus*, *E*. *trachycaulus*, *E*. *transbaicalensis* and *E*. *wawawaiensis* in a support of 0.64 PP. The sequences from other species formed a subclade in 89% bootstrap and 0.99 PP support, included in which are the sequences from diploid **St** genome species and tetraploid *E*. *dentatus*, *E*. *sibiricus*, *E*. *virescens* and *E*. *wiegandii*. Within the **H** clade, all sequences of *E*. *fibrosus* accessions except the one from accession PI 406448 were placed together in 55% bootstrap and 0.97 PP support. The sequences from *E*. *caninus* and *E*. *dentatus* are sister to the *E*. *fibrosus* group in 67% bootstrap support and 0.95 PP.

### TrnL-F analysis

**Fig 2:**
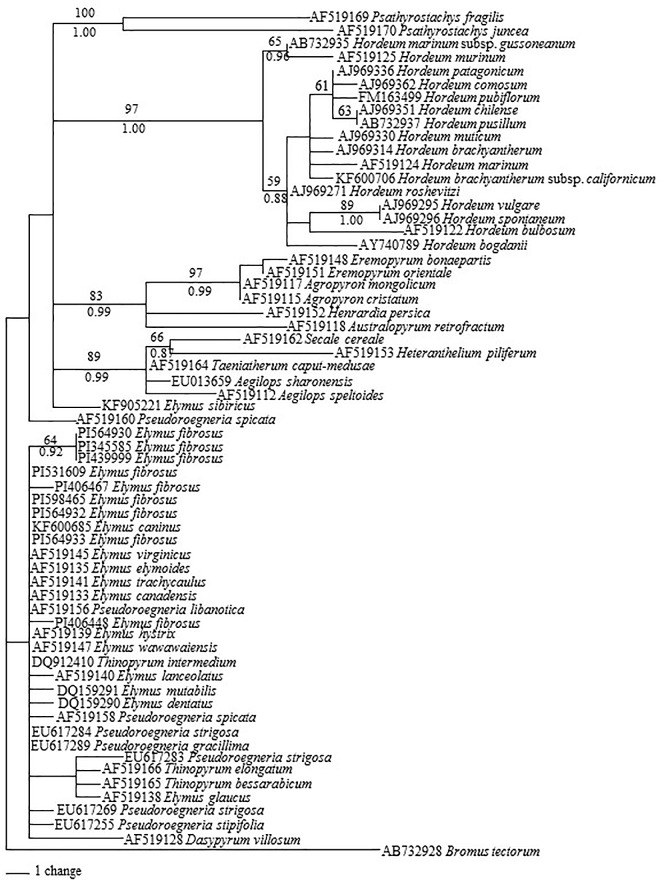
One of the 124 parsimonious trees derived from *TrnL-F* sequence data was conducted using heuristic search with TBR branch swapping. Numbers above and below branches are bootstrap values from MP and Bayesian posterior probability (PP) values, respectively. *Bromus tectorum* was used as an outgroup. Consistency index (CI) = 0.903, retention index (RI) = 0.951.

Maximum parsimonious analysis of 63 *TrnL-F* sequences was performed using *B*. *tectorum* as an outgroup. Total of 838 characters were used for phylogenetic analysis, of which 739 were constant, and 42 were parsimony informative. Maximum parsimonious analysis generated 124 most parsimonious trees with a CI = 0.903 (excluding uninformative characters) and RI = 0.951. The Bayesian analyses using GTR model resulted in identical trees with arithmetic mean log-likelihood values of -2232.85 and -2235.11. One of the maximum parsimonious tree is shown in Fig 2 with BS values from maximum parsimonious analysis and PP value from Bayesian analysis.

The phylogenetic tree showed several obvious groups. All sequences from *Hordeum* species were grouped together with the sequences from *Psathyrostachys*. The sequences from **H** genome were well separated from the sequences from **St** genome species. All sequences from *E*. *fibrosus* were grouped together with diploid *Pseudoroegneria* species (**St** genome), *Dasypyrum villosum* (**V)**. The sequences also included in this group were from tetraploid *Elymus* species and *Thinopyrum intermedium*.

## Discussion

Cytologically, *Elymus fibrosus* was considered as a tetraploid containing the genomes of a *Pseudoroegneria* species (**St** genome) and a wild *Hordeum* species (**H** genome) [1]. The phylogenetic analysis based on *TrnL-F* data here grouped all sequences from *E*. *fibrosus* with the sequences from diploid *Pseudoroegneria* species (**St** genome), *Dasypyrum villosum* (**V)**, other *Elymus* species and *Thinopyrum intermedium* together (Fig 2). This grouping is not exceptional. A study using combined cpDNA restriction sites, rpoA sequences, and tRNA spacer sequences also grouped several North American *Elymus* species with *Pseudoroegneria* (**St**) and *Dasypyrum* (**V**) [9]. It has been suggested maternal genome of *Elymus* species might be donated by three possible donors: *Pseudoroegneria*, *Dasypyrum* or *Thinopyrum* [40]. The study on the genomic constitution and evolution of *Thinopyrum intermedium* using *TrnL-F* placed the sequences from *Pseudoroegneria* (**St**), *Dasypyrum* (**V**) and *Thinopyrum intermedium* in a clade [41]. Our phylogenetic analysis of *E*. *trachycaulus* (**StH**) also grouped the *TrnL-F* sequences from *E*. *trachycaulus* with the sequences from *Pseudoroegneria* (**St**), *Dasypyrum* (**V**) and *Thinopyrum* (**E**) together [22]. cpDNA data on diploid species in Triticeae revealed a close relationship among *Pseudoroegneria* (**St**), *Dasypyrum* (**V**) and *Thinopyrum* (**E**) [42]. The *TrnL-F* sequence from *Thinopyrum intermedium* chloroplast that was placed in this clade (Fig 2) was downloaded from Mahelka et al. [43], and was from the **St** genome since *Th*. *intermedium* is hexaploid species with the **St** genome donated by *Pseudoroegneria* (**St**) [41]. The chloroplast sequences from *Hordeum* species were well separated from the sequences from *E*. *fibrosus* (Fig 2). The presence of *Pseudoroegneria*-derived chloroplast sequences is consistent with the nuclear gene *Rpb2* sequence data, in which the two distinct copies of *Rpb2* sequences from each *E*. *fibrosus* were well separated into *Pseudoroegneria* and *Hordeum* clades (Fig 1). Taking all into consideration, we suggested *Pseudoroegneria* as the likeliest maternal progenitor of *E*. *fibrosus*. It was documented that *Pseudoroegneria* (**St**) was the maternal parent of polypoids containing the **St** nuclear genome in combination with other genomes [7], which has been reported in numerous studies [8–13, 21]. However, a study also suggested that not only *Pseudoroegneria* (**St**) but also *Agropyron* (**P**) are the likely maternal genome donors to *Kengyilia* (**StYP**) species [44]. The *Pseudoroegneria* as the likeliest maternal progenitor of *E*. *fibrosus* should be verified by additional chloroplast sequences.

Two distinct copies of *Rpb2* sequences each from 8 out of ten accessions of *E*. *fibrosus* were discovered. Phylogenetic analysis well separated the two distinct copies of sequences from each accession into **St** and **H** clades except the two distinct copies of sequences from the accession PI 564933, confirming that seven accessions (PI 439999, PI 564930, PI 598465, PI 406467, PI 531609, PI 406448, and PI 564932) of *E*. *fibrosus* have the **StH** genomic constitution, and supporting the cytological evidence on the genomic constitution of this species [1]. However, one copy from the accession PI 564933 was placed into the **H** clade, while another copy is sister to the **H** clade in phylogenetic analysis (Fig 1). This is unexpected, but has also been reported in other *Elymus* species. In *E*. *trachycaulus* species, two/three **H**-like *Pepc* sequences from some accessions were obtained without **St** copy sequence from these accessions. Phylogenetic analysis clearly separated the two distinct copies of sequences from each accession into **H1** and **H2** clades [22]. In some accessions of **StStHH** allotetraploids *E*. *lanceolatus* and *E*. *wawawaiensis*, each has two different copies of sequences from the **St** genome, including one *P*. *spicata–*like sequence and another *P*. *strigosa*–like sequence [18]. As what has been discussed in Zuo et al [22], gene introgression from *Hordeum* into *E*. *fibrosus* following polyploidization; incomplete concerted evolution which incompletely homogenized **St** copy of *Rpb2* toward second **H** copy of *Rpb2*, and concerted evolution even it is very common for highly repetitive nuclear sequences [45, 46] could occur for some low-copy nuclear genes, are the more likely reasons causing two distinct **H-**like copies of sequences present in the accession PI 564933 of allotetraploid *E*. *fibrosus*. It cannot be excluded that homoeologous rearrangements in *Brassica napus* [47, 48] and exchange among homoeologous chromosomes [49] might promote convergent evolution after polyploidization, which could result in two original distinct copies of sequences similar to each other. Only one copy of sequence each from accession H10339 and PI 345585 was recovered. If no bias in cloning or PCR amplification, there is 99.9% chance of obtaining at least one copy of each of the two ancestral allelic types for the allotetraploid [50]. This might be due to mutation in the primers region causing failure of amplification of the “missing” gene copy. Another possibility might be genome convergent evolution in allopolyploids as discussed above.Allozyme, RAPD and microsatellite studies on *E*. *fibrosus* indicated that although there are differences in the amount of genetic diversity detected by allozyme, RAPD and microsatellite analyses, this species possesses a very low amount of genetic variation in its populations [15, 16]. However, *E*. *caninus*, *E*. *alaskanus*, and *E*. *trachycaulus* with **StH** genomic constitution have similar ecological preferences and breeding systems with *E*. *fibrosus*, genetic diversity of *E*. *caninus* [23, 24], *E*. *alaskanus* [25–28], and *E*. *trachycaulus* complex [29, 30] showed much higher level than that in *E*. *fibrosus*. Genetic diversity within plant species is complex results of many factors such as abiotic ecological forces, biotic agents, and species characteristics including population size, breeding system, migration and dispersal [51, 52]. Multiple origins are often considered as one of potential sources for increasing genetic variation in polyploids [31]. Our previous studies have shown that **St** genome in *E*. *caninus* has two distinct origins from either **St**_**1**_ and/or **St**_**2**_, and that *P*. *spicata* and *P*. *stipifolia* are the most likely donors of the **St**_**2**_ genome copies. The sequences data also indicated multiple origins of the **H** genome in *E*. *caninus* [21]. Zou et al. [22] indicated that the **St** genome in *E*. *trachycaulus* was originated from either *P*. *strigosa*, *P*. *stipifolia*, *P*. *spicata* or *P*. *geniculate*, and that the **H** genome in *E*. *trachycaulus* was contributed by multiple sources. However, phylogenetic analysis of *Rpb*2 sequences here revealed that all **St** copies of sequences from *E*. *fibrosus* were grouped together, and all **H** copy sequences of *E*. *fibrosus* accessions except the accession PI 406448 were also placed together, suggesting that **St** and **H** genome in *E*. *fibrosus* each was more likely originated from single gene pool, which might be one of the reasons causing genetic diversity in *E*. *fibrosus* lower than those in *E*. *caninus* and *E*. *trachycaulus*.

## Supporting Information

S1 File RPB2 sequences used in phylogenetic analysis. (PDF)

S2 File TrnL–trnF sequences used for phylogenetic analysis. (PDF)
