# Supplementary material for: Origin and Evolution of Allopolyploid Wheatgrass Elymus fibrosus (Schrenk) Tzvelev (Poaceae: Triticeae) Reveals the Effect of Its Origination on Genetic Diversity
Source: PLoS One. 2016 Dec 9;11(12):e0167795. doi: 10.1371/journal.pone.0167795 (PMC5147983; doi:10.1371/journal.pone.0167795)
Supplement: S1 File — (PDF) [file pone.0167795.s001.pdf]

S1_file. RPB2 sequences used in phylogenetic analysis

```
E142012     AACATTCAGCATGTAATTTT-GAGACCTGGATCTCATTACA-AATCACATACCAGCAATTAATGGCTTTCTTCTTAAA
E531712     AACATTCAGCATGTAATTTT-GAGACCTGGATCTCATTAAA-AATCACATACCAGCACTTAATGGCTTTCTTCTTAAC
W533014     AACATTCAGCATGTAATTTT-GAGACCTGAATGTCATTAAA-AATCACATACCAGCAATAAATGACTTTCTT---AGC
W547363     AACATTCAGCATGTAATTTT-GAGACCTGAATGTCATTAAA-AATCACATACCAGCAATAAATGACTTTCTT---AGC
P383534     --CATTCAGCATGTAATTTT-GAGACCTGAATGTCATTAAA-AATCACATGCCAGCAAGAAATGACTTTCTT---AAC
PI531609U   AACATTCAGCATGTAATTTT-GGGACATGAATGTCGTTAAA-AATCACATACCGGCAATAAATGACTTTCTT---AAC
PI406467L   AACATTCAGCATGTAATTTT-GGGACATGAATGTCGTTAAA-AATCACATACCGGCAATAAATGACTTTCTT---AAC
H10339      AACATTCAGCATGTAATTTT-GGGACATGAATGTCGTTAAA-AATCACATACCGGCAATAAATGACTTTCTT---AAC
PI439999L   AACATTCAGCATGTAATTTT-GGGACATGAATGTCGTTAAA-AATCACATACCGGCAATAAATGACTTTCTT---AAC
PI564932U   AACATTCAGCATGTAATTTT-GGGACATGAATGTCGTTAAA-AATCACATACCGGCAATAAATGACTTTCTT---AAC
PI564933L   AACATTCAGCATGTAATTTT-GGGACATGAATGTCGTTAAA-AATCACATACCGGCAATAAATGACTTTCTT---AAC
PI564930U   AACATTCAGCATGTAATTTT-GGGACATGAATGTCGTTAAA-AATCACATACCGGCAATAAATGACTTTCTT---AAC
PI598465U   AACATTCAGCATGTAATTTT-GGGACATGAATGTCGTTAAA-AATCACATACCGGCAATAAATGACTTTCTT---AAC
PI406448U   AACATTCAGCATGTAATTTT-GGGACATGAATGTCGTTAAA-AATCACATACCGGCAATAAATGACTTTCTT---AAC
628702L     AACATTCAGCATGTAATTTTTGGGACATGAATGTCGTTAAA-AATCACATACCGGCAATAAATGACTTTCTT---AAC
H3169L      AACATTCAGCATGTAATTTT-GGGACATGAATGACGTTAAA-AATCACATACCGGCAATAAATGACTTTCTT---AAC
H3526L      --CATTCAGCATGTAATTTT-GGGACATGAATGTCGTTAAA-AATCACATACCGGCAATAAATGACTTTCTT---AAC
H10391L     ------CAGCATGTAATTTT-GGGACATGAATGTCGTTAAA-AATCACATACCGGCAATAAATGACTTTCTT---AAC
499461L     AACATTCAGCATGTAATTTT-GGGACATGAATGTCGTTAAA-AATCACATACCGGCAATAAATGACTTTCTT---AAC
H4014       AACATTCAGCATGTAATTTT-GGGACATGAATGTCGTTAAA-AATCACATACCGGCAATAAATGACTTTCTT---AAC
H5495one    AACATTCAGCATGTAATTTT-AAGACCTGAATGTCGTTAAA-AATCACATGCCGGCAATAAATGACTTTCTT---AAC
436946      AACATTCAGCATGTAATTTT-AAGACCTGAATGTCGTTAAA-AATCACATGCCGGCAATAAATGACTTTCTT---AAC
232258U     AACATTCAGCATGTAATTTT-GGGACCTGAATGTCGTTAAA-AATCACATGCCGGCAATAAATGACTTTCTT---AAC
531708L     AACATTCAGCATGTAATTTT-GGGACCTGAATGTCGTTAAA-AATCACATGCCGGCAATAAATAACTTTCTT---AAC
H2148       AACATTCAGCATGTAATTTT-GGGACCTGAATGTCGTTAAA-AATCACATGCCGGCAATAAATGACTTTCTT---AAC
H1816       AACATTCAGCATGTAATTTT-GGGACCTGAATGTCGTTAAA-AATCACATGCCGGCAATAAATGACTTTCTT---AAC
H2024       AACATTCAGCATGTAATTTT-GGGACCTGAATGTCGTTAAA-AATCACATGCCGGCAATAAATGACTTTCTT---AAC
H1941       AACATTCAGCATGTAATTTT-GGGACCTGAATGTCGTTAAA-AATCACATGCCGGCAATAAATGACTTTCTT---AAC
331168L     ---ATTCAGCATGTAATTTT-GGGACCTGAATGTCATTAAA-AATCACATGCCGGCAATAAATGACTTTCTT---AAC
236663L     ---ATTCAGCATGTAATTTT-GGGACCTGAATGTCGTTAAA-AATCACATACCAGCAATAAATGACTTTCTT---AAC
H10584L     --TAATCAGCATGTAATTTTTGGGACCTGAATGTCGTTAAA-AATCACATACCAGCAATAAATGACTTTCTT---AAC
US9601      AACATTCAGCATGTAATTTT-GGGACCTGAATGTCGTTAAA-AATCACATACCAGCAATAAATGACTTTCTT---AAC
H9152       AACATTCAGCATGTAATTTT-GGGACCTGAATGTCGTTAAA-AATCACATACCAGCAATAAATGACTTTCTT---AAC
PI564933U   AACATTCAGCATGTAATTTT-GAGAC------GTCGTTAAA-AATCACATAGCAGCACTAAATGACTTCCTT---AAC
PI229595    ------------------------------------------------------------------------------
H3526U1     AACATTCAGCATGGTATTTT-GAGAC------GTCGTTAAA-ATTCACATAGCAGCACTAATTGACTTCCTT---AAC
GQ867861    AACATTCAGCATGTAATTTT-GAGAC------GTCGTTAAA-AATCACATAGCAGCACTAAATGACTTCCTT---AAC
GQ867864    AACATTCAGCATGTAATTTT-GAGAC------GTCGTTAAA-AATCACATAGCAGCACTAAATGACTTCCTT---AAC
PI531609L   AACATTCAGCATGTAATTTT-GAGAC------GTCGTTAAA-AATCACATAGCAGCACTAAATGACTTCCTT---AAC
H10391U     AACATTCAGCATGTAATTTT-GAGAC------GTCGTTAAA-AATCACATAGCAGCACTAAATGACTTCCTT---AAC
PI564932L   AACATTCAGCATGTAATTTT-GAGAC------GTCGTTAAA-AATCACATAGCAGCACTAAATGACTTCCTT---AAC
PI345585U   AACATTCAGCATGTAATTTT-GAGAC------GTCGTTAAA-AATCACATAGCAGCACTAAATGACTTCCTT---AAC
PI406467U   AACATTCAGCATGTAATTTT-GAGAC------GTCGTTAAA-AATCACATAGCAGCACTAAATGACTTCCTT---AAC
PI439999U   AACATTCAGCATGTAATTTT-GAGAC------GTCGTTAAA-AATCACATAGCAGCACTAAATGACTTCCTT---AAC
PI564930L   AACATTCAGCATGTAATTTT-GAGAC------GTCGTTAAA-AATCACATAGCAGCACTAAATGACTTCCTT---AAC
PI406448L   AACATTCAGCATGTAATTTT-GAGAC------GTCGTTAAA-AATCACATAGCAGCACTAAATGACTTCTTA----AC
PI598465L   AACATTCAGCATGTAATTTT-GAGAC------GTCGTTAAA-AATCACATAGCAGCACTAAATGACTTCCTT---AAC
EU187442    AACATTCAGCATGTAATTTT-GAGAC------GTCGTTAAA-AATCACATAGCAGCACTAAATGACTTCCTT---AAC
628702U     -----------TGTAATTTT-GAGAC------GTCGTTAAA-AATCACATAACAGCACTAAATGACATCCTT---AAC
H10584U     ---------------ATTTT-GAGAC------GTCGTTAAA-AATCACATAGCAGCACTAAATGACTTCCTT---AAC
531708U     ------------------------------ACGTCGTTACAAATCACATAGCAGCACTAAATGACTTCCTT---AAC
499461u1    -------------------------------------TAAA--ATCACATAGCAGCACTAAATGACTTCCTT--AA-C
PI228390    ----------------------------------------------------------CCAAGAGGCGA-TTCGANCC
PI401326    ------------------------------------------------------------AAGGAGCGA-TTCGAACC
PI420842    ------------GCGATTCGAACCACACCAACTTCAGTGTG-CAGGACACATCATTCCCCAAAGAGCGAATTCGAACC
PI537379    AGTTCCTCAAGAGCGATTCGA-CCACACCAACTTCAGTGTG-CAGGACACATCATTCCCCAAAAAGAGTACCTTAACC
PI516184    CATTCC-CAAGAGCGATTCGAACCACACCAACTTCAGTGTG-CAGGACACATCATTCCCCTAAGTGAGTACCTTAACC
```

```
PI531752     -------------GAATGCGAACCACTCCAACTTCAGTGTG-CAGGACACATCATTCCCCTAAGTGAGTTCCTTAACA


E142012      ATGTTCTATGCTAAAGAAATTCAAGATGTAC--------------TATCCAAAATGA-TCTTTACTTTGGA-------
E531712      ATGTTCTATGCTAAAGAAATTCAAGATGTACATGTTTACCATACATCTTGAAAATGA-TCTTTACTTTGGA-------
W533014      ATGTTCTATGCTAAAGAAATTCAAGATGTACATGTTTACCATACATCTTGAAAATGA-TCTTTACTTTGGA-------
W547363      ATGTTCTATGCTAAAGAAATTCAAGATGTACATGTTTACCATACATCTTGAAAATGA-TCTTTACTTTGGA-------
P383534      ATATTCTATGCTAAAGAAATTCAAG--------------------TCTTGAAAATGA-TCTTTACTTTGGA-------
PI531609U    ATGTTC-------------------------------ACCATACATCTTGAAAAAGA-TCTTTACTTTGCA-------
PI406467L    ATGTTC-------------------------------ACCATACATCTTGAAAAAGA-TCTTTACTTTGCA-------
H10339       ATGTTC-------------------------------ACCATACATCTTGAAAAAGA-TCTTTACTTTGCA-------
PI439999L    ATGTTC-------------------------------ACCATACATCTTGAAAAAGA-TCTTTACTTTGCA-------
PI564932U    ATGTTC-------------------------------ACCATACATCTTGAAAAAGA-TCTTTACTTTGCA-------
PI564933L    ATGTTC-------------------------------ACCATACATCTTGAAAAAGA-TCTTTACTTTGCA-------
PI564930U    ATGTTC-------------------------------ACCATACATCTTGAAAAAGA-TCTTTACTTTGCA-------
PI598465U    ATGTTC-------------------------------ACCATACATCTTGAAAAAGA-TCTTTACTTTGCA-------
PI406448U    ATGTTC-------------------------------AC---------------------------------------
628702L      ATGTTC-------------------------------ACCATACATCTTGAAAAAGA-TCTTTACTTTGCA-------
H3169L       ATGTTC-------------------------------ACCATACAACTTGAAAAAGA-TCTTTACTTTGCA-------
H3526L       ATGTTC-------------------------------ACCATACATCTTGAAAATGA-TCTTTACTTTGCA-------
H10391L      ATGTTC-------------------------------ACCATACATCTTGAAAATGA-TCTTTACTTTGCA-------
499461L      ATGTTC-------------------------------ACCATACATCTTGAAAATGA-TCTTTACTTTGCA-------
H4014        ATGTTC-------------------------------ACCATACATCTTGAAAATGA-TCTTTACTTTGCA-------
H5495one     ATGTTC-------------------------------ACCATACATCTTGAAAAAGA-TCTTTACTTTGCA-------
436946       ATGTTC-------------------------------ACCATACATCTTGAAAAAGA-TCTTTACTTTGCA-------
232258U      ATGTTC-------------------------------ACCATACATCTTGAAAAAGA-TCTTTACTTTGCA-------
531708L      ATGTTC-------------------------------ACCATACATCTTGAAAAAGA-TCTTTACTTTGCA-------
H2148        ATGTTC-------------------------------ACCATACATCTTGAAAAAGA-TCTTTACTTTGCA-------
H1816        ATGTTC-------------------------------ACCATACATCTTGAAAAAGA-TCTTTACTTTGCA-------
H2024        ATGTTC-------------------------------ACCATACATCTTGAAAAAGA-TCTTTACTTTGCA-------
H1941        ATGTTC-------------------------------ACCATACATCTTGAAAAAGA-TCTTTATTTTGCA-------
331168L      ATGTTC-------------------------------ACCATACATCTTGAAAAAGA-TCTTTACTTTGCA-------
236663L      ATGTTC-------------------------------ACCATACATCTTGAAAATGA-TCTTTACTTTGCA-------
H10584L      ATGTTC-------------------------------ACCATACATCTTGAAAATGA-TCTTTACTTTGCA-------
US9601       ATGTTC-------------------------------ACCATACATCTTGAAAATGA-TCTTTACTTTGCA-------
H9152        ATGTTC-------------------------------ACCATACATCTTGAAAATGA-TCTTTACTTTGCA-------
PI564933U    ATGTTCTATGCTAAAGAATTCAAGATGTATATGTTT-ACCATACATCTTGAAAATGA-TCTTTACTTTGGA-------
PI229595     ------------------------------------------------------------------------------
H3526U1      ATGTTCTATGCTATAGAATTCCAGA-TGTATATGTTTACCATACATCTTGAAAATGA-CCTTTACTTTGGA-------
GQ867861     ATGTTCTATGCTAAAGAA-TTCAAGATGTATATGTTTACCATACATCTTGAAAATGA-TCTTTACTTTGGA-------
GQ867864     ATGTTCTATGCTAAAGAA-TTCAAGATGTATATGTTTACCATACATCTTGAAAATGA-CCTTTACTTTGGA-------
PI531609L    ATGTTCCATGCTAAAGAG-TTCAAGATGTATATGTTTACCATACATCTTGAAAATGA-TCTTTACTTTGGA-------
H10391U      ATGTTCTATGCTAAAGAA-TTCAAGATGTATATGTTTACCATACATCTTGAAAATGA-TCTTTACTTTGGA-------
PI564932L    ATGTTCTATGCTAAAGAA-TTCAAGATGTATATGTTTACCATACATCTTGAAAATGA-TCTTTACTTTGGA-------
PI345585U    ATGTTCTATGCTAAAGAA-TTCAAGATGTATATGTTTACCATACATCTTGAAAATGA-TCTTTACTTTGGA-------
PI406467U    ATGTTCTATGCTAAAGAA-TTCAAGATGTATATGTTTACCATACATCTTGAAAATGA-TCTTTACTTTGGA-------
PI439999U    ATGTTCTATGCTAAAGAA-TTCAAGATGTATATGTTTACCATACATCTTGAAAATGA-TCTTTACTTTGGA-------
PI564930L    ATGTTCTATGCTAAAGAA-TTCAAGATGTATATGTTTACCATACATCTTGAAAATGA-TCTTTACTTTGGA-------
PI406448L    ATGTTCTATGCTAAAGAA-TTCAAGATGTATATGTTTACCATACATCTTGAAAATGA-TCTTTACTTTGGA-------
PI598465L    ATGTTCTATGCTAAAGAA-TTCAAGATGTATATGTTTACCATACATCTTGAAAATGA-TCTTTACTTTGGA-------
EU187442     ATGTTCTATGCTAAAGAA-TTCAAGATGTATATGTTTACCATACATCTTGAAAATGA-CCTTCACTTTGGA-------
628702U      ATGTTCTATGCTAAAGAA-TTCAAGATGTATATGTTTACCATACATCTTGAAAATGA-TCTTTACTTTGGA-TGGTAT
H10584U      ATGTTCTATGCTAAAGAA-TTCAAGATGTATATGTTTACCATACATCTTGAAAATGA-CCTTTACTTTGGA-TGGTAT
531708U      ATGTTCTATGCTAAAGAA-TTCAAGATGTATATGTTTACCATACATCTTGAAAATGA-TCTTTACTTTGGA-TGGTAT
499461u1     ATGTTCTATGCTAAAGAA-TTCAAGATGTATATGTTTACCATACATCTTGAAAATGA-CCTTTACTTTGGAT-GGTAT
PI228390     A--CTCCAACTTCAGTGTGCAGGACACATCATTCCCCTAAGTGAGTTTCTTAACATGTTCTCTGCTAAAGAATTCAAG
PI401326     A--CTCCAACTTCAGTGTGAAGGACACATCATTCCCCTAAATGAGTTTCTTAACATGTTCTATGCTAAAGAATTCAAG
PI420842     A--CACCAACTTCAGTGTGTAGGAGACATCTTTCTCCAAAAAGCGAATTCTAACCA--CACCTACTTCAGGGTGGGGA
PI537379     ---TGTTAACTGCTGTGTGTTCAAGATGTATATGTTCACCATACATCTTGAAAATGA-TCTTTACTTTGGA--TGGGA
PI516184     ---TGTTCAACGCTGTGAATTCAAGATGTATATGTTTACCATACATCTTGAAAATGA-TCTTTACTTTGGA--TGGTA
PI531752     ----TGCCTCTTCTGAGAATTGAAGATGTCTATGTTTACCATACATCTTGAAAATGA-TCTTTACTTTGGA--TGGGA
```

E142012	-TA-GCA---CCAGATATCAAACAAAGTTAAGGTTGCA
E531712	-TA-GTA---GCAGATATCAAACTAA-TTAGGGTTGCA
W533014	-TA-GTA---CCAGATATCGAACAAAATTAGGGTTGCA
W547363	-TA-GTA---CCAGATATCGAACAAAATTAGGGTTGCA
P383534	-TA-GTA---CCAGATAACGAACAAAATTAGGGTTGCA
PI531609U	-TA-GTA---------------------------------------AGAACCAGATATCAAACTAAGTCAGGATTGCA
PI406467L	-TA-GTA---------------------------------------AGAACCAGATATCAAACTAGGTCAGGATTGCA
H10339	-TA-GTA---------------------------------------AGAACCAGATATCAAACTAAGTCAGGATTGCA
PI439999L	-TA-GTA---------------------------------------AGAACCAGATATCAAACTAAGTCAGGATTGCA
PI564932U	-TA-GTA---------------------------------------AGAACCAGATATCAAACTAAGTCAGGATTGCA
PI564933L	-TA-GTA---------------------------------------AGAACCAGATATCAAACTAAGTCAGGATTGCA
PI564930U	-TA-GTA---------------------------------------AGAACCAGATATCAAACTAAGTCAGGATTGCA
PI598465U	-TA-GTA---------------------------------------AGAACCAGATATCAAACTAAGTCAGGATTGCA
PI406448U	---TAAGTCAGGATTGCA
628702L	-TA-GTA---------------------------------------AGTACCAGATATCAAACTAAGTCAGGATTGCA
H3169L	-TA-GTA---------------------------------------AGTACCAGATATCAAACCAAGTCAGGATTGCA
H3526L	-TA-GTA---------------------------------------AGTACCAGATATCAAACTAAGTCAGGATTGCA
H10391L	-TA-GTA---------------------------------------AGTACCAGATATCAAACTAAGTCAGGATTGCA
499461L	-TA-GTA---------------------------------------AGTACCAGATATCAAACTAAGTCAGGATTGCA
H4014	-TA-GTA---------------------------------------AGTACCAGATATCAAACTAAGTCAGGATTGCA
H5495one	-TA-GTA---------------------------------------AGTACCAGATATCAAACTAAGTCAGGATTGCA
436946	-TA-GTA---------------------------------------AGTACCAGATATCAAACTAAGTCAGGATTGCA
232258U	-TA-GTA---------------------------------------AGTACCAGATATCAAACTAAGTCAGGATTGCA
531708L	-TA-GTA---------------------------------------AGTACCAGATATCAAACTAAGTCAGGATTGCA
H2148	-TA-GTA---------------------------------------AGTACCAGATATCAAACTAAGTCAGGATTGCA
H1816	-TA-GTA---------------------------------------AGTACCAGATATCAAACTAAGTCGGGATTGCA
H2024	-TA-GTA---------------------------------------AGTACCAGATATCAAACTAAGTCAGGATTGCA
H1941	-TA-GTA---------------------------------------AGTACCAGATATCAAACTAAGTCAGGATTGCA
331168L	-TA-GTA---------------------------------------AGTACCAGATATCAAACTAAGTCAGGATTGCA
236663L	-TA-GTA---------------------------------------AGTACCAGATATCAAACTAAGTCAGGATTGCA
H10584L	-TA-GTA---------------------------------------AGTACCAGATATCAAACTAAGTCAGGATTGCA
US9601	-TA-GTA---------------------------------------AGTACCAGATATCAAACTAAGTCAGGATTGCA
H9152	-TA-GTA---------------------------------------AGTACCAGATATCAAACTAAGTCAGGATTGCA
PI564933U	-TG-GTATCTAGTGTCAAAAACGCTCTTATATTATGGGACGGAGGGAGTACAAGATATAAAATAAAGTTAGGATTGCA
PI229595	--
H3526U1	-TG-GTATCTAGTGTCAAAAACGCTCTTATATTATGGGACGGAGGGAGTACAAGATATAAAATTAAGTTAGGATTGCA
GQ867861	-TG-GTATCTAGTGTCAAAAACGCTCTTATATTATGGGACGGAGGGAGTACAAGATATAAAATAAAGTTAGGATTGCA
GQ867864	-TG-GTATCTAGTGTCAAAAACGCTCTTATATTATGGGACGGAGGGAGTACAAGATATAAAATAAAGTTAGGATTGCA
PI531609L	-TG-GTATCTAGTGTCAAAAACGCTCTTATATTATGGGACGGAGGGAGTACAAGATATAAAATAAAGTTAGGATTGCA
H10391U	-TG-GTATCTAGTGTCAAAAACGCTCTTATATTATGGGACGGAGGGAGTACAAGATATAAAATAAAGTTAGGATTGCA
PI564932L	-TG-GTACCTAGTGTCAAAAACGCTCTTATATTATGGGACGGAGGGAGTACAAGATATAAAATAAAGTTAGGATTGCA
PI345585U	-TG-GTATCTAGTGTCAAAAACGCTCTTATATTATGGGACGGAGGGAGTACAAGATATAAAATAAAGTTAGGATTGCA
PI406467U	-TG-GTATCTAGTGTCAAAAACGCTCTTATATTATGGGACGGAGGGAGTACAAGATATAAAATAAAGTTAGGATTGCA
PI439999U	-TG-GTATCTAGTGTCAAAAACGCTCTTATATTATGGGACGGAGGGAGTACAAGATATAAAATAAAGTTAGGATTGCA
PI564930L	-TG-GTATCTAGTGTCAAAAACGCTCTTATATTATGGGACGGAGGGAGTACAAGATATAAAATAAAGTTAGGATTGCA
PI406448L	-TG-GTATCTAGTGTCAAAAACGCTCTTATATTATGGGACGGAGGGAGTACNAGATATAAAATAAAGTTAGGATTGCA
PI598465L	-TG-GTATCTAGTGTCAAAAACGCTCTTATATTATGGGACGGAGGGAGTACAAGATATAAAATAAAGTTAGGATTGCA
EU187442	-TG-GTATCTAGTGTCAAAAACGCTCTTATATTATGGGACGGAGGGAGTACAAGATATAAAATAAAGTTAGGATTGCA
628702U	CTA-GTGTCAAAAACG-CTCTTATATTATGGGACGG------AGGGAGTACAAGATATAAAATAAAGTTAGGATTGCA
H10584U	CTA-GTGTCAAAAACG-CTCTTATATTATGGGACGG------AGGGAGTACAAGATATAAAATAAAGTTAGGATTGCA
531708U	CTA-GTGTCAAAAACG-CTCTTATATTATGGGACGG------AGGGAGTACAAGATATAAAATAAAGTTAGGATTGCA
499461u1	CTA-GTGTCAAAAACGC-TCTTATATTATGGGACGG------AGGGAGTACAAGATATAAAATAAAGTTAGGATTGCA
PI228390	ATGTATATGTTTACCATACATCTTGAAAATGATCTTTACTTTGGATAGTACTAGATATCAAATAAAGTTAGGACTGCA
PI401326	ATGTATATGTTTACCATACATCTTGAAAATGATCTTTACTTTGGATAGTACTAGATATCAAATAAAGTTAGGACTGCA
PI420842	TCACATGTCTCCCCCG-ATAGTGTATTATGGAACCACCCCCTTCCCAGTGTTA------AAATAAAGC-AGGATTGCA
PI537379	TCTAGTGTCAAAAACG-CTCTTATATTATGGGACGG------AGGGAGTACAAGATATAAAATAAAGTTAGGATTGCA
PI516184	TCTAGTGTCAAAAACG-CTCTTATATTATGGGACGG------AGGGAGTACAAGATATAAAATAAAGTTAGGATTGCA
PI531752	TCTAGTGTCAAAAACG-CTCTTATATTATGGGACGG------AGGGAGTACTAGATATAAAATAAAGTTAGGATTGCA
E142012	TATGAAAGGGCAACAAAATCACATCAGTTTT-ATTAA-TGGAGTGCTCC-AATCTGTTAAAT-ATACAAATTATGCAG
E531712	TATGAAAGGGCAACAAAATCACATCAGTTTT-ATTAA-TGGAGTGCTCC-AATCTGTTAAAT-ATACAAATTATGCAG

W533014	TATGAAAGGGCAACAAAATCACATCAGTTTTTATTAAATGGAGTGCTCCCAATCTGTTAAAT-ATACAAATTATGCAG
W547363	TATGAAAGGGCAACAAAATCACATCAGTTTTTATTAAATGGAGTGCTCCCAATCTGTTAAAT-ATACAAATTATGCAG
P383534	TATGAAAGGGCAACAAAATCACATCAGTTTTTATTAAATGGAGTGCTCCCAATCTGTTAAAT-AAACAAATTATGCAG
PI531609U	TATGGAAGGGCGACAAAATCACATCAGTT-----------------------------AAAT-ATACAAAATATGCAG
PI406467L	TATGGAAGGGCAACAAAATCACATCAGTT-----------------------------AAAT-ATACAAAATATGCAG
H10339	TATGGAAGGGCAACAAAATCACATCAGTT-----------------------------AAAT-ATACAAAATATGCAG
PI439999L	TATGGAAGGGCAACAAAATCACATCAGTT-----------------------------AAAT-ATACAAAATATGCAG
PI564932U	TATGGAAGGGCAACAAAATCACATCAGTT-----------------------------AAAT-ATACAAAATATGCAG
PI564933L	TATGGAAGGGCAACAAAATCACATCAGTT-----------------------------AAAT-ATACAAAATATGCAG
PI564930U	TATGGAAGGGCAACAAAATCACATCAGTT-----------------------------AAAT-ATACAAAATATGCAG
PI598465U	TATGGAAGGGCAACAAAATCACATCAGTT-----------------------------AAAT-ATACAAAATATGCAG
PI406448U	TATGGAAGGGCAACAAAATCACATCAGTT-----------------------------AAAT-ATACAAAATATGCAG
628702L	TATGGAAGGGCAACAAAATCACATCAGTT-----------------------------AAAT-ATACAAAATATGCAG
H3169L	TATGGAAGGGCAACAAAATCACATCAGTT-----------------------------AAAT-ATACAAAATATGCAG
H3526L	TATGGAAGGGCAACAAAATCACATCAGTT-----------------------------AAAT-ATACAAAATATGCAG
H10391L	TATGGAAGGGCAACAAAATCACATCAGTT-----------------------------AAAT-ATACAAAATATGCAG
499461L	TATGGAAGGGCAACAAAATCACATCAGTT-----------------------------AAAT-ATACAAAATATGCAG
H4014	TATGGAAGGGCAACAAAATCACATCAGTT-----------------------------AAAT-ATACAAAATATGCAG
H5495one	TATGGAAGGGCAACAAAATCACATCAGTT-----------------------------AAAT-AT---------GCAG
436946	TATGGAAGGGCAGCAAAATCACATCAGTT-----------------------------AAAT-AT---------GCAG
232258U	TATGGAAGGGCAACAAAATCACATCAGTT-----------------------------AAAT-AT---------GCAG
531708L	TATGAAAGGGCAACAAAATCACATCAGTT-----------------------------AAAT-AT---------GCAG
H2148	TATGGAAGGGCAACAAAATCACATCAGTT-----------------------------AAAT-AT---------GCAG
H1816	TATGGAAGGGCAACAAAATCACATCAGTT-----------------------------AAAT-AT---------GCAG
H2024	TATGGAAGGGCAACAAAATCACATCAGTT-----------------------------AAAT-AT---------GCAG
H1941	TATGGAAGGGCAACAAAATCACATCAGTT-----------------------------AAAT-AT---------GCAG
331168L	GATGGAAGGGCAACAAAATCACATCAGTT-----------------------------AAAT-AT---------GCAG
236663L	TATGGAAGGGCAACAAAATCACATCAGTT-----------------------------AAAT-ATACAAAATATGCAG
H10584L	TATGGAAGGGCAACAAAATCACATCAGTT-----------------------------AAAT-ATACAAAATATGCAG
US9601	TATGGAAGGGCAACAAAATCACATCAGTT-----------------------------AAAT-ATACAAAATATGCAG
H9152	TATGGAAGGGCAACAAAATCACATCAGTT-----------------------------AAAT-ATACAAAATATGCAG
PI564933U	TATGGAAGGGCAACAAAATCACATCAGTTTT--ATTAATGGAGTGCTCCCAATGTGTTAAAG-ATACAAATTATGCAG
PI229595	--
H3526U1	TATGGAAGGGCCACAAAATCACATCAGTTTT--ATTAATGGAGTGCTCCCAATGTGCTAAAG-ATACAAATTATGCAG
GQ867861	TATGGAAGGGCAACAAAATCACATCAGTTTT--ATTAATGGAGTGCTCCCAATGTGTTAAAG-ATACAAATTATGCAG
GQ867864	TATGGAAGGGCAACAAAATCACATCAGTTTT--ATTAATGGAGTGCTCCCAATGTGTTAAAG-ATACAAATTATGCAG
PI531609L	TATGGAAGGGCAACAAAATCACATCAGTTTT--ATTAATGGAGTGCTCCCAATGTGTTAAAG-ATACAAATTATGCAG
H10391U	TATGGAAGGGCAACAAAATCACATCAGTTTT--ATTAATGGAGTGCTCCCAATGTGTTAAAG-ATACAAATTATGCAG
PI564932L	TATGGAAGGGCAGCAAAATCACATCAGTTTT--ATTAATGGAGTGCTCCCAATGTGTTAAAG-ATACAAATTATGCAG
PI345585U	TATGGAAGGGCAACAAAATCACATCAGTTTT--ATTAATGGAGTGCTCCCAATGTGTTAAAG-ATACAAATTATGCAG
PI406467U	TATGGAAGGGCAACAAAATCACATCAGTTTT--ATTAATGGAGTGCTCCCAATGTGTTAAAG-ATACAAATTATGCAG
PI439999U	TATGGAAGGGCAACAAAATCACATCAGTTTT--ATTAATGGAGTGCTCCCAATGTGTTAAAG-ATACAAATTATGCAG
PI564930L	TATGGAAGGGCAACAAAATCACATCAGTTTT--ATTAATGGAGTGCTCCCAATGTGTTAAAG-ATACAAATTATGCAG
PI406448L	TATGGAAGGGCAACAAAATCACATCAGTTTT--ATTAATGGAGTGCTCCCAATGTGTTAAAG-ATACAAATTATGCAG
PI598465L	TATGGAAGGGCAACAAAATCACATCAGTTTT--ATTAATGGAGTGCTCCCAATGTGTTAAAG-ATACAAATTATGCAG
EU187442	TATGGAAGGGCAACAAAATCACATCAGTTTT--ATTAATGGAGTGCTCCCAATGTGTTAAAG-ATACAAATTATGCAG
628702U	TATGGAAGGGCAACAAAATCACATCTGTTTT--ATTAATGGAGTGCTCCCAATGTGTTAAAG-ATACAAATTATGCAG
H10584U	TATGGAAGGGCAACAAAATCACATCAGTTTT--ATTAATGGAGTGCTCCCAACGTGTTAAAG-ATACAAATTATGCAG
531708U	TATGGAAGGGCAACAAAATCACATCAGTTTT--ATTAATGGAGTGCTCCCAATGTGTTAAAG-ATACAAATTATGCAG
499461u1	TATGGAAGGGCAACAAAATCACATCAGTTTT--ATTAATGGAGTGCTCCCAATGTGTTAAAGATACAAATTATGCAG
PI228390	TATGGAAGGGCAACAAAATCACATCAGTTTT--ATTAATGGAGTGCTCCCAATGTGTTAAAT-ATACAAATTCTGCAG
PI401326	TATGGAAGGGCAACAAAATCACATCAGTTTT--ATTAATGGAGTGCTCCCAATGTGTTAAAT-ATACAAATTCTGCAG
PI420842	TATGGAAGGGCAACAAAATCACATCAGTTTT--ATTAATGGAGTGCTCCCAATGTGTTAAAG-ATACAAATTATGCAG
PI537379	TATGGAAGGGCAACAAAATCACATCAGTTTT--ATTAATGGAGTGCTCCCAATGTGTTAAAG-ATACAAATTATGCAG
PI516184	TATGGAAGGGCAACAAAATCACATCAGTTTT--ATTAATGGAGTGCTCCCAATGTGTTAAAG-ATACAAATTATGCAG
PI531752	TATGGAAGGGCAACAAAATCACATCAGTTTT--ATTAATGGAGTGCTCCCAATGTGTTAAAG-ATACAAATTATGCAG

E142012	ACAATATATGTTTTTCAATACTCATATCCATTCTGCTACAATGCCAGCAGCTTTTAGCATGGAACAGGAGAAGCAGGG
E531712	CTAATATATGTTTTTCAATAC---TATCCATTCTGCTACAATGCCAGCAGCTTTTAGCATGAAACAGGAGAAGCAGGG
W533014	ATAATGTATGTTTTTCAATACTCATATCCATTTTGCTACGTTGCCAGCAGCTTTTAGCATGAAACAGGAGAAGCAGGG
W547363	ATAATGTATGTTTTTCAATACTCATATCCATTTTGCTACATTGCCAGCAGCTTTTAGCATGAAACAGGAGAAGCAGGG

```
P383534     ATAATATATGTTTTTCAATACTCATATCCATTCTGCTACATTGCCAGCAGCTTTTAGCATGAAACAGGAGAAGCAGGG
PI531609U   ATAATATATGTTTCCCAATACTCATATCTATTCTGCTACATTGCCAGCAGCTTTTAGAATGGAACAGGAGAAGCAAAG
PI406467L   ATAATATATGTTTCCCAATACTCATATCTATTCTGCTACATTGCCAGCAGCTTTTAGAATGGAACAGGAGAAGCAAAG
H10339      ATAATATATGTTTCCCAATACTCATATCTATTCTGCTACATTGCCAGCAGCTTTTAGAATGGAACAGGAGAAGCAAAG
PI439999L   ATAATATATGTTTCCCAATACTCATATCTATTCTGCTACATTGCCAGCAGCTTTTAGAATGGAACAGGAGAAGCAAAG
PI564932U   ATAATATATGTTTCCCAATACTCATATCTATTCTGCTACATTGCCAGCAGCTTTTAGAATGGAACAGGAGAAGCAAAG
PI564933L   ATAATATATGTTTCCCAATACTCATATCTATTCTGCTACATTGCCAGCAGCTTTTAGAATGGAACAGGAGAAGCAAAG
PI564930U   ATAATATATGTTTCCCAATACTCATATCTATTCTGCTACATTGCCAGCAGCTTTTAGAATGGAACAGGAGAAGCAAAG
PI598465U   ATAATATATGTTTCCCAATACTCATATCTATTCTGCTACATTGCCAGCAGCTTTTAGAATGGAACAGGAGAAGCAAAG
PI406448U   ATAATATATGTTTCCCAATACTCATATCTATTCTGCTACATTGCCAGCAGCTTTTAGAATGGAACAGGAGAAGCAAAG
628702L     ATAATATATGTTTCCCAATACTCATATCTATTCTGCTACATTGCCAGCAGCTTTTAGAATGGAACAGGAGAAGCAAAG
H3169L      ATAATATATGTTTCCCAATACTCATATCTATTCTGCTACATTGCCAGCAGCTTTTAGAATGGAACAGGAGAAGCAAAG
H3526L      ATAATATATGTTTCCCAATACTCATATCTATTCTGCTACATTGCCAGCAGCTTTTAGAATGGAACAGGAGAAGCGAAG
H10391L     ATAATATATGTTTCCCAATACTCATATCTATTCTGCTACATTGCCAGCAGCTTTTAGAATGGAACAGGAGAAGCAAAG
499461L     ATAATATATGTTTCCCAATACTCATATCTATTCTGCTACATTGCCAGCAGCTTTTAGAATGGAACAGGAGCAGCAAAG
H4014       ATAATATATGTTTCCCAATACTCATATCTATTCTGCTACATTGCCAGCAGCTTTTAGAATGGAACAGGAGAAGCAAAG
H5495one    ATAATATATATTTCCCAATACTCATATCCGTTCTGCTACATTGCCAGCAGCTCTTAGAATGGAACAGGAAAAGCAAAG
436946      ATAATATATATTTCCCAATACTCATATCCATTCTGCTACATTGCCAGCAGCTCTTAGAATGGAACAGGAAAAGCAAAG
232258U     ATAATATATATTTCCCAATACTCATATCCATTCTGCTACATTGCCAGCAGCTCTTAGAATGGAACAGGAAAAGCAAAG
531708L     ATAATATATATTTCCCAATACTCATATCCATTCTGCTACATTGCCAGCAGCTCTTAGAATGGAACAGGAAAAGCAAAG
H2148       ATAATATATATTTCCCAATACTCATATCCATTCTGCTACATTGCCAGCAGCTCTTAGAATGGAACAGGAAAAGCAAAG
H1816       ATAATATATATTTCCCAATACTCATATCCAT-CTGCTACATTGCCAGCAGCTCTTAGAATGGAACAGGAAAAGCAAAG
H2024       ATAATATATATTTCCCAATGCTCATATCCATTCTGCTACATTGCCAGCAGCTCTTAGAATGGAACAGGAAAAGCAA--
H1941       ATAATATATATTTCCCAATACTCATATCCATTCTGCTACATTGCCAGCAGCTCTTAGAATGGAACAGGAAAAGCAAAG
331168L     ATAATATATATTTCCCAATACTCATATCCATTCTGCTACATTGCCAGCAGCTCTTAGAATGGAACAGGAGAAGCAAAG
236663L     ATAATATATATTTCCCAATACTCATATCCATTCTGCTACATTGCCAGCAGCTCTTAGAATGGAACAGGAAAAGCAAAG
H10584L     ATAATATATATTTCCCAATACTCATATCCATTCTGCTACACTGCCAGCAGCTCTTAGAATGAAACAGGAAAAGCAAAG
US9601      ATAATATATATTTCCCAATACTCATATCCATTCTGCTACATTGCCAGCAGCTCTTAGAACGGAACAGGAAAAGCAAAG
H9152       ATAATATATATTTCCCAATACTCATATCCATTCTGCTACATTGCCAGCAGCTCTTAGAATGGAACAGGAAAAGCAAAG
PI564933U   ATAATATATGTTTCCCAATACTCATATCTATTCTGCTACATTGCCAGCAGCTTTTAGAATGGAACAGGAGAAGCAAAG
PI229595    ------------TTCTTACGAGCGCATCGA----ACCACATTGCTT-CAGTGTGTAGTA-AGAACATGATACCAATGG
H3526U1     ATAATATATGTTTTTCAATACTCATACCCATTCTGCTACATTGCCAGCAGCTTTTAGCATGAAACAGGAGAAGCAGGG
GQ867861    ATAATATATGTTTTTCAATACTCATACCCATTCTGCTACATTGCCAGCAGCTTTTAGCATGAAACAGGAGAAGCAGCG
GQ867864    ATAATATATGTTTTTCAATACTCATACCCATTCTGCTACATTGCCAGCAGCTTTTAGCATGAA-CAGGAGAAGCAGGG
PI531609L   ATAATATATGTTTTTCAATACTCATACCCATTCTGCTACATTGCCAGCAGCTTTTAGCATGAAACAGGAGAAGCAGGG
H10391U     ATAATATATGTTTTTCAATACTCATACCCATTCTGCTACATTGCCAGCAGCTTTTAGCATGAAACAGGAGAAGCAGGG
PI564932L   ATAATATATGTTTTTCAATACTCATACCCATTCTGCTACATTGCCAGCAGCTTTTAGCATGAAACAGGAGAAGCAGGG
PI345585U   ATAATATATGTTTTTCAATACTCATACCCATTCTGCTACATTGCCAGCAGCTTTTAGCATGAAACAGGAGAAGCAGGG
PI406467U   ATAATATATGTTTTTCAATACTCATACCCATTCTGCTACATTGCCAGCAGCTTTTAGCATGAAACAGGAGAAGCAGGG
PI439999U   ATAATATATGTTTTTCAATACTCATACCCATTCTGCTACATTGCCAGCAGCTTTTAGCATGAAACAGGAGAAGCAGGG
PI564930L   ATAATATATGTTTTTCAATACTCATACCCATTCTGCTACATTGCCAGCAGCTTTTAGCATGAAACAGGAGAAGCAGGG
PI406448L   ATAATATATGTTTTTCAATACTCATACCCATTCTGCTACATTGCCAGCAGCTTTTAGCATGAAACAGGAGAAGCAGGG
PI598465L   ATAATATATGTTTTTCAATACTCATACCCATTCTGCTACATTGCCAGCAGCTTTTAGCATGAAACAGGAGAAGCAGGG
EU187442    ATAATATATGTTTTTCAATACTCATACCCATTCTGCTACATTGCCAGCAGCTTTTAGCATGAAACAGGAGAAGCAGGG
628702U     ATAATATATGTTTTTCAATACTCATACCCATTCTGCTACATTGCCAGCAGCTTTTAGCATGAAACAGGAGAAGCAGGG
H10584U     ATAATATATGTTTTTCAATACTCATACCCATTCTGCTACATTGCCAGCAGCTTTTAGCATGAAACAGGAGAAGCAGGG
531708U     ATAATATATGTTTTTCAATACTCATACCCATTCTGCTACATTGCCAGCAGCTTTTAGCATGAAACAGGAGAAGCAGGG
499461u1    ATAATATATGTTTTTCAATACTCATACCCATTCTGCTACATTGCCAGCAGCTTTTAGCATGAAACAGGAGAAGCAGGG
PI228390    ATAATATATATTTTTCAATACTCATATCCATTCTGCTACATTGCCAGCAGCTTTTAGCATGAAACAGGAGAAGCAGGG
PI401326    ATAATATATATTTTTCAATACTCATATCCATTCTGCTACATTGCCAGCAACTTTTAGCATGAAACAGGAGAAGCAGGG
PI420842    ATAATATATGTTTTTCAATACTCATACCCATTCTGCTACATTGCCAGCAGCTTTTAGCATGAAACAGGAGAAGCAGGG
PI537379    ATAATATATGTTTTTCAATACTCATACCCATTCTGCTACATTGCCAGCAGCTTTTAGCATGAAACAGGAGAAGCAGGG
PI516184    ATAATATATGTTTTTCAATACTCATACCCATTCTGCTACATTGCCAGCAGCTTTTAGCATGAAACAGGAGAAGCAGGG
PI531752    ATAATATATGTTTTTCAATACTCATACCCATTCTGCTACATTGCCAGCAGCTTTTAGCATGAAACAGGAGAAGCAGGG


E142012     TACAAACCTGTCTTCTTAAACGACGAAGGGTCTTCACCAATAGGTCAGGATTCCTATGAATTCCAACCCAAC-AACCA
E531712     TACAAACCTGTCTTCTTAAACGACGAAGGGTCTTCACCAATAGGTCAGGATTCCTATGAATTCCAACCCAAC-AACCA
W533014     TACAAACCTGTCTTCTTAAACGACGAAGGGTCTTCACCAATAGGTCAGGATTCCTATGAATTCCAACCCAAC-AACCA
W547363     TACAAACCTGTCTTCTTAAACGACGAAGGGTCTTCACCAATAGGTCAGGATTCCTATGAATTCCAACCCAAC-AACCA
P383534     TACAAACCTGTCTTCTTAAACGACGAAGGGTCTTCACCAATAGGTCAGGATTCCTATGAATTCCAACCCAAC-AACCA
PI531609U   CACAAACCTGTCTTCTTAAACGACGAAGTGTCCTCACCAATAGGTCAGGATTCCTATGAATTCCAACCCAAC-AACCA
```

```
PI406467L   CACAAACCTGTCTTCTTAAACGACGAAGTGTCTTCACCAATAGGTCAGGATTCCTATGAATTCCAACCCAAC-AACCA
H10339      CACAAACCTGTCTTCTTAAACGACGAAGTGTCTTCACCAATAGGTCAGGATTCCTATGAATTCCAACCCAAC-AACCA
PI439999L   CACAAACCTGTCTTCTTAAACGACGAAGTGTCTTCACCAATAGGTCAGGATTCCTATGAATTCCAACCCAAC-AACCA
PI564932U   CACAAACCTGTCTTCTTAAACGACGAAGTGTCTTCACCAATAGGTCAGGATTCCTATGAATTCCAACCCAAC-AACCA
PI564933L   CACAAACCTGTCTTCTTAAACGACGAAGTGTCTTCACCAATAGGTCAGGATTCCTATGAATTCCAACCCAAC-AACCA
PI564930U   CACAAACCTGTCTTCTTAAACGACGAAGTGTCTTCACCAATAGGTCAGGATTCCTATGAATTCCAACCCAAC-AACCA
PI598465U   CACAAACCTGTCTTCTTAAACGACGAAGTGTCTTCACCAATAGGTCAGGATTCCTATGAATTCCAACCCAAC-AACCA
PI406448U   CACAAACCTGTCTTCTTAAACGACGAAGTGTCTTCACCAATAGGTCAGGATTCCTATGAATTCCAACCCAAC-AACCA
628702L     CACAAACCTGTCTTCTTAAACGACGAAGTGTCTTCACCAATAGGTCAGGATTCCTATGAATTCCAACCCAAC-AACCA
H3169L      CACAAACCTGTCTTCTTAAACGACGAAGTGTCTTCACCAATAGGTCAGGATTCCTATGAATTCCAACCCAAC-AACCA
H3526L      CACAAACCTGTCTTCTTAAACGACGAAGTGTCTTCACCAATAGGTCAGGATTCCTATGAATTCCAACCCAAC-AACCA
H10391L     CACAAACCTGTCTTCTTAAACGACGAAGTGTCTTCACCAATAGGTCAGGATTCCTATGAATTCCAACCCAAC-AACCA
499461L     CACAAACCTGTCTTCTTAAACGACGAAGTGTCTTCACCAATAGGTCAGGATTCCTATGAATTCCAACCCAAC-AACCA
H4014       CACAAACCTGTCTTCTTAAACGACGAAGTGTCTTCACCAATAGGTCAGGATTCCTATGAATTCCAACCCAAC-AACCA
H5495one    CACAAACCTGTCTTCTTAAACGACGAAGTGTCTTCACCAATAGGTCAGGATTCCTATGAATTCCAACCCAAC-AACCA
436946      CACAAACCTGTCTTCTTAAACGACGAAGTGTCTTCACCAATAGGTCAGGATTCCTATGAATTCCAACCCAAC-AACCA
232258U     CACAAACCTGTCTTCTTAAACGACGAAGTGTCTTCACCAATAGGTCAGGATTCCTATGAATTCCAACCCAAC-AACCA
531708L     CACAAACCTGTCTTCTTAAACGACGAAGTGTCTTCACCAATAGGTCAGGATTCCTATGAATTCCAACCCAACTAACCA
H2148       CACAAACCTGTCTTCTTAAACGACGAAGTGTCTTCACCAATAGGTCAGGATTCCTATGAATTCCAACCCAAC-AACCA
H1816       CACAAACCTGTCTTCTTAAACGACGAAGTGTCTTCACCAATAGGTCAGGATTCCTGTGAATTCCAACCCAAC-AACCA
H2024       CACAAACCTGTCTTCTTAACCGACGAAGTGTCTTCACCAATAGGTCAGGATTCCTATGAATTCCAACCCAAC-AACCA
H1941       CACAAACCTGTCTTCTTAAACGACGAAGTGTCTTCACCAATAGGTCAGGATTCCTATGAATTCCAACCCAAC-AACCA
331168L     CACAAACCTGTCTTCTTAAACGACGAAGTGTCTTCACCAATAGGTCAGGATTCCTATGAATTCCAACCCAAC-AACCA
236663L     CACAAACCTGTCTTCTTAAACGACGAAGTGTCTTCACCAATAGGTCAGGATTCCTATGAATTCCAACCCAAC-AACCA
H10584L     CACAAACCTGTCTTCTTAAACGACGAAGTGTCTTCACCAATAGGTCAGGATTCCTATGAATTCCAAACCAAC-AACCA
US9601      CACAAACCTGTCTTCTTAAACGACGAAGTGTCTTCACCAATAGGTCAGGATTCCTATGAATTCCAACCCAAC-AACCA
H9152       CACAAACCTGTCTTCTTAAACGACGAAGTGTCTTCACCAATAGGTCAGGATTCCTATGAATTCCAACCCAAC-AACCA
PI564933U   CACAAACCTGTCTTCTTAAACGACGAAGTGTCTTCACCAATAGGTCAGGATTCCTATGAATTCCAACCCAAC-AACCA
PI229595    TAAATACCTGTCTTCGTAAACGACGAGGTGTCTTCACCAATGTTGAAGGATTCCTATGAATTCCCACCCCAC-TTCCA
H3526U1     TACAAACCTGTCTTCTTAAACGACGAAGTGTCTTCACTAATAGGTCAGGATTCCTATGAATTCCAACCCAAC-AACCA
GQ867861    TACAAACCTGTCTTCTTAAACGACGAAGTGTCTTCACTAATAGGTCAGGATTCCTATGAATTCCAACCCAAC-AACCA
GQ867864    TACAAACCTGTCTTCTTAAACGACGAAGTGTCTTCACTAATAGGTCAGGATTCCTATGAATTCCAACCCAAC-AACCA
PI531609L   TACAAACCTGTCTTCTTAAACGACGAAGTGTCTTCACTAATAGGTCAGGATTCCTATGAATTCCAACCCAAC-AACCA
H10391U     TACAAACCTGTCTTCTTAAACGACGAAGTGTCTTCACTAATAGGTCAGGATTCCTATGAATTCCAACCCAAC-AACCA
PI564932L   TACAAACCTGTCTTCTTAAACGACGAAGTGTCTTCACTAATAGGTCAGGATTCCTATGAATTCCAACCCAAC-AACCA
PI345585U   TACAAACCTGTCTTCTTAAACGACGAAGTGTCTTCACTAATAGGTCAGGATTCCTATGAATTCCAACCCAAC-AACCA
PI406467U   TACAAACCTGTCTTCTTAAACGACGAAGTGTCTTCACTAATAGGTCAGGATTCCTATGAATTCCAACCCAAC-AACCA
PI439999U   TACAAACCTGTCTTCTTAAACGACGAAGTGTCTTCACTAATAGGTCAGGATTCCTATGAATTCCAACCCAAC-AACCA
PI564930L   TACAAACCTGTCTTCTTAAACGACGAAGTGTCTTCACTAATAGGTCAGGATTCCTATGAATTCCAACCCAAC-AACCA
PI406448L   TACAAACCTGTCTTCTTAAACGACGAAGTGTCTTCACTAATAGGTCAGGATTCCTATGAATTCCAACCCAAC-AACCA
PI598465L   TACAAACCTGTCTTCTTAAACGACGAAGTGTCTTCACTAATAGGTCAGGATTCCTATGAATTCCAACCCAAC-AACCA
EU187442    TACAAACCTGTCTTCTTAAACGACGAAGTGTCTTCACTAATAGGTCAGGATTCCTATGAATTCCAACCCAAC-AACCA
628702U     TACAAACCTGTCTTCTTAAACGACGAAGTGTCTTCACTAATAGGTCAGGATTCCTATGAATTCCAACCCAAC-AACCA
H10584U     TACAAACCTGTCTTCTTAAACGACGAAGTGTCTTCACTAATAGGTCAGGATTCCTATGAATTCCAACCCAAC-AACCA
531708U     TACAAACCTGTCTTCTTAAACGACGAAGTGTCTTCACTAATAGGTCAGGATTCCTATGAATTCCAACCCAAC-AACCA
499461u1    TACAAACCTGTCTTCTTAAACGACGAAGTGTCTTCACTAATAGGTCAGGATTCCTATGAATTCCAACCCAAC-AACCA
PI228390    TACAAACCTGTCTTCTTAAACGGCGAAGTGTCTTCACCAATAGGTCAGGATTCCTATGAATTCCAACCCAAC-AACCA
PI401326    TACAAACCTGTCTTCTTAAACGGCGAAGTGTCTTCACCAATAGGTCAGGATTCCTATGAATTCCAACCCAAC-AACCA
PI420842    TACAAACCTGTCTTCTTAAACGACGAAGTGTCTTCACTAATAGGTCAGGATTCCTATGAATTCCAACCCAAC-AACCA
PI537379    TACAAACCTGTCTTCTTAAACGACGAAGTGTCTTCACTAATAGGTCAGGATTCCTATGAATTCCAACCCAAC-AACCA
PI516184    TACAAACCTGTCTTCTTAAACGACGAAGTGTCTTCACTAATAGGTCAGGATTCCTATGAATTCCAACCCAAC-AACCA
PI531752    TACAAACCTGTCTTCTTAAACGACGAAGTGTCTTCACTAATAGGTCAGGATTCCTATGAATTCCAACCCAAC-AACCA
```

```
E142012     TTGACAAATATTTT-AGCAGCTTGAGGAATGACTGCCGGTGATATCTCCTGTTAAAAAGGAATCAAGAACGAAATCAG
E531712     TTGACAAATATTTT-AGCAGCTTGAGGAATGACTGCCGGTGATATCTCCTGTTAAAAAAGAATCAAGAACGAAATCAG
W533014     TTGACAAATATTTT-AGCAGCTTGAGGAATGACTGCCGGTGATATCTCCTGTTAAAA-CGAATCCAGAACGAAATCAG
W547363     TTGACAAATATTTT-AGCAGCTTGAGGAATGACTGCCGGTGATATCTCCTGTTAAAAACGAATCCAGAACGAAATCAG
P383534     TTGACAAATATTTT-AGCAGCTTGAGGAATGACTGCCGGTGATATCTCCTGTTAAAAACGAATCCAGAACGAAATCAG
PI531609U   TTGACAAATATTTT-AGCAGCTTGAGGAATGACTGCCGGTGATATCTCCTGTTAAAGACGATTCCAGAACGAAATCAG
PI406467L   TTGACAAATATTTT-AGCAGCTTGAGGAATGACTGCCGGTGATATCTCCTGTTAAAGACGATTCCAGAACGAAATCAG
H10339      TTGACAAATATTTT-AGCAGCTTGAGGAATGACTGCCGGTGATATCTCCTGTTAAAGACGATTCCAGAACGAAATCAG
```

```
PI439999L   TTGACAAATATTTT-AGCAGCTTGAGGAATGACTGCCGGTGATATCTCCTGTTAAAGACGATTCCAGAACGAAATCAG
PI564932U   TTGACAAATATTTT-AGCAGCTTGAGGAATGACTGCCGGTGATATCTCCTGTTAAAGACGATTCCAGAACGAAATCAG
PI564933L   TTGACAAATATTTT-AGCAGCTTGAGGAATGACTGCCGGTGATATCTCCTGTTAAAGACGATTCCAGAACGAAATCAG
PI564930U   TTGACAAATATTTT-AGCAGCTTGAGGAATGACTGCCGGTGATATCTCCTGTTAAAGACGATTCCAGAACGAAATCAG
PI598465U   TTGACAAATATTTT-AGCAGCTTGAGGAATGACTGCCGGTGATATCTCCTGTTAAAGACGATTCCAGAACGAAATCAG
PI406448U   TTGACAAATATTTT-AGCAGCTTGAGGAATGACTGCCGGTGATATCTCCTGTTAAAGACGAATCCAGAACGAAATCAG
628702L     TTGACAAATATTTT-AGCAGCTTGAGGAATGACTGCCGGTGATATCTCCTGTTAAAGACGATTCCAGAACGAAATCAG
H3169L      TTGACAAATATTTT-AGCAGCTTGAGGAATGACTGCCGGTGATATCTCCTGTTAAAGACGATTCCAGAACGAAATCAG
H3526L      TTGACAAATATTTT-AGCAGCTTGAGGAATGACTGCCGGTGATATCTCCTGTTAAAGACAAATCCAGAACGAAATCAG
H10391L     TTGACAAATATTTT-AGCAGCTTGAGGAATGACTGCCGGTGATATCTCCTGTTAAAGACAAATCCAGAACGAAATCAG
499461L     TTGACAAATATTTT-AGCAGCTTGAGGAATGACTGCCGGTGATATCTCCTGTTAAAGACAAATCCAGAACGAAATCAG
H4014       TTGACAAATATTTT-AGCAGCTTGAGGAATGACTGCCGGTGATATCTCCTGTTAAAGACAAATCCAGAACGAAATCAG
H5495one    TTGACAAATATTTTTAGCAGCTTGAGGAATGACTGCCGGTGATATCTCCTATTAAAGATGAATCCAGAACGAAATCAG
436946      TTGACAAATATTTT-AGCAGCTTGAGGAATGACTGCCGGTGATATCTCCTATTAAAGATGAATCCAGAACGAAATCAG
232258U     TTGACAAATATTT--AGCAGCTTGAGGAATGACTGCCGGTGATATCTCCTATTAAAGATGAATCCAGAACGAAATCAG
531708L     TTGACAAATATTTT-AGCAGCTTGAGGAATGACTGCCGGTGATATCTCCTATTAAAGATGAATCCAGAACGAAATCAG
H2148       TTGACAAATATTTT-AGCAGCTTGAGGAATGACTGCCGGTGATATCTCCTATTAAAGATGAATCCAGAACGAAATCAG
H1816       TTGACAAATATTTT-AGCAGCTTGAGGAATGACTGCCGGTGATATCTCCTATTAAAGATGAATCCAGAACGAAATCAG
H2024       TTGACAAATATTTT-AGCAGCTTGAGGAATGACTGCCGGTGATATCTCCTATTAAAGATGAATCCAGAACGAAATCAG
H1941       TTGACAAATATTTT-AGCAGCTTGAGGAATGACTGCCGGTGATATCTCCTATTAAAGATGAATCCAGAACGAAATCAG
331168L     TTGACAAATATTTT-AGCAGCTTGAGGAATGACTGCCGGTGATATCTCCTATAAAAGATGAATCCAGAACGAAATCAG
236663L     TTGACAAATATTTT-AGCAGCTTGAGGAATGACTGCCGGTGATATCTCCTATTAAAGACGAATCCAGAACGAAATCAG
H10584L     TTGACAAATATTTT-AGCAGCTTGAGGAATGACTGCCGGTGATATCTCCTATTAAAGACGAATCCAGAACGAAATCAG
US9601      TTGACAAATATTTT-AGCAGCTTGAGGAATGACTGCCGGTGATATCTCCTATTAAAGACGAATCCAGAACGAAATCAG
H9152       TTGACAAATATTTT-AGCAGCTTGAGGAATGACTGCCGGTGATATCTCCTATTAAAGACGAATCCAGAACGAAATCAG
PI564933U   TTGACAAATATTTT-AGCAGCTTGAGGAATGACTGCCGGTGATATCTCCTGTTAAAGACGATTCCAGAACGAAATCAG
PI229595    TTGACTAATATTTT-AGCAGCTTGAGGAATGACTGCCGGTGATATCTCCTGTTAAAAACAAATCCAGAACCAAATCAG
H3526U1     TTGACAAATATTTT-AGCAGCTTGAGGAATGACTGCCGGTGATATCTCCTGTTAAAAACGAATCCAGAACAAAATCAG
GQ867861    TTGACAAATATTTT-AGCAGCTTGAGGAATGACTGCCGGTGATATCTCCTGTTAAAAACGAATCCAGAACAAAATCAG
GQ867864    TTGACAAATATTTT-AGCAGCTTGAGGAATGACTGCCGGTGATATCTCCTGTTAAAAACGAATCCAGAACAAAATCAG
PI531609L   TTGACAAAAATTTT-AGCAGCTTGAGGAATGACTGCCGGTGATATCTCCTGTTAAAAACGAATCCAGAACAAAATCAG
H10391U     TTGACAAATATTTT-AGCAGCTTGAGGAATGACTGCCGGTGATATCTCCTGTTAAAAACGAATCCAGAACAAAATCAG
PI564932L   TTGACAAAAATTTT-AGCAGCTTGAGGAATGACTGCCGGTGATATCTCCTGTTAAAAACGAATCCAGAACAAAATCAG
PI345585U   TTGACAAAAATTTT-AGCAGCTTGAGGAATGACTGCCGGTGATATCTCCTGTTAAAAACGAATCCAGAACAAAATCAG
PI406467U   TTGACAAAAATTTT-AGCAGCTTGAGGAATGACTGCCGGTGATATCTCCTGTTAAAAACGAATCCAGAACAAAATCAG
PI439999U   TTGACAAAAATTTT-AGCAGCTTGAGGAATGACTGCCGGTGATATCTCCTGTTAAAAACGAATCCAGAACAAAATCAG
PI564930L   TTGACAAAAATTTT-AGCAGCTTGAGGAATGACTGCCGGTGATATCTCCTGTTAAAAACGAATCCAGAACAAAATCAG
PI406448L   TTGACAAAAATTTT-AGCAGCTTGAGGAATGACTGCCGGTGATATCTCCTGTTAAAAACGAATCCAGAACAAAATCAG
PI598465L   TTGACAAAAATTTT-AGCAGCTTGAGGAATGACTGCCGGTGATATCTCCTGTTAAAAACGAATCCAGAACAAAATCAG
EU187442    TTGACAAAAATTTT-AGCAGCTTGAGGAATGACAGCCGGTGATATCTCCTGTTAAAAACGAATCCAGAACAAAATCAG
628702U     TTGACAAAAATTTT-AGCAGCTTGAGGAATGACTGCCGGTGATATCTCCTGTTAAAAACGAATCCAGAACAAAATCAG
H10584U     TTGACAAATATTTT-AGCAGCTTGAGGAATGACTGCCGGTGATATCTCCTGTTAAAAACGAATCCAGAACAAAATCAG
531708U     TTGACAAATATTTT-AGCAGCTTGAGGAATGACTGCCGGTGATATCTTCTGTTAAAAATGAATCCAGAACAAAATCAG
499461u1    TTGACAAATATTTT-AGCAGCTTGAGGAATGACTGCCGGTGATATCTCCTGTTAAAAACGAATCCAGAACAAAATCAG
PI228390    TTGACAAAAATTTT-AGCAGCTTGAGGAATGACTGCCGGTGATATCTCCTGTTAAAATCGAATCCAGAACAAAATCAG
PI401326    TTGACAAAAATTTT-AGCAGCTTGAGGAATGACTGCCGGTGATATCTCCTGTTAAAAACGAATCCAGAACAAAATCAG
PI420842    TTGACAAAAATTTT-AGCAGCTTGAGGAATGACTGCCGGTGATATCTCCTGTTAAAAACGAATCCAGAACAAAATCAG
PI537379    TTGACAAAAATTTT-AGCAGCTTGAGGAATGACTGCCGGTGATATCTCCTGTTAAAAACGAATCCAGAACAAAATCAG
PI516184    TTGACAAAAATTTT-AGCAGCTTGAGGAATGACTGCCGGTGATATCTCCTGTTAAAAACGAATCCAGAACAAAATCAG
PI531752    TTGACAAAAATTTT-AGCAGCTTGAGGAATGACTGCCGGTGATATCTCCTGTTAAAAACGAATCCAGAACAAAATCAG


E142012     TGACTGTTGTAACTGGTAAAT-GACACGAAACTTTAACCTTGGGTAAGATAAGCAAATGAAAATGCATGCTCACACAT
E531712     TGACTGTTGTAACTGGTAAAT-GACACGAAAGTTTAACCTTGGGTAAGATAAGCAAATGAAAATGCATGCACACACAT
W533014     TGACTGTTGTAACTGGTAAATTGACAGGAAACTTAACCTT-GGGTAAGATAAGCAAATGAGAATGCATGCACACACAT
W547363     TGACTGTTGTAACTGGTAAATTGACAGGAAACTTAACCTT-GGGTAAGATAAGCAAATGAGAATGCATGCACACACAT
P383534     TGACTGTTGTAACTGGTAAATTGACACGAAACTTAACCTTTGGATAAGATAAGCAAATGAAAATGCATGCGCACACAT
PI531609U   TGACTGTTGTAACTGGTAAAT-GACACGAAACTTTAACCTTGGGTAAGATAAGCAAATGAAAATGCATGCACGCACAT
PI406467L   TGACTGTTGTAACTGGTAAAT-GACACGAAACTTTAACCTTGGGTAAGATAAGCAAATGAAAATGCATGCACGCACAT
H10339      TGACTGTTGTAACTGGTAAAT-GACACGAAACTTTAACCTTGGGTAAGATAAGCAAATGAAAATGCATGCACGCACAT
PI439999L   TGACTGTTGTAACTGGTAAAT-GACACGAAACTTTAACCTTGGGTAAGATAAGCAAATGAAAATGCATGCACGCACAT
PI564932U   TGACTGTTGTAACTGGTAAAT-GACACGAAACTTTAACCTTGGGTAAGATAAGCAAATGAAAATGCATGCACGCACAT
```

PI564933L	TGACTGTTGTAACTGGTAAAT-GACACGAAACTTTAACCTTGGGTAAGATAAGCAAATGAAAATGCATGCACGCACAT
PI564930U	TGACTGTTGTAACTGGTAAAT-GACACGAAACTTTAACCTTGGGTAAGATAAGCAAATGAAAATGCATGCACGCACAT
PI598465U	TGACTGTTGTAACTGGTAAAT-GACACGAAACTTTAACCTTGGGTAAGATAAGCAAATGAAAATGCATGCACGCACAT
PI406448U	TGACTGTTGTAACTGGTAAAT-GACACGAAACTTTAACCTTGGGTAAGATAAGCAAATGAAAATGCATGCACGCACAT
628702L	TGACTGTTGTAACTGGTAAAT-GACACGAAACTTTAACCTTGGGTAAGATAAGCAAATGAAAATGCATGCACGCACAT
H3169L	TGACTGTTGTAACTGGTAAAT-GACACGAAACTTTAACCTTGGGTAAGATAAGCAAATGAAAATGCATGCACGCACAT
H3526L	TGACTGTTGTAACTGGTAAAT-GACACGAAACTTTAACCTTGGGTAAGATAAGCAAATGAAAATGCATGCACGCACAT
H10391L	TGACTGTTGTAACTGGTAAAT-GACACGAAACTTTAACCTTGGGTAAGATAAGCAAATGAAAATGCATGCACGCACAT
499461L	TGACTGTTGTAACTGGTAAAT-GACACGAAACTTTAACCTTGGGTAAGATAAGCAAATGAAAATGCATGCACGCACAT
H4014	TGACTGTTGTAACTGGTAAAT-GACACGAAACTTTAACCTTGGGTAAGATAAGCAAATGAAAATGCATGCACGCACAT
H5495one	TGACTGTTGTAACTGGTAAAT-GACACGAAACTTTAACCTTGGGTAAGATAAGCAAATGAAAATGCATGCACGCACAT
436946	TGACTGTTGTAACTGGTAAAT-GACACGAAACTTTAACCTTGGGTAAGATAAGCAAATGAAAATGCATGCACGCACAT
232258U	TGACTGTTGTAACTGGTAAAT-GACACGAAACTTTAACCTTGGGTAAGATAAGCAAATGAAAATG-ATGCACGCACAT
531708L	TGACTGTTGTAACTGGTAAAT-GACACGAAACTTTAACCTTGGGTAAGATAAGCAAATGAAAATGCATGCACGCACAT
H2148	TGACTGTTGTAACTGGTAAAT-GACACGAAACTTTAACCTTGGGTAAGATAAGCAAATGAAAATGCATGCACGCACAT
H1816	TGACTGTTGTAACTGGTAAAT-GACACAAAACTTTAACCTTGGGTAAGATAAGCAAATGAAAATGCATGCACGCACAT
H2024	TGACTGTTGTAACTGGTAAAT-GACACGAAACTTTAACCTTGGGTAAGATAAGCAAATGAAAATGCATGCACGCACAT
H1941	TGACTGTTGTAACTGGTAAAT-GACACGAAAATTGAACCTTGGGTAAGATAAGCAAATGAAAATGCATGCACGCACAT
331168L	TGACTGTTGTAACTGGTAAAT-GACACGAAACTTTAACCTTGGGTAAGATAAGCAAATGAAAATGCATGCACGCACAT
236663L	TGACTGTTGTAACTGGTAAAT-GACACGAAACTTTAACCTTGGGTAAGATAAGCAAATGAAAATGCATGCACACACAT
H10584L	TGACTGTTGTAACTGGTAAAT-GACACGAAACTTTAACCTTGGGTAAGATAAGCAAATGAAAATGCATGCACGCACAT
US9601	TGACTGTTGTAACTGGTAAAT-GACACGAAACTTTAACCTTGGGTAAGATAAGCAAATGAAAATGCATCTACGCACAT
H9152	TGACTGTTGTAACTGGTAAAT-GACACGAAACTTTAACCTTGGGTAAGATAAGCAAATGAAAATGCATGCACGCACAT
PI564933U	TGACTGTTGTAACTGGTAAAT-GACACGAAACTTTAACCTTGGGTAAGATAAGCAAATGAAAATGCATGCACGCACAT
PI229595	TGACTGTTGTAAC-------------------------CTTGGGTAAAATGAGCAAATGAAAATGCATGCA-------
H3526U1	TGACTGTTGTAACTGGTAAAT-GACACGAAACTTTAAACTTGGGTAAGATAAGCAAATGAAAATGCATGCGCACACAT
GQ867861	TGACTGTTGTAACTGGTAAAT-GACACGAAACTTTAAACTTGGGTAAGATAAGCAAATGAAAATGCATGCGCACACAT
GQ867864	TGACTGTTGTAACTGGTAAAT-GACACGAAACTTTAAACTTGGGTAAGATAAGCAAATGAAAATGCATGCGCACACAT
PI531609L	TGACTGTTGTAACTGGTAAAT-GACACGAAACTTTAAACTTGGGTAAGATAAGCAAATGAAAATGCATGCGCACACAT
H10391U	TGACTGTTGTAACTGGTAAAT-GACACGAAACTTTAAACTTGGGTAAGATAAGCAAATGAAAATGCATGCGCACACAT
PI564932L	TGACTGTTGTAACTGGTAAAT-GACACGAAACTTTAAACTTGGGTAAGATAAGCAAATGAAAATGCATGCGCACACAT
PI345585U	TGACTGTTGTAACTGGTAAAT-GACACGAAACTTTAAACTTGGGTAAGATAAGCAAATGAAAATGCATGCGCACACAT
PI406467U	TGACTGTTGTAACTGGTAAAT-GACACGAAACTTTAAACTTGGGTAAGATAAGCAAATGAAAATGCATGCGCACACAT
PI439999U	TGACTGTTGTAACTGGTAAAT-GACACGAAACTTTAAACTTGGGTAAGATAAGCAAATGAAAATGCATGCGCACACAT
PI564930L	TGACTGTTGTAACTGGTAAAT-GACACGAAACTTTAAACTTGGGTAAGATAAGCAAATGAAAATGCATGCGCACACAT
PI406448L	TGACTGTTGTAACTGGTAAAT-GACACGAAACTTTAAACTTGGGTAAGATAAGCAAATGAAAATGCATGCGCACACAT
PI598465L	TGACTGTTGTAACTGGTAAAT-GACACGAAACTTTAAACTTGGGTAAGATAAGCAAATGAAAATGCATGCGCACACAT
EU187442	TGACTGTTGTAACTGGTAAAT-GACACGAAACTTTAAACTTGGGTAAGATAAGCAAATGAAAATGCATACGCACACAT
628702U	TGACTGTTGTAACTGGTAAAT-GACACGAAACTTTAAACTTGGGTAAGATAAGCAAATGAAAATGCATGCGCACACAT
H10584U	TGACTGTTGTAACTGGTAAAT-GACACGAAACTTTAAACTTGGGTAAGATAAGCAAATGAAAATGCATGCGCACACAT
531708U	TGACTGTTGTAACTGGTAAAT-GACACGAAACTTTAAACTTGGGTAAGATAAGCAAATGAAAATGCATGCGCACACAT
499461u1	TGACTGTTGTAACTGGTAAAT-GACACGAAACTTTAAACTTGGGTAAGATAAGCAAATGAAAATGCATGCGCACACAT
PI228390	TGACTGTTGTAACTGGTAAAT-AACACGAAACTTTAACCTTGGGTAAGGTAAGCAAATGAAAATGCATGCGCACACAT
PI401326	TGACTGTTGTAACTGGTAAAT-AACACGAAACTTTAACCTTGGGTAAGGTAAGCAAATGAAAATGCATGCGCACACAT
PI420842	TGACTGTTGTAACTGGTAAAT-GACACGAAACTTTAAACTTGGGTAAGATAAGCAAATGAAAATGCATGCGCACACAT
PI537379	TGACTGTTGTAACTGGTAAAT-GACACGAAACTTTAAACTTGGGTAAGATAAGCAAATGAAAATGCATGCGCACACAT
PI516184	TGACTGTTGTAACTGGTAAAT-GACACGAAACTTTAAACTTGGGTAAGATAAGCAAATGAAAATGCATGCGCACACAT
PI531752	TGACTGTTGTAACTGGTAAAT-GACACGAAACTTTAAACTTGGGTAAGATAAGCAAATGAAAATGCATGCGCACACAT

E142012	TGAGAAGGAG---AAACAAACCTCAAAATTTTCTGTGCCCCACTCTTCCAAAAATTCCAAAATAGGATTTGCGGCAGA
E531712	TGAGAAGGGG---AAACAAACCTCAAAATTTTCTGTGCCCCACTCTTCCAAAAATTCCAAAATAGGATTTGCGGCAGA
W533014	TGAGAAAGGG---AAACAAACCTCAAAATTTTCTGTGCCCCACTCTTCCAAAAATTCCAAAATAGGATTTGCGGCAGA
W547363	TGAGAAAGGG---AAACAAACCTCAAAATTTTCTGTGCCCCACTCTTCCAAAAATTCCAAAATAGGATTTGCGGCAGA
P383534	TGAGAAAGGG---AAACAAACCTCAAAATTTTCTGTGCCCCACTCTTCCAAAAATTCCAAAATAGGATTTGCGGCAGA
PI531609U	TGAGAAGGGGCTAAAACAAACCTCGAAATTTTCTGTGCCCCATTCTTCCAAAAATTCCAAAATAGGATTTGCAGCAGA
PI406467L	TGAGAAGGGGCTAAAACAAACCTCGAAATTTTCTGTGCCCCATTCTTCCAAAAATTCCAAAATAGGATTTGCAGCAGA
H10339	TGAGAAGGGGCTAAAACAAACCTCGAAATTTTCTGTGCCCCATTCTTCCAAAAATTCCAAAATAGGATTTGCAGCAGA
PI439999L	TGAGAAGGGGCTAAAACAAACCTCGAAATTTTCTGTGCCCCATTCTTCCAAAAATTCCAAAATAGGATTTGCAGCAGA
PI564932U	TGAGAAGGGGCTAAAACAAACCTCGAAATTTTCTGTGCCCCATTCTTCCAAAAATTCCAAAATAGGATTTGCAGCAGA
PI564933L	TGAGAAGGGGCTAAAACAAACCTCGAAATTTTCTGTGCCCCATTCTTCCAAAAATTCCAAAATAGGATTTGCAGCAGA
PI564930U	TGAGAAGGGGCTAAAACAAACCTCGAAATTTTCTGTGCCCCATTCTTCCAAAAATTCCAAAATAGGATTTGCAGCAGA

```
PI598465U   TGAGAAGGGGCTAAAACAAACCTCGAAATTTTCTGTGCCCCATTCTTCCAAAAATTCCAAAATAGGATTTGCAGCAGA
PI406448U   TGAGAAGGG-CTAAAACAAACCTCGAAATTTTCTGTGCCCCATTCTTCCAAAAATTCCAAAATAGGATTTGCAGCAGA
628702L     TGAGAAGGGGCTAAAACAAACCTCGAAATTTTCTGTGCCCCATTCTTCCAAAAATTCCAAAATAGGATTTGCAGCAGA
H3169L      TGAGAAGGGGCTAAAACAAACCTCGAAATTTTCTGTGCCCCATTCTTCCAAAAATTCCAAAATAGGATTTGCAGCAGA
H3526L      GGAGAAGGAGCTAAAACAAACCTCGAAATTTTCTGTGCCCCATTCTTCCAAAAATTCCAAAATAGGATTTGCAGCAGA
H10391L     TGAGAAGGGGCTAAAACAAACCTCGAAATTTTCTGTGCCCCATTCTTCCAAAAATTCCAAAATAGGATTTGCAGCAGA
499461L     TGAGAAGGGGCTAAAACAAACCTCGAAATTTTCTGTGCCCCATTCTCCCAAAAATTCCAAAATAGGATTTGCAGCAGA
H4014       TGAGAAGGGGCTAAAACAAACCTCGAAATTTTCTGTGCCCCATTCTTCCAAAAATTCCAAAATAGGATTTGCAGCAGA
H5495one    TGAGAAGGGGCTAAAACAAACCTCGAAATTTTCTGTGCCCCATTCTTCCAAAAATTCCAAAATAGGATTTGCAGCAGA
436946      TGAGAAGGGGCTAAAACAAACCTCGAAATTTTCTGTGCCCCATTCTTCCAAAAATTCCAAAATAGGATTTGCAGCAGA
232258U     TGAGAAGGGGCTAAAACAAACCTCGAAATTTTCTGTGCCCCATTCTTCCAAAAATTCCAAAATAGGATTTGCAGCAGA
531708L     TGAGAAGGGGCTAAAACAAACCTCGAAATTTTCTGTGCCCCATTCTTCCAAAAATTCCAAAATAGGATTTGCAGCAGA
H2148       TGAGAAGGGGCTAAAACAAACCTCGAAATTTTCTGTGCCCCATTCTTCCAAAAATTCCAAAATAGGATTTGCAGCAGA
H1816       TGAGAAGGGGCTAAAACAAACCTCGAAATTTTCTGTGCCCCATTCTTCCAAAAATTCCAAAATAGGATTTGCAGCAGA
H2024       TGAGAAGGGGCTAAAACAAACCTCAAAATTTTCCGTGCCCCATTCTTCCAGAAATTCCAAAATAGGATTTGCAGCAGA
H1941       TGAGAAGGGGCTAAAACAAACCTCGAAATTTTCTGTGCCCCATTCTTCCAAAAATTCCAAAATAGGATTTGCAGCAGA
331168L     AGAGAAGGGGCTAAAACAAACCTCGAAATTTTCTGTGCCCCATTCTTCCAAAAATTCCAAAATAGGATTTGCAGCAGA
236663L     TGAGAAGGGGCTAAAACAAACCTCGAAATTTTCTGTGCCCCATTCTTCCAAAAATTCCAAAATAGGATTTGCAGCAGA
H10584L     TGAGAAGGGGCTAAAACAAACCTCGAAATTTTCTGTGCCCCATTCTTCCAAAAATTCCAAAATAGGATTTGCAGCAGA
US9601      TGAGAAGGGGCTAAAACAAACCTCGAAATTTTCTGTGCCCCATTCTTCCAAAAATTCCAAAATAGGATTTGCAGCAGA
H9152       TGAGAAGGGGCTAAAACAAACCTCGAAATTTTCTGTGCCCCATTCTTCCAAAAATTCCAAAATAGGATTTGCAGCAGA
PI564933U   TGAGAAGGGGCTAAAACAAACCTCGAAATTTTCTGTGCCCCATTCTTCCAAAAATTCCAAAATAGGATTTGCAGCAGA
PI229595    -GAGAAGGGGATAAAACAAACCTCAAAATTCTCTGTGCCCCACTCTTCCAGAAATTCCAAAATAGGATTCGCGGCAGA
H3526U1     TGAGAAGGGGATAAAACAAACCTCAAAATTTTCTGTGCCCCACTCTTCCAAAAATTCCAAAATAGGATTTGCGGCAGA
GQ867861    TGAGAAGGGGATAAAACAAACCTCAAAATTTTCTGTGCCCCACTCTTCCAAAAATTCCAAAATAGGATTTGCGGCAGA
GQ867864    TGAGAAGGGGATAAAACAAACCTCAAAATTTTCTGTGCCCCACTCTTCCAAAAATTCCAAAATAGGATTTGCGGCAGA
PI531609L   TGAGAAGGGGATAAAACAAACCTCAAAATTTTCTGTGCCCCACTCTTCCAAAAATTCCAAAATAGGATTTGCGGCAGA
H10391U     TGAGAAGGGGATAAAACAAACCTCAAAATTTTCTGTGCCCCACTCTTCCAAAAATCCCAAAATAGGACTTGCGGCAGA
PI564932L   TGAGAAGGGGATAAAACAAACCTCAAAATTTTCTGTGCCCCACTCTTCCAAAAATTCCAAAATAGGATTTGCGGCAGA
PI345585U   TGAGAAGGGGATAAAACAAACCTCAAAATTTTCTGTGCCCCACTCTTCCAAAAATTCCAAAATAGGATTTGCGGCAGA
PI406467U   TGAGAAGGGGATAAAACAAACCTCAAAATTTTCTGTGCCCCACTCTTCCAAAAATTCCAAAATAGGATTTGCGGCAGA
PI439999U   TGAGAAGGGGATAAAACAAACCTCAAAATTTTCTGTGCCCCACTCTTCCAAAAATTCCAAAATAGGATTTGCGGCAGA
PI564930L   TGAGAAGGGGATAAAACAAACCTCAAAATTTTCTGTGCCCCACTCTTCCAAAAATTCCAAAATAGGATTTGCGGCAGA
PI406448L   TGAGAAGGGGATAAAACAAACCTCAAAATTNTCTGTGCCCCACTCTTCCAAAAATTCCAAAATAGGATTTGCGGCAGA
PI598465L   TGAGAAGGGGATAAAACAAACCTCAAAATTTTCTGTGCCCCACTCTTCCAAAAATTCCAAAATAGGATTTGCGGCAGA
EU187442    TGAGAAGGGGATAAAACAAACCTCAAAATTTTCTGTGCCCCACTCTTCCAAAAATTCCAAAATAGGATTTGCGGCAGA
628702U     TGAGAAGGGGATAAAACAAACCTCAAAATTTTCTGTGCCCCACTCTTCCAAAAATTCCAAAATAGGATTTGCGGCAGA
H10584U     TGAGAAGGGGATAAAACAAACCTCAAAATTTTCTGTGCCCCACTCTTCCGAAAATTCCAAAATAGGATTTGCGGCAGA
531708U     TGAGAAGGGGATAAAACAAACCTCAAAATTTTCTGTGCCCCACTCTCCCAAAAATTCCAAAATAGGATTTGCGGCAGA
499461u1    TGAGAAGGGGATAAAACAAACCTCAAAATTTTCTGTGCCCCACTCTTCCAAAAATTCCAAAATAGGATTTGCCGCAGA
PI228390    TGAGAAGGGGATAAAACAACCCTCAAAATTTTGTGTGCCCCACTCTCCCAAAAATTGGGGGGGGGGACTTGCCCCACA
PI401326    TGAGAAGGGGATAAAACAAACCTCAAAATTTTCTGTGCCCCACTCTTCCAAAAATTCCAAAATAGGATTTGCGGCAGA
PI420842    TGAGAAGGGGATAAAACAAACCTCAAAATTTTCTGGGCCCCACTCTTCCAACAATTCCAAAATTGGATTTGCGGCAGA
PI537379    TGAGAAGGGGATAAAACAAACCTCAAAATTTTCTGTGCCCCACTCTTCCAAAAATTCCAAAATAGGATTTGCGGCAGA
PI516184    TGAGAAGGGGATAAAACAAACCTCAAAATTTTCTGTGCCCCACTCTTCCAAAAATTCCAAAATAGGATTTGCGGCAGA
PI531752    TGAGAAGGGGATAAAACAAACCTCAAAATTTTCTGTGCCCCACTCTTCCAAAAATTCCAAAATAGGATTTGCGGCAGA

E142012     ACCAACAG-TGATATAAACCATCAAGGCAA
E531712     ACCAACAG-TGATATAAACCATCAAGGCAA
W533014     ACCAACAG-TGATATAAACCATCAAGGCAA
W547363     ACCAACAG-TGATATAAACCATCAAGGCAA
P383534     ACCAACAG-TGATATAAACCATCAAGGCAA
PI531609U   ACCAACAG-TGATATAAACCATCAAGGTGA
PI406467L   ACCAACAG-TGATATAAACCATCAAGGCGA
H10339      ACCAACAG-TGATATAAACCATCAAGGCGA
PI439999L   ACCAACAG-TGATATAAACCATCAAGGCGA
PI564932U   ACCAACAG-TGATATAAACCATCAAGGCGA
PI564933L   ACCAACAG-TGATATAAACCATCAAGGCGA
PI564930U   ACCAACAG-TGATATAAACCATCAAGGCGA
PI598465U   ACCAACAG-TGATATAAACCATCAAGGCGA
PI406448U   ACCAACAG-TGATATAAACCATCAAGGCGA
628702L     ACCAACAG-TGATATAAACCATCAAGGCGA
```

H3169L	ACCAACAG-TGATATAAACCATCAAGGCGA
H3526L	ACAAACAG-TGATATAAACCATCAAGGCGA
H10391L	ACCAACAG-TGATATAAACCATCAAGGCGA
499461L	ACCAACAG-TGATATAAACCATCAAGGCGA
H4014	ACCAACAG-TGATATAAACCATCAAGGCGA
H5495one	ACCAACAG-TGATATAAACCATCAAGGCGA
436946	ACCAACAG-TGATATAAACCATCAAGGCGA
232258U	ACCAACAG-TGATATAAACCATCAAGGCGA
531708L	ACCAACAG-TGATATAAACCATCAAGGCGA
H2148	ACCAACAG-TGATATAAACCATCAAGGCGA
H1816	ACCAACAG-TGATATAAACCATCAAGGCGA
H2024	ACCAACAG-TGATATAAACCATCAAGGCGA
H1941	ACCAACAG-TGATATAAACCATCAAGGCGA
331168L	ACAAACAG-TGATATAAACCATCAGGGCGA
236663L	ACCAACAG-AGATATAAACCATCAGGGCGA
H10584L	ACCAACAG-TGATATAAACCATCAGGGCGA
US9601	ACCAACAG-TGATATAAACCATCAGGGCGA
H9152	ACCAACAG-TGATATAAACCATCAGGGCGA
PI564933U	ACCAACAG-TGATATAAACCATCAAGGCGA
PI229595	ACCAACAG-TGATATAAACCATCAAGGCAA
H3526U1	ACCAACAG-TGATATAAACCATCAAGGCAA
GQ867861	ACCAACAG-TGATATAAACCATCAAGGCAA
GQ867864	ACCAACAG-TGATATAAACCATCAAGGCAA
PI531609L	ACCAACAG-TGATATAAACCATCAAGGCAA
H10391U	AACAACAGGTGATATAAACCATCAAGGCAA
PI564932L	ACCAACAG-TGATATAAACCATCAAGGCAA
PI345585U	ACCAACAG-TGATATAAACCATCAAGGCAA
PI406467U	ACCAACAG-TGATATAAACCATCAAGGCAA
PI439999U	ACCAACAG-TGATATAAACCATCAAGGCAA
PI564930L	ACCAACAG-TGATATAAACCATCAAGGCAA
PI406448L	ACCAACAG-TGATATAAACCATCAAGGCAA
PI598465L	ACCAACAG-TGATATAAACCATCAAGGCAA
EU187442	ACCAACAG-TGATATAAACCATCAAGGCAA
628702U	ACCAACAG-TGATATAAACCATCAAGGCAA
H10584U	ACCAACAG-TGATATAAACCATCAAGGCAA
531708U	ACC-ACAG-TGATATAACCCATCAAAGCAA
499461u1	ACCAACAG-TGATATAAACCATCAAGGCAA
PI228390	ACCAACAG-TGATTTAAACCCCCACCCCT-
PI401326	ACCCACAG-TAATATAAACCATCAAGGCAA
PI420842	ACCAACAG-TGATATAAACCATCAACACAA
PI537379	ACCAACAG-TGATATAAACCATCAAGGCAA
PI516184	ACCAACAG-TGATATAAACCATCAAGGCAA
PI531752	ACCAACAG-TGATATAAACCATCAAGGCAA
