# Supplementary material for: Origin and Evolution of Allopolyploid Wheatgrass Elymus fibrosus (Schrenk) Tzvelev (Poaceae: Triticeae) Reveals the Effect of Its Origination on Genetic Diversity
Source: PLoS One. 2016 Dec 9;11(12):e0167795. doi: 10.1371/journal.pone.0167795 (PMC5147983; doi:10.1371/journal.pone.0167795)
Supplement: S2 File — (PDF) [file pone.0167795.s002.pdf]

S2_file. TrnL–trnF sequences used for phylogenetic analysis

```
AF519169    TGGTAACTTCCAAATTCAGAGAAACCCTGGAATTAAAAAAGGGCAATCCT
AF519170    TGGTAACTTCCAAATTCAGAGAAACCCTGGAATTAAAAAAGGGCAATCCT
PI564930    --GTAACTTCCAAATTCAGAGAAACCCTGGAATTAAAAAAGGGCAATCCT
PI345585    --GTAACTTCCAAATTCAGAGAAACCCTGGAATTAAAAAAGGGCAATCCT
PI439999    TGGTAACTTCCAAATTCAGAGAAACCCTGGAATTAAAAAAGGGCAATCCT
PI531609    TGGTAACTTCCAAATTCAGAGAAACCCTGGAATTAAAAAAGGGCAATCCT
PI406467    TGGTAACTTCCAAATTCAGAGAAACCCTGGAATTAAAAAAGGGCAATCCT
PI598465    TGGTAACTTCCAAATTCAGAGAAACCCTGGAATTAAAAAAGGGCAATCCT
PI564932    TGGTAACTTCCAAATTCAGAGAAACCCTGGAATTAAAAAAGGGCAATCCT
KF600685    TGGTAACTTCCAAATTCAGAGAAACCCTGGAATTAAAAAAGGGCAATCCT
PI564933    TGGTAACTTCCAAATTCAGAGAAACCCTGGAATTAAAAAAGGGCAATCCT
AF519145    TGGTAACTTCCAAATTCAGAGAAACCCTGGAATTAAAAAAGGGCAATCCT
EU617289    --------------------------------------------------
EU617255    --------------------------------------------------
EU617284    --------------------------------------------------
EU617269    --------------------------------------------------
EU617283    --------------------------------------------------
KF905221    --------------------GAAACCCTGGAATTAAAAAAGGGCAATCCT
AF519135    TGGTAACTTCCAAATTCAGAGAAACCCTGGAATTAAAAAAGGGCAATCCT
AF519141    TGGTAACTTCCAAATTCAGAGAAACCCTGGAATTAAAAAAGGGCAATCCT
AF519133    TGGTAACTTCCAAATTCAGAGAAACCCTGGAATTAAAAAAGGGCAATCCT
AF519128    TGGTAACTTCCAAATTCAGAGAAACCCTGGAATTAAAAAAGGGCAATCCT
AF519156    TGGTAACTTCCAAATTCAGAGAAACCCTGGAATTAAAAAAGGGCAATCCT
PI406448    TGGTAACTTCCAAATTCAGAGAAACCCTGGAATTAAAAAAGGGCAATCCT
AF519139    TGGTAACTTCCAAATTCAGAGAAACCCTGGAATTAAAAAAGGGCAATCCT
AF519147    TGGTAACTTCCAAATTCAGAGAAACCCTGGAATTAAAAAAGGGCAATCCT
DQ912410    TGGTAACTTCCAAATTCAGAGAAACCCTGGAATTAAAAAAGGGCAATCCT
AF519166    TGGTAACTTCCAAATTCAGAGAAACCCTGGAATTAAAAAAGGGCAATCCT
AF519165    TGGTAACTTCCAAATTCAGAGAAACCCTGGAATTAAAAAAGGGCAATCCT
AB732928    TGGTAACTTCCAAATTCAGAGAAACCCTGGAATTAAAAAAGGGCAATCCT
AF519138    TGGTAACTTCCAAATTCAGAGAAACCCTGGAATTAAAAAAGGGCAATCCT
AF519148    TGGTAACTTCCAAATTCAGAGAAACCCTGGAATTAAAAAAGGGCAATCCT
AF519151    TGGTAACTTCCAAATTCAGAGAAACCCTGGAATTAAAAAAGGGCAATCCT
AF519117    TGGTAACTTCCAAATTCAGAGAAACCCTGGAATTAAAAAAGGGCAATCCT
AF519115    TGGTAACTTCCAAATTCAGAGAAACCCTGGAATTAAAAAAGGGCAATCCT
AF519152    TGGTAACTTCCAAATTCAGAGAAACCCTGGAATTAAAAAAGGGCAATCCT
AF519118    TGGTAACTTCCAAATTCAGAGAAACCCTGGAATTAAAAAAGGGCAATCCT
AF519162    TGGTAACTTCCAAATTCAGAGAAACCCTGGAATTAAAAAAGGGCAATCCT
AF519153    TGATAACTTCCAAATTCAGAGAAACCCTGGAATTAAAAAAGGGCAATCCT
AF519112    TGGTAACTTCCAAATTCAGAGAAACCCTGGAATTAAAAAAGGGCAATCCT
AF519164    TGGTAACTTCCAAATTCAGAGAAACCCTGGAATTAAAAAAGGGCAATCCT
EU013659    TGGTAACTTCCAAATTCAGAGAAACCCTGGAATTAAAAAAGGGCAATCCT
AF519140    TGGTAACTTCCAAATTCAGAGAAACCCTGGAATTAAAAAAGGGCAATCCT
DQ159291    TGGTAACTTCCAAATTCAGAGAAACCCTGGAATTAAAAAAGGGCAATCCT
DQ159290    TGGTAACTTCCAAATTCAGAGAAACCCTGGAATTAAAAAAGGGCAATCCT
AF519158    TGGTAACTTCCAAATTCAGAGAAACCCTGGAATTAAAAAAGGGCAATCCT
AF519160    TGGTAACTTCCAAATTCAGAGAAACCCTGGAATTAAAAAAGGGCAATCCT
AB732935    TGGTAACTTCCAAATTCAGAGAAACCCTGGAATTAAAAAAGGGCAATCCT
AF519125    TGGTAACTTCCAAATTCAGAGAAACCCTGGAATTAAAAAAGGGCAATCCT
AJ969336    ---------------------------------TAAAAAAGGGCAATCCT
AJ969330    TGGTAACTTCCAAATTCAGAGAAACCCTGGAATTAAAAAAGGGCAATCCT
AJ969362    TGGTAACTTCCAAATTCAGAGAAACCCTGGAATTAAAAAAGGGCAATCCT
```

```
FM163499    TGGTAACTTCCAAATTCAGAGAAACCCTGGAATTAAAAAAGGGCAATCCT
AJ969351    TGGTAACTTCCAAATTCAGAGAAACCCTGGAATTAAAAAAGGGCAATCCT
AJ969314    ---------------------------------TAAAAAAGGGCAATCCT
AJ969271    ---------------------------------TAAAAAAGGGCAATCCT
AJ969295    -----------------------ACCCTGGAATTAAAAAAGGGCAATCCT
AJ969296    -----------------------ACCCTGGAATTAAAAAAGGGCAATCCT
AB732937    ------------------------------AATTAAAAAAGGGCAATCCT
AF519124    TGGTAACTTCCAAATTCAGAGAAACCCTGGAATTAAAAAAGGGCAATCCT
KF600706    TGGTAACTTCCAAATTCAGAGAAACCCTGGAATTAAAAAAGGGCAATCCT
AF519122    TGGTAACTTCCAAATTCAGAGAAACCCTGGAATTAAAAAAGGGCAATCCT
AY740789    --------------------------------------------------

AF519169    GAGCCAAATCCGTGTTTTGAGAAAACAAGGGGTTCTCGAACTAGAATACA
AF519170    GAGCCAAATCCGTGTTTTGAGAAAACAAGGGGTTCTCGAACTAGAATACA
PI564930    GAGCCAAATCCGTGTTTTGAGAAAACAAGGGGTTCTCGAACTAGAATACA
PI345585    GAGCCAAATCCGTGTTTTGAGAAAACAAGGGGTTCTCGAACTAGAATACA
PI439999    GAGCCAAATCCGTGTTTTGAGAAAACAAGGGGTTCTCGAACTAGAATACA
PI531609    GAGCCAAATCCGTGTTTTGAGAAAACAAGGGGTTCTCGAACTAGAATACA
PI406467    GAGCCAAATCCGTGTTTTGAGAAAACAAGGGGTTCTCGAACTAGAATACA
PI598465    GAGCCAAATCCGTGTTTTGAGAAAACAAGGGGTTCTCGAACTAGAATACA
PI564932    GAGCCAAATCCGTGTTTTGAGAAAACAAGGGGTTCTCGAACTAGAATACA
KF600685    GAGCCAAATCCGTGTTTTGAGAAAACAAGGGGTTCTCGAACTAGAATACA
PI564933    GAGCCAAATCCGTGTTTTGAGAAAACAAGGGGTTCTCGAACTAGAATACA
AF519145    GAGCCAAATCCGTGTTTTGAGAAAACAAGGGGTTCTCGAACTAGAATACA
EU617289    --------------------------------------------------
EU617255    --------------------------------------------------
EU617284    --------------------------------------------------
EU617269    --------------------------------------------------
EU617283    --------------------------------------------------
KF905221    GAGCCAAATCCGTGTTTTGAGAAAACAAGGGGTTCTCGAACTAGAATACA
AF519135    GAGCCAAATCCGTGTTTTGAGAAAACAAGGGGTTCTCGAACTAGAATACA
AF519141    GAGCCAAATCCGTGTTTTGAGAAAACAAGGGGTTCTCGAACTAGAATACA
AF519133    GAGCCAAATCCGTGTTTTGAGAAAACAAGGGGTTCTCGAACTAGAATACA
AF519128    GAGCCAAATCCGTGTTTTGAGAAAACAAGGGGTTCTCGAACTAGAATACA
AF519156    GAGCCAAATCCGTGTTTTGAGAAAACAAGGGGTTCTCGAACTAGAATACA
PI406448    GAGCCAAATCCGTGTTTTGAGAAAACAAGGGGTTCTCGAACTAGAATACA
AF519139    GAGCCAAATCCGTGTTTTGAGAAAACAAGGGGTTCTCGAACTAGAATACA
AF519147    GAGCCAAATCCGTGTTTTGAGAAAACAAGGGGTTCTCGAACTAGAATACA
DQ912410    GAGCCAAATCCGTGTTTTGAGAAAACAAGGGGTTCTCGAACTAGAATACA
AF519166    GAGCCAAATCCGTGTTTTGAGAAAACAAGGGGTTCTCGAACTAGAATACA
AF519165    GAGCCAAATCCGTGTTTTGAGAAAACAAGGGGTTCTCGAACTAGAATACA
AB732928    GAGCCAAATCCATGTTTTGAGAAAAAAAGGGGTTCTCGAACTAGAATACA
AF519138    GAGCCAAATCCGTGTTTTGAGAAAACAAGGGGTTCTCGAACTAGAATACA
AF519148    GAGCCAAATCCGTGTTTTGAGAAAACAAGGGGTTCTCGAACTAGAATACA
AF519151    GAGCCAAATCCGTGTTTTGAGAAAACAAGGGGTTCTCGAACTAGAATACA
AF519117    GAGCCAAATCCGTGTTTTGAGAAAACAAGGGGTTCTCGAACTAGAATACA
AF519115    GAGCCAAATCCGTGTTTTGAGAAAACAAGGGGTTCTCGAACTAGAATACA
AF519152    GAGCCAAATCCGTGTTTTGAGAAAACAAGGGGTTCTCAAACTAGAATACA
AF519118    GAGCCAAATCCGTGTTTTGAGAAAACAAGGGGTTCTCGAACTAGAATACA
AF519162    GAGCCAAATCCGTGTTTTGAGAAAACAAGGGGTTCTCGAACTAGAATACA
AF519153    GAGCCAAATCCGTGTTTTGAGAAAACAAGGGGTTCTCGAACTAGAATACA
AF519112    GAGCCAAATCCGTGTTTTGAGAAAACAAGGGGTTCTCGAACTAGAATACA
AF519164    GAGCCAAATCCGTGTTTTGAGAAAACAAGGGGTTCTCGAACTAGAATACA
EU013659    GAGCCAAATCCGTGTTTTGAGAAAACAAGGGGTTCTCGAACTAGAATACA
```

AF519140	GAGCCAAATCCGTGTTTTGAGAAAACAAGGGGTTCTCGAACTAGAATACA
DQ159291	GAGCCAAATCCGTGTTTTGAGAAAACAAGGGGTTCTCGAACTAGAATACA
DQ159290	GAGCCAAATCCGTGTTTTGAGAAAACAAGGGGTTCTCGAACTAGAATACA
AF519158	GAGCCAAATCCGTGTTTTGAGAAAACAAGGGGTTCTCGAACTAGAATACA
AF519160	GAGCCAAATCCGTGTTTTGAGAAAACAAGGGGTTCTCGAACTAGAATACA
AB732935	GAGCCAAATCCGTGTTTTGAGAA-----GGGATTCTCGAACTAGAATACA
AF519125	GAGCCAAATCCGTGTTTTGAGAA-----GGGATTCTCGAACTAGAATACA
AJ969336	GAGCCAAATCCGTGTTTTGAGAA-----GGGATTCTCGAACTAGAATACA
AJ969330	GAGCCAAATCCGTGTTTTGAGAA-----GGGATTCTCGAACTAGAATACA
AJ969362	GAGCCAAATCCGTGTTTTGAGAA-----GGGATTCTCGAACTAGAATACA
FM163499	GAGCCAAATCCGTGTTTTGAGAA-----GGGATTCTCGAACTAGAATACA
AJ969351	GAGCCAAATCCGTGTTTTGAGAA-----GGGATTCTCGAACTAGAATACA
AJ969314	GAGCCAAATCCGTGTTTTGAGAA-----GGGATTCTCGAACTAGAATACA
AJ969271	GAGCCAAATCCGTGTTTTGAGAA-----GGGATTCTCGAACTAGAATACA
AJ969295	GAGCCAAATCCGTGTTTTGAGAA-----GGGATTCTCGAACTAGAATACA
AJ969296	GAGCCAAATCCGTGTTTTGAGAA-----GGGATTCTCGAACTAGAATACA
AB732937	GAGCCAAATCCGTGTTTTGAGAA-----GGGATTCTCGAACTAGAATACA
AF519124	GAGCCAAATCCGTGTTTTGAGAA-----GGGATTCTCGAACTAGAATACA
KF600706	GAGCCAAATCCGTGTTTTGAGAA-----GGGATTCTCGAACTAGAATACA
AF519122	GAGCCAAATCCGTGTTTTGAGAA-----GGGATTCTCGAACTAGAATACA
AY740789	--

AF519169	AAGGAAAAGGATAGGTGCAGAGACTCAATGGAAGCTGTTCTAACGAATCG
AF519170	AAGGAAAAGGATAGGTGCAGAGACTCAATGGAAGCTGTTCTAACGAATCG
PI564930	AAGGAAAAGGATAGGTGCAGAGACTCAATGGAAGCTGTTCTAACGAATCG
PI345585	AAGGAAAAGGATAGGTGCAGAGACTCAATGGAAGCTGTTCTAACGAATCG
PI439999	AAGGAAAAGGATAGGTGCAGAGACTCAATGGAAGCTGTTCTAACGAATCG
PI531609	AAGGAAAAGGATAGGTGCAGAGACTCAATGGAAGCTGTTCTAACGAATCG
PI406467	AAGGAAAAGGATAGGTGCAGAGACTCAATGGAAGCTGTTCTAACGAATCG
PI598465	AAGGAAAAGGATAGGTGCAGAGACTCAATGGAAGCTGTTCTAACGAATCG
PI564932	AAGGAAAAGGATAGGTGCAGAGACTCAATGGAAGCTGTTCTAACGAATCG
KF600685	AAGGAAAAGGATAGGTGCAGAGACTCAATGGAAGCTGTTCTAACGAATCG
PI564933	AAGGAAAAGGATAGGTGCAGAGACTCAATGGAAGCTGTTCTAACGAATCG
AF519145	AAGGAAAAGGATAGGTGCAGAGACTCAATGGAAGCTGTTCTAACGAATCG
EU617289	------AAGGATAGGTGCAGAGACTCAATGGAAGCTGTTCTAACGAATCG
EU617255	------AAGGATAGGTGCAGAGACTCAATGGAAGCTGTTCTAACGAATCG
EU617284	------AAGGATAGGTGCAGAGACTCAATGGAAGCTGTTCTAACGAATCG
EU617269	------AAGGATAGGTGCAGAGACTCAATGGAAGCTGTTCTAACGAATCG
EU617283	------AAGGATAGGTGCAGAGACTCAATGGAAGCTGTTCTAACGAATCG
KF905221	AAGGAAAAGGATAGGTGCAGAGACTCAATGGAAGCTGTTCTAACGAATCG
AF519135	AAGGAAAAGGATAGGTGCAGAGACTCAATGGAAGCTGTTCTAACGAATCG
AF519141	AAGGAAAAGGATAGGTGCAGAGACTCAATGGAAGCTGTTCTAACGAATCG
AF519133	AAGGAAAAGGATAGGTGCAGAGACTCAATGGAAGCTGTTCTAACGAATCG
AF519128	AAGGAAAAGGATAGGTGCAGAGACTCAATGGAAGCTGTTCTAACGAATCG
AF519156	AAGGAAAAGGATAGGTGCAGAGACTCAATGGAAGCTGTTCTAACGAATCG
PI406448	AAGGAAAAGGATAGGTGCAGAGACTCAATGGAAGCTGTTCTAACGAATCG
AF519139	AAGGAAAAGGATAGGTGCAGAGACTCAATGGAAGCTGTTCTAACGAATCG
AF519147	AAGGAAAAGGATAGGTGCAGAGACTCAATGGAAGCTGTTCTAACGAATCG
DQ912410	AAGGAAAAGGATAGGTGCAGAGACTCAATGGAAGCTGTTCTAACGAATCG
AF519166	AAGGAAAAGGATAGGTGCAGAGACTCAATGGAAGCTGTTCTAACGAATCG
AF519165	AAGGAAAAGGATAGGTGCAGAGACTCAATGGAAGCTGTTCTAACGAATCG
AB732928	AAGGAAAAGGATAGGTGCAGAGACTCAATGGAAGCTGTTCTAACGAATCG
AF519138	AAGGAAAAGGATAGGTGCAGAGACTCAATGGAAGCTGTTCTAACGAATCG
AF519148	AAGGAAAAGGATAGGTGCAGAGACTCAATGGAAGCTGTTCTAACGAATCG

```
AF519151    AAGGAAAAGGATAGGTGCAGAGACTCAATGGAAGCTGTTCTAACGAATCG
AF519117    AAGGAAAAGGATAGGTGCAGAGACTCAATGGAAGCTGTTCTAACGAATCG
AF519115    AAGGAAAAGGATAGGTGCAGAGACTCAATGGAAGCTGTTCTAACGAATCG
AF519152    AAGGAAAAGGATAGGTGCAGAGACTCAATGGAAGCTGTTCTAACGAATCG
AF519118    AAGGAAAAGGATAGGTGCAGAGACTCAATGGAAGCTGTTCTAACGAATCG
AF519162    AAGGAAAAGGATAGGTGCAGAGACTCAATGGAAGCTGTTCTAACGAATCG
AF519153    AAGGAAAAGGATAGGTGCAGAGACTCAATGGAAGCTGTTCTAACGAATCG
AF519112    AAGGAAAAGGATAGGTGCAGAGACTCAATGGAAGCTGTTCTAACGAATCG
AF519164    AAGGAAAAGGATAGGTGCAGAGACTCAATGGAAGCTGTTCTAACGAATCG
EU013659    AAGGAAAAGGATAGGTGCAGAGACTCAATGGAAGCTGTTCTAACGAATCG
AF519140    AAGGAAAAGGATAGGTGCAGAGACTCAATGGAAGCTGTTCTAACGAATCG
DQ159291    AAGGAAAAGGATAGGTGCAGAGACTCAATGGAAGCTGTTCTAACGAATCG
DQ159290    AAGGAAAAGGATAGGTGCAGAGACTCAATGGAAGCTGTTCTAACGAATCG
AF519158    AAGGAAAAGGATAGGTGCAGAGACTCAATGGAAGCTGTTCTAACGAATCG
AF519160    AAGGAAAAGGATAGGTGCAGAGACTCAATGGAAGCTGTTCTAACGAATCG
AB732935    AAGGAAAAGGATAGGTGCAGAGACTCAATGGAAGCTGTTCTAACGAATCG
AF519125    AAGGAAAAGGATAGGTGCAGAGACTCAATGGAAGCTGTTCTAACGAATCG
AJ969336    AAGGAAAAGGATAGGTGCAGAGACTCAATGGAAGCTGTTCTAACGAATCG
AJ969330    AAGGAAAAGGATAGGTGCAGAGACTCAATGGAAGCTGTTCTAACGAATCG
AJ969362    AAGGAAAAGGATAGGTGCAGAGACTCAATGGAAGCTGTTCTAACGAATCG
FM163499    AAGGAAAAGGATAGGTGCAGAGACTCAATGGAAGCTGTTCTAACGAATCG
AJ969351    AAGGAAAAGGATAGGTGCAGAGACTCAATGGAAGCTGTTCTAACGAATCG
AJ969314    AAGGAAAAGGATAGGTGCAGAGACTCAATGGAAGCTGTTCTAACGAATCG
AJ969271    AAGGAAAAGGATAGGTGCAGAGACTCAATGGAAGCTGTTCTAACGAATCG
AJ969295    AAGGAAAAGGATAGGTGCAGAGACTCAATGGAAGCTGTTCTAACGAATCG
AJ969296    AAGGAAAAGGATAGGTGCAGAGACTCAATGGAAGCTGTTCTAACGAATCG
AB732937    AAGGAAAAGGATAGGTGCAGAGACTCAATGGAAGCTGTTCTAACGAATCG
AF519124    AAGGAAAAGGATAGGTGCAGAGACTCAATGGAAGCTGTTCTAACGAATCG
KF600706    AAGGAAAAGGATAGGTGCAGAGACTCAATGGAAGCTGTTCTAACGAATCG
AF519122    AAGGAAAAGGATAGGTGCAGAGACTCAATGGAAGCTGTTCTAACGAATCG
AY740789    ------AAGGATAGGTGCAGAGACTCAATGGAAGCTGTTCTAACGAATCG

AF519169    AGTTAATTACGTTGTGTTGTTAGTGGAATTCCTTCTAATTCTAAATTAGA
AF519170    AGTTAATTACGTTGTGTTGTTAGTGGAATTCCTTCTAATTCTAAATTAGA
PI564930    AGTTAATTACGTTGTGTTGTTAGTGGAATTCCTTCTAATTCTAAATTAGA
PI345585    AGTTAATTACGTTGTGTTGTTAGTGGAATTCCTTCTAATTCTAAATTAGA
PI439999    AGTTAATTACGTTGTGTTGTTAGTGGAATTCCTTCTAATTCTAAATTAGA
PI531609    AGTTAATTACGTTGTGTTGTTAGTGGAATTCCTTCTAATTCTAAATTAGA
PI406467    AGTTAATTACGTTGTGTTGTTAGTGGAATTCCTTCTAATTCTAAATTAGA
PI598465    AGTTAATTACGTTGTGTTGTTAGTGGAATTCCTTCTAATTCTAAATTAGA
PI564932    AGTTAATTACGTTGTGTTGTTAGTGGAATTCCTTCTAATTCTAAATTAGA
KF600685    AGTTAATTACGTTGTGTTGTTAGTGGAATTCCTTCTAATTCTAAATTAGA
PI564933    AGTTAATTACGTTGTGTTGTTAGTGGAATTCCTTCTAATTCTAAATTAGA
AF519145    AGTTAATTACGTTGTGTTGTTAGTGGAATTCCTTCTAATTCTAAATTAGA
EU617289    AGTTAATTACGTTGTGTTGTTAGTGGAATTCCTTCTAATTCTAAATTAGA
EU617255    AGTTAATTACGTTGTGTTGTTAGTGGAATTCCTTCTAATTCTAAATTAGA
EU617284    AGTTAATTACGTTGTGTTGTTAGTGGAATTCCTTCTAATTCTAAATTAGA
EU617269    AGTTAATTACGTTGTGTTGTTAGTGGAATTCCTTCTAATTCTAAATTAGA
EU617283    AGTTAATTACGTTGTGTTGTTAGTGGAATTCCTTCTAATTCTAAATTAGA
KF905221    AGTTAATTACGTTGTGTTGTTAGTGGAATTCCTTCTAATTCTAAATTAGA
AF519135    AGTTAATTACGTTGTGTTGTTAGTGGAATTCCTTCTAATTCTAAATTAGA
AF519141    AGTTAATTACGTTGTGTTGTTAGTGGAATTCCTTCTAATTCTAAATTAGA
AF519133    AGTTAATTACGTTGTGTTGTTAGTGGAATTCCTTCTAATTCTAAATTAGA
AF519128    AGTTAATTACGTTGTGTTGTTATTGGAATTCCTTCTAATTCTAAATTAGA
```

AF519156	AGTTAATTACGTTGTGTTGTTAGTGGAATTCCTTCTAATTCTAAATTAGA
PI406448	AGTTAATTACGTTGTGTTGTTAGTGGAATTCCTTCTAATTCTAAATTAGA
AF519139	AGTTAATTACGTTGTGTTGTTAGTGGAATTCCTTCTAATTCTAAATTAGA
AF519147	AGTTAATTACGTTGTGTTGTTAGTGGAATTCCTTCTAATTCTAAATTAGA
DQ912410	AGTTAATTACGTTGTGTTGTTAGTGGAATTCCTTCTAATTCTAAATTAGA
AF519166	AGTTAATTACGTTGTGTTGTTAGTGGAATTCCTTCTAATTCTAAATTAGA
AF519165	AGTTAATTACGTTGTGTTGTTAGTGGAATTCCTTCTAATTCTAAATTAGA
AB732928	AGTTAATTACGTTGTGTTGTTAGTGGAATTCCTTCTAATTCTAAATTAGA
AF519138	AGTTAATTACGTTGTGTTGTTAGTGGAATTCCTTCTAATTCTAAATTAGA
AF519148	AGTTAATTACGTTGTGTTGTTAGTGGAATTCCTTCTAATTCTAAATTAGA
AF519151	AGTTAATTACGTTGTGTTGTTAGTGGAATTCCTTCTAATTCTAAATTAGA
AF519117	AGTTAATTACGTTGTGTTGTTAGTGGAATTCCTTCTAATTCTAAATTAGA
AF519115	AGTTAATTACGTTGTGTTGTTAGTGGAATTCCTTCTAATTCTAAATTAGA
AF519152	AGTTAATTACGTTGTGTTGTTAGTGGAATTCCTTCTAATTCTAAATTAGA
AF519118	AGTTAATTACGTTGTGTTGTTAGTGGAATTCCTTCTAATTTTAAATTAGA
AF519162	AGTTAATTACGTTGTGTTGTTAGTGGAATTCCTTCTAATTCTAAATTAGA
AF519153	AGTTAATTACGTTGTGTTGTTAGTGGAATTCCTTCTAATTCTAAATTAGA
AF519112	AGTTAATTACGTTGTGTTGTTAGTGGAATTCCTTCTAATTCTAAATTAGA
AF519164	AGTTAATTACGTTGTGTTGTTAGTGGAATTCCTTCTAATTCTAAATTAGA
EU013659	AGTTAATTACGTTGTGTTGTTAGTGGAATTCCTTCTAATTCTAAATTAGA
AF519140	AGTTAATTACGTTGTGTTGTTAGTGGAATTCCTTCTAATTCTAAATTAGA
DQ159291	AGTTAATTACGTTGTGTTGTTAGTGGAATTCCTTCTAATTCTAAATTAGA
DQ159290	AGTTAATTACGTTGTGTTGTTAGTGGAATTCCTTCTAATTCTAAATTAGA
AF519158	AGTTAATTACGTTGTGTTGTTAGTGGAATTCCTTCTAATTCTAAATTAGA
AF519160	AGTTAATTACGTTGTGTTGTTAGTGGAATTCCTTCTAATTCTAAATTAGA
AB732935	AGTTAATTACGTTGTGTTGTTAGTGGAATTCCTTCTAATTCTAAATTAGA
AF519125	AGTTAATTACGTTGTGTTGTTAGTGGAATTCCTTCTAATTCTAAATTAGA
AJ969336	AGTTAATTACGTTGTGTTGTTAGTGGAATTCCTTCTAATTCTAAATTAGA
AJ969330	AGTTAATTACGTTGTGTTGTTAGTGGAATTCCTTCTAATTCTAAATTAGG
AJ969362	AGTTAATTACGTTGTGTTGTTAGTGGAATTCCTTCTAATTCTAAATTAGA
FM163499	AGTTAATTACGTTGTGTTGTTAGTGGAATTCCTTCTAATTCTAAATTAGA
AJ969351	AGTTAATTACGTTGTGTTGTTAGTGGAATTCCTTCTAATTCTAAATTAGA
AJ969314	AGTTAATTACGTTGTGTTGTTAGTGGAATTCCTTCTAATTCTAAATTAGA
AJ969271	AGTTAATTACGTTGTGTTGTTAGTGGAATTCCTTCTAATTCTAAATTAGA
AJ969295	AGTTAATTACGTTGTGTTGTTAGTGGAATTCCTTCTAATTCTAAATTAGA
AJ969296	AGTTAATTACGTTGTGTTGTTAGTGGAATTCCTTCTAATTCTAAATTAGA
AB732937	AGTTAATTACGTTGTGTTGTTAGTGGAATTCCTTCTAATTCTAAATTAGA
AF519124	AGTTAATTACGTTGTGTTGTTAGTGGAATTCCTTCTAATTCTAAATTAGA
KF600706	AGTTAATTACGTTGTGTTGTTAGTGGAATTCCTTCTAATTCTAAATTAGA
AF519122	AGTTAATTACGTTGTGTTGTTAGTGGAATTCCTTCTAATTCTAAATTAGA
AY740789	AGTTAATTACGTTGTGTTGTTAGTGGAATTCCTTCTAATTCTAAATTAGA

AF519169	GAAAGAGGGGTTTTATACTTTATACATTTAATAAACACGTATAGATACTG
AF519170	GAAAGAGGGGTTTTATACTTTATACATTTAATAAACACGTATAGATACTG
PI564930	GAAAGAGGGGTTTTATACTTTATACATTTAATAAACACGTATAGATACTG
PI345585	GAAAGAGGGGTTTTATACTTTATACATTTAATAAACACGTATAGATACTG
PI439999	GAAAGAGGGGTTTTATACTTTATACATTTAATAAACACGTATAGATACTG
PI531609	GAAAGAGGGGTTTTATACTTTATACATTTAATAAACACGTATAGATACTG
PI406467	GAAAGAGGGGTTTTATACTTTATACATTTAATAAACACGTATAGATACTG
PI598465	GAAAGAGGGGTTTTATACTTTATACATTTAATAAACACGTATAGATACTG
PI564932	GAAAGAGGGGTTTTATACTTTATACATTTAATAAACACGTATAGATACTG
KF600685	GAAAGAGGGGTTTTATACTTTATACATTTAATAAACACGTATAGATACTG
PI564933	GAAAGAGGGGTTTTATACTTTATACATTTAATAAACACGTATAGATACTG
AF519145	GAAAGAGGGGTTTTATACTTTATACATTTAATAAACACGTATAGATACTG

EU617289	GAAAGAGGGGTTTTATACTTTATACATTTAATAAACACGTATAGATACTG
EU617255	GAAAGAGGGGTTTTATACTTTATACATTTAATAAACACGTATAGATACTG
EU617284	GAAAGAGGGGTTTTATACTTTATACATTTAATAAACACGTATAGATACTG
EU617269	GAAAGAGGGGTTTTATACTTTATACATTTAATAAACACGTATAGATACTG
EU617283	GAAAGAGGGGTTTTATACTTTATACATTTAATAAACACGTATAGATACTG
KF905221	GAAAGAGGGGTTTTATACTTTATACATTTAATAAACACGTATAGATACTG
AF519135	GAAAGAGGGGTTTTATACTTTATACATTTAATAAACACGTATAGATACTG
AF519141	GAAAGAGGGGTTTTATACTTTATACATTTAATAAACACGTATAGATACTG
AF519133	GAAAGAGGGGTTTTATACTTTATACATTTAATAAACACGTATAGATACTG
AF519128	GAAAGAGGGGTTTTATACTTTATACATTTAATAAACACGTATAGATACTG
AF519156	GAAAGAGGGGTTTTATACTTTATACATTTAATAAACACGTATAGATACTG
PI406448	GAAAGAGGGGTTTTATACTTTATACATTTAATAAACACGTATAGATACTG
AF519139	GAAAGAGGGGTTTTATACTTTATACATTTAATAAACACGTATAGATACTG
AF519147	GAAAGAGGGGTTTTATACTTTATACATTTAATAAACACGTATAGATACTG
DQ912410	GAAAGAGGGGTTTTATACTTTATACATTTAATAAACACGTATAGATACTG
AF519166	GAAAGAGGGGTTTTATACTTTATACATTTAATAAACACGTATAGATACTG
AF519165	GAAAGAGGGGTTTTATACTTTATACTTTTAATAAACACGTATAGATACTG
AB732928	GAAAGCGGGGTTTTATACTTTATACATTTAATAAACACGTATAGATACTG
AF519138	GAAAGAGGGGTTTTATACTTTATACATTTAATAAACACGTATAGATACTG
AF519148	GAAAGAGGGGTTTTATACTTTATACATTTAATAAACACGTATAGATGCTG
AF519151	GAAAGAGGGGTTTTATACTTTATACATTTAATAAACACGTATAGATGCTG
AF519117	GAAAGAGGGGTTTTATACTTTATACATTTAATAAACACGTATAGATGCTG
AF519115	GAAAGAGGGGTTTTATACTTTATACATTTAATAAACACGTATAGATGCTG
AF519152	GAAAGAGGGGTTTTATACTTTATACATTTAATAAACACGTATAGATACTG
AF519118	GAAAGAGGGGTTTTATACTTTATACATTTAATAAACACGTATAGATACTG
AF519162	GAAAGAGGGGTTTTATACCTTATACATTTAATAAACACGTATAGATACTG
AF519153	GAAAGAGGGGTTTTATACCTTATACATTTAATAAACACGTATAGATACTG
AF519112	GAAAGAGGGGTTTTATACCTTATACATTTAATAAACACGTATAGATACTG
AF519164	GAAAGAGGGGTTTTATACCTTATACATTTAATAAACACGTATAGATACTG
EU013659	GAAAGAGGGGTTTTATACCTTATACATTTAATAAACACGTATAGATACTG
AF519140	GAAAGAGGGGTTTTATACTTTATACATTTAATAAACACGTATAGATACTG
DQ159291	GAAAGAGGGGTTTTATACTTTATACATTTAATAAACACGTATAGATACTG
DQ159290	GAAAGAGGGGTTTTATACTTTATACATTTAATAAACACGTATAGATACTG
AF519158	GAAAGAGGGGTTTTATACTTTATACATTTAATAAACACGTATAGATACTG
AF519160	GAAAGAGGGGTTTTATACTTTATACATTTAATAAACACGTATAGATACTG
AB732935	GAAAGAGGGGTTTTATACTTTATACATTTAATAAACACGTATAGATACTG
AF519125	GAAAGAGGGGTTTTATACTTTATACATTTAATAAACACGTATAGATACTG
AJ969336	GAAAGAGGGGTTTTATACTTTATACATTTAATAAACACGTATAGATACTG
AJ969330	GAAAGAGGGGTTTTATACTTTATACATTTAATAAACACGTATAGATACTG
AJ969362	GAAAGAGGGGTTTTATACTTTATACATTTAATAAACACGTATAGATACTG
FM163499	GAAAGAGGGGTTTTATACTTTATACATTTAATAAACACGTATAGATACTG
AJ969351	GAAAGAGGGGTTTTATACTTTATACATTTAATAAACACGTATAGATACTG
AJ969314	GAAAGAGGGGTTTTATACTTTATACATTTAATAAACACGTATAGATACTG
AJ969271	GAAAGAGGGGTTTTATACTTTATACATTTAATAAACACGTATAGATACTG
AJ969295	GAAAGAGGGGTTTTATACTTTATACATTTAATAAACACGTATAGATACTG
AJ969296	GAAAGAGGGGTTTTATACTTTATACATTTAATAAACACGTATAGATACTG
AB732937	GAAAGAGGGGTTTTATACTTTATACATTTAATAAACACGTATAGATACTG
AF519124	GAAAGAGGGGTTTTATACTTTATACATTTAATAAACACGTATAGATACTG
KF600706	GAAAGAGGGGTTTTATACTTTATACATTTAATAAACACGTATAGATACTG
AF519122	GAAAGAGGGGTTTTATACTTT-------TAATAAACACGTATAGATACTG
AY740789	GAAAGAGGGGTTTTATACTTTATACATTTAATAAACACGTATAGATACTG

AF519169	ACATAGCAAACGATTAATTACAGAACTCATATT-----CTAATAT-AGGT
AF519170	ACATAGCAAACGATTAATTACAGAACTCATATT-----CTAATAT-AGGT

PI564930   ACATAGCAAACGATTAATCACAGAACTCATATTATATTATAATAT-AGGT
PI345585   ACATAGCAAACGATTAATCACAGAACTCATATTATATTATAATAT-AGGT
PI439999   ACATAGCAAACGATTAATCACAGAACTCATATTATATTATAATAT-AGGT
PI531609   ACATAGCAAACGATTAATCACAGAACTCATATTATATTATAATAT-AGGT
PI406467   ACATAGCAAACGATTAATCACAGAACTCATATTATATTATAATAT-AGGT
PI598465   ACATAGCAAACGATTAATCACAGAACTCATATTATATTATAATAT-AGGT
PI564932   ACATAGCAAACGATTAATCACAGAACTCATATTATATTATAATAT-AGGT
KF600685   ACATAGCAAACGATTAATCACAGAACTCATATTATATTATAATAT-AGGT
PI564933   ACATAGCAAACGATTAATCACAGAACTCATATTATATTATAATAT-AGGT
AF519145   ACATAGCAAACGATTAATCACAGAACTCATATTATATTATAATAT-AGGT
EU617289   ACATAGCAAACGATTAATCACAGAACTCATATTATATTATAATATGAGGT
EU617255   ACATAGCAAACGATTAATCACAGAACTCATATTATATTATAATATGAGGT
EU617284   ACATAGCAAACGATTAATCACAGAACTCATATTATATTATAATATGAGGT
EU617269   ACATAGCAAACGATTAATCACAGAACTCATATTATATTATAATATGAGGT
EU617283   ACATAGCAAACGATTAATCACAGAACTCATATT-----ATAATATCAGGT
KF905221   ACATAGCAAACGATTAATCACAGAACTCATATTATATTATAATAT-AGGT
AF519135   ACATAGCAAACGATTAATCACAGAACTCATATTATATTATAATAT-AGGT
AF519141   ACATAGCAAACGATTAATCACAGAACTCATATTATATTATAATAT-AGGT
AF519133   ACATAGCAAACGATTAATCACAGAACTCATATTATATTATAATAT-AGGT
AF519128   ACATAGCAAACGATTAATCACAGAACTCATATTATATTATAATAT-AGGT
AF519156   ACATAGCAAACGATTAATCACAGAACTCATATTATATTATAATAT-AGGT
PI406448   ACATAGCAAACGATTAATCACAGAACTCATATTATAATATAATAT-AGGT
AF519139   ACATAGCAAACGATTAATCACAGAACTCATATTATATTATAATAT-AGGT
AF519147   ACATAGCAAACGATTAATCACAGAACTCATATTATATTATAATAT-AGGT
DQ912410   ACATAGCAAACGATTAATCACAGAACTCATATTATATTATAATAT-AGGT
AF519166   ACATAGCAAACGATTAATCACAGAACTCATATT--ATTATAATAT-AGGT
AF519165   ACATAGCAAACGATTAATCACAGAACTCATATT--ATTATAATAT-AGGT
AB732928   ACATAGCAAACGATTAATCACAGAACTCATAT-----TATAATAT-AGGT
AF519138   ACATAGCAAACGATTAATCACAGAACTCATATTATATTATAATAT-AGGT
AF519148   ACATAGCAAACGATTAATCACAGAACTCATATTATATTATAATAT-AGGT
AF519151   ACATAGCAAACGATTAATCACAGAACTCATATTATATTATAATAT-AGGT
AF519117   ACATAGCAAACGATTAATCACAGAACTCATATTATATTATAATAT-AGGT
AF519115   ACATAGCAAACGATTAATCACAGAACTCATATTATATTATAATAT-AGGT
AF519152   ACATAGCAAACGATTAATCACAGAACTCATATTATAATATAATAT-AGGT
AF519118   ACATAGCAAACGATTAATCACAGAACTCATATTATATTATAATAT-AGGT
AF519162   ACATAGCAAACGATTAATCACAGAACTCATATTA-ATTATAATAT-AGGT
AF519153   ACATAGCAAACGATTAATAACAGAACTCATATT--ATTATAATAT-AGGT
AF519112   ACATAGCAAACGATTAATCACAGAACTCATATTA-ATTATAATAT-AGGT
AF519164   ACATAGCAAACGATTAATCACAGAACTCATATTA-ATTATAATAT-AGGT
EU013659   ACATAGCAAACGATTAATCACAGAACTCATATTA-ATTATAATAT-AGGT
AF519140   ACATAGCAAACGATTAATCACAGAACTCATATTATATTATAATAT-AGGT
DQ159291   ACATAGCAAACGATTAATCACAGAACTCATATT--ATTATAATAT-AGGT
DQ159290   ACATAGCAAACGATTAATCACAGAACTCATATT--ATTATAATAT-AGGT
AF519158   ACATAGCAAACGATTAATCACAGAACTCATATT--ATTATAATAT-AGGT
AF519160   ACATAGCAAACGATTAATCACAGAACTCATATT--ATTATAATAT-AGGT
AB732935   ACATAGCAAACGATTAATCACAGAACTCATATT-----ATAATAT-AGGT
AF519125   ACATAGCAAACGATTAATCACAGAACTCATATT-----ATAATAT-AGGT
AJ969336   ACATAGTAAACGATTAATCACAGAACTCATATT-----ATAATAT-AGGT
AJ969330   ACATAGTAAACGATTAATCACAGAACTCATATT-----ATAATAT-AGGT
AJ969362   ACATAGTAAACGATTAATCACAGAACTCATATT-----ATAATAT-AGGT
FM163499   ACATAGTAAACGATTAATCACAGAACTCATATT-----ATAATAT-AGGT
AJ969351   ACATAGTAAACGATTAATCACAGAACTCATATT-----ATAATAT-AGGT
AJ969314   ACATAGTAAACGATTAATCACAGAACTCATATT-----ATAATAT-AGGT
AJ969271   ACATAGCAAACGATTAATCACAGAACTCATATT-----ATAATAT-AGGT

AJ969295	ACATAGCAAACGATTAATCACAGAACTCATATT-----ATAATAT-AGGT
AJ969296	ACATAGCAAACGATTAATCACAGAACTCATATT-----ATAATAT-AGGT
AB732937	ACATAGTAAACGATTAATCACAGAACTCATATT-----ATAATAT-AGGT
AF519124	ACATAGTAAACGATTAATCACAGAACTCATATT-----ATAATAT-AGGT
KF600706	ACATAGTAAACGATTAATCACAGAACTCATATT-----ATAATAT-AGGT
AF519122	ACATAGCAAACGATTAATCACAGAACTCATATT-----ATAATAT-AGGT
AY740789	ACATAGCAAACGATTAATCACAGAACTCATATT-----ATAATAT-AGGT

AF519169	TCTTTATCTTTTTTAAAA--------ATGAAATT--AGAAT-------GA
AF519170	TCTTTATCTTTTTTAAAA--------ATGAAATT--AGAAT-------GA
PI564930	TCTTTATCTTTTTTT-AAA-------ATGAAA-TTAGAAAT-GATAATGA
PI345585	TCTTTATCTTTTTTT-AAA-------ATGAAA-TTAGAAAT-GATAATGA
PI439999	TCTTTATCTTTTTTT-AAA-------ATGAAA-TTAGAAAT-GATAATGA
PI531609	TCTTTATCTTTTTTT-AAA-------ATGAAA-TTAGAAAT-GATAATGA
PI406467	TCTTTATCTTTTTTT-AAA-------ATGAAA-TTAGAAAT-GATAATGA
PI598465	TCTTTATCTTTTTTT-AAA-------ATGAAA-TTAGAAAT-GATAATGA
PI564932	TCTTTATCTTTTTTT-AAA-------ATGAAA-TTAGAAAT-GATAATGA
KF600685	TCTTTATCTTTTTTT-AAA-------ATGAAA-TTAGAAAT-GATAATGA
PI564933	TCTTTATCTTTTTTT-AAA-------ATGAAA-TTAGAAAT-GATAATGA
AF519145	TCTTTATCTTTTTTT-AAA-------ATGAAA-TTAGAAAT-GATAATGA
EU617289	TCTTTATCTTTTTTT-AAA-------ATGAAATTTAGAAAT-GATAATGA
EU617255	TCTTTATCTTTTTTT-AAA-------ATGAAATTTAGAAAT-GATAATGA
EU617284	TCTTTATCTTTTTTT-AAA-------ATGAAATTTAGAAAT-GATAATGA
EU617269	TCTTTATCTTTTTTT-AAA-------ATGAAATTTAGAAAT-GATAATGA
EU617283	TCTTTATCTTTTTTT-AAA-------ATGAAATTTAGAAAT-GATAATGA
KF905221	TCTTTATCTTTTTTA-AAA-------ATGAAA-TTAGAAAT-GATAATGA
AF519135	TCTTTATCTTTTTTT-AAA-------ATGAAA-TTAGAAAT-GATAATGA
AF519141	TCTTTATCTTTTTTT-AAA-------ATGAAA-TTAGAAAT-GATAATGA
AF519133	TCTTTATCTTTTTTT-AAA-------ATGAAA-TTAGAAAT-GATAATGA
AF519128	TCTTTATCTTTTTTT-AAA-------ATGAAA-TTAGAAA-TGATAATGA
AF519156	TCTTTATCTTTTTTT-AAA-------ATGAAA-TTAGAAA-TGATAATGA
PI406448	TCTTTATCTTTTTT--AAA-------ATGAAA-TTAGAAA-TGATAATGA
AF519139	TCTTTATCTTTTTTT-AAA-------ATGAAA-TTAGAAAT-GATAATGA
AF519147	TCTTTATCTTTTTTTAAAA--------TGAAA-TTAGAAAT-GATAATGA
DQ912410	TCTTTATCTTTTTTTAAAA--------TGAAA-TTAGAAAT-GATAATGA
AF519166	TCTTTATCTTTTTTT-AAA-------ATGAAA-TTAGAAAT-GATAATGA
AF519165	TCTTTATCTTTTTTT-AAA-------ATGAAA-TTAGAAAT-GATAATGA
AB732928	TCTTTATCTTTTTTTAAAA-------ATGAAA-TTAGAAAT-------GA
AF519138	TCTTTATCTTTTTTT-AAA-------ATGAAA-TTAGAAAT-GATAATGA
AF519148	TCTTTATCTTTTTTA-AAA-------ATGAAA-TTAGAAA-------TGA
AF519151	TCTTTATCTTTTTTA-AAA-------ATGAAA-TTAGAAA-------TGA
AF519117	TCTTTATCTTTTTTA-AAA-------ATGAAA-TTAGAAA-------TGA
AF519115	TCTTTATCTTTTTTA-AAA-------ATGAAA-TTAGAAA-------TGA
AF519152	TCTTTATCTTTTTAA-AAA-------ATGAAA-TTAGAAA-------TGA
AF519118	TCTTTATCTTTTTTA-AAA-------ATGAAA-TTAGAAA-------TGA
AF519162	TCTTTATCTTTTTTA-AAA-------ATGAAA-TTCGAAAT-GATAATGA
AF519153	TCTTTATCTTTTTTA-AAA-------ATGAAA-TTCGAAAT-GATAATGA
AF519112	TCTTTATCTTTTTTA-AAA-------ATGAAA-TTAGAAAT-GATAATGA
AF519164	TCTTTATCTTTTTTA-AAA-------ATGAAA-TTAGAAAT-GATAATGA
EU013659	TCTTTATCTTTTTTA-AAA-------ATGAAA-TTAGAAAT-GATAATGA
AF519140	TCTTTATCTTTTTTT-AAA-------ATGAAA-TTAGAAAT-GATAATGA
DQ159291	TCTTTATCTTTTTTT-AAA-------ATGAAA-TTAGAAAT-GATAATGA
DQ159290	TCTTTATCTTTTTTT-AAA-------ATGAAA-TTAGAAAT-GATAATGA
AF519158	TCTTTATCTTTTTTT-AAA-------ATGAAA-TTAGAAAT-GATAATGA

AF519160	TCTTTATCTTTTTTA-AAA-------ATGAAA-TTAGAAAT-GATAATGA
AB732935	TCTTTATCTTTTTTAAAA--------ATAAAATG--AGAAT-------GA
AF519125	TCTTTATCTTTTTTAAAA--------ATAAAATG--AGAAT-------GA
AJ969336	TCTTTATCTTTTTAAAAATTTC-AAAATAAAATT--AGAAT-------GA
AJ969330	TCTTTATCTTTTTTAAAATTTC-AAAATAAAATT--AGAAT-------GA
AJ969362	TCTTTATCTTTTTAAAAATTTC-AAAATAAAATT--AGAAT-------GA
FM163499	TCTTTATCTTTTTAAAAATTTC-AAAATAAAATT--AGAAT-------GA
AJ969351	TCTTTATCTTTTTAAAAATTGC-AAAATAAAATT--AGAAT-------GA
AJ969314	TCTTTATCTTTTTTAAAATTTC-AAAATAAAATT--AGAAT-------GA
AJ969271	TCTTTATCTTTTTTAAAATTTCTAAAATAAAATT--AGAAT-------GA
AJ969295	TCTTTATCTTTTTTAAAATTTC-AAAATAAAATT--CGAAT-------GA
AJ969296	TCTTTATCTTTTTTAAAATTTC-AAAATAAAATT--CGAAT-------GA
AB732937	TCTTTATCTTTTTAAAAATTGC-AAAATAAAATT--AGAAT-------GA
AF519124	TCTTTATCTTTTTTAAAATTTC-AAAATAAAATT--CGAAT-------GA
KF600706	TCTTTATCTTTTTTAAAATTTC-AAAATAAAATT--AGAAT-------GA
AF519122	TCTTTATCTTTTTTTAAATTTC-GAAATAAAATT--AGAAT-------GA
AY740789	TCTTTATCTTTTTTAAAA------AAATAAAATT--AGAAT-------GA

AF519169	TTATGAAATAAAAAACTCATATCATAATTTTTTTTT--GAATTATTGTGA
AF519170	TTATGAAATAAAAAACTCATATCATAATTTTTTTTTT-GAATTATTGTGA
PI564930	TTATGAAATAAAAAACTCATATCATAATTTTTTTTTT--AATTATTGTGA
PI345585	TTATGAAATAAAAAACTCATATCATAATTTTTTTTTT--AATTATTGTGA
PI439999	TTATGAAATAAAAAACTCATATCATAATTTTTTTTTT--AATTATTGTGA
PI531609	TTATGAAATAAAAAACTCATATCATAATTTTTTTTTT--AATTATTGTGA
PI406467	TTATGAAATAAAAAACTCATATCATAATTTTTTTTTT--AATTATTGTGA
PI598465	TTATGAAATAAAAAACTCATATCATAATTTTTTTTTT--AATTATTGTGA
PI564932	TTATGAAATAAAAAACTCATATCATAATTTTTTTTTT--AATTATTGTGA
KF600685	TTATGAAATAAAAAACTCATATCATAATTTTTTTTTT--AATTATTGTGA
PI564933	TTATGAAATAAAAAACTCATATCATAATTTTTTTTTT--AATTATTGTGA
AF519145	TTATGAAATAAAAAACTCATATCATAATTTTTTTTTT--AATTATTGTGA
EU617289	TTATGAAATAAAAAACTCATATCATAATTTTTTTTTTT-AATTATTGTGA
EU617255	TTATGAAATAAAAAACTCATATCATAATTTTTTTTTTT-AATTATTGTGA
EU617284	TTATGAAATAAAAAACTCATATCATAATTTTTTTTTT--AATTATTGTGA
EU617269	TTATGAAATAAAAAACTCATATCATAATTTTTTTTTT--AATTATTGTGA
EU617283	TTATGAAATAAAAAACTCATATCATAATTTTTTTTTT--CATTATTGTGA
KF905221	TTATGAAATGAAAAACTCATATCATAATTTTTTTTTT--AATTATTGTGA
AF519135	TTATGAAATAAAAAACTCATATCATAATTTTTTTTTT--AATTATTGTGA
AF519141	TTATGAAATAAAAAACTCATATCATAATTTTTTTTTT--AATTATTGTGA
AF519133	TTATGAAATAAAAAACTCATATCATAATTTTTTTTTT--AATTATTGTGA
AF519128	TTATGAAATAAAAAACTCATATCATAATTTTTTTTTT--AATTATTGTGA
AF519156	TTATGAAATAAAAAACTCATATCATAATTTTTTTTTT--AATTATTGTGA
PI406448	TTATGAAATAAAAAACTCATATCATAATTTTTTTTTT--AATTATTGTGA
AF519139	TTATGAAATAAAAAACTCATATCATAATTTTTTTTTT--AATTATTGTGA
AF519147	TTATGAAATAAAAAACTCATATCATAATTTTTTTTT---AATTATTGTGA
DQ912410	TTATGAAATAAAAAACTCATATCATAATTTTTTTTTT--AATTATTGTGA
AF519166	TTATGAAATAAAAAACTCATATCATAATTTTTTTTTTT-CATTATTGTGA
AF519165	TTATGAAATAAAAAACTCATATCATAATTTTTTTTTTT-CATTATTGTGA
AB732928	TTATGAA--AAAAAACTCATATCATAATTTTTTTT--------ATTGTGA
AF519138	TTATGAAATAAAAAACTCATATCATAATTTTTTTTTT--CATTATTGTGA
AF519148	TTTTTAAATAAAAAACTCATATCATAATTTTTTTTT----ATTATTGTGA
AF519151	TTTTTAAATAAAAAACTCATATCATAATTTTTTTTT----ATTATTGTGA
AF519117	TTTTTAAATAAAAAACTCATATCATAATTTTTTTTT----ATTATTGTGA
AF519115	TTTTTAAATAAAAAACTCATATCATAATTTTTTTTT----ATTATTGTGA
AF519152	TTTTGAAATAAAAAACTCATATCATAATTTTTTTTTTTTTATTATGGTGA

```
AF519118    TTTTGAAATAAAAAACTCATATCATAATTTTTTTTTT---ATTATTGTGA
AF519162    TTATGAAATAAAAAACTCATATCATAATTTTTTTTTTTTCATTATTGTGA
AF519153    TTATGAAATAAAAAAGTCATATCATAATTTTTTTTTTT-CATTATTGTGA
AF519112    TTATGAAATAAAAAACTCATATCATATAATTTTTTTTTTCATTATTGTGA
AF519164    TTATGAAATAAAAAACTCATATCATAATTTTTTTTTTT-CATTATTGTGA
EU013659    TTATGAAATAAAAAACTCATATCATAATTTTTTTTTT--CATTATTGTGA
AF519140    TTATGAAATAAAAAACTCATATCATAATTTTTTTTT---AATTATTGTGA
DQ159291    TTATGAAATAAAAAACTCATATCATAATTTTTTTTTT--AATTATTGTGA
DQ159290    TTATGAAATAAAAAACTCATATCATAATTTTTTTTTT--AATTATTGTGA
AF519158    TTATGAAATAAAAAACTCATATCATAATTTATTTTTT--AATTATTGTGA
AF519160    TTATGAAATAAAAAACTCATATCATAATTTATTTTTT--AATTATTGTGA
AB732935    TTATGAAATAAAAAACTCATATCATAATTTATTTTT--GAATTATTGTGA
AF519125    TTATGAAATAAAAAACTCATATCATAATTTATTTTT--GAATTATTGTGA
AJ969336    TTATGAAATAAAAAACTCATATCATAATTTATTTTT--GAATTATTGTGA
AJ969330    TTATGAAATAAAAAACTCATATCATAATTTATTTTT--GAATTATTGTGA
AJ969362    TTATGAAATCAAAAACTCATATCATAATTTATTTTT--GAATTATTGTGA
FM163499    TTATGAAATAAAAAACTCATATCATAATTTATTTTT--GAATTATTGTGA
AJ969351    TTATGAAATAAAAAACTCATATCATAATTTATTTTT--GAATTATTGTGA
AJ969314    TTATGAAATAAAAAACTCATATCATAATTTATTTTT--GAATTATTGTGA
AJ969271    TTATGAAATAAAAAACTCATATCATAATTTATTTTT--GAATTATTGTGA
AJ969295    TTATGAAATAAAAAACTCATATCATAATTTATTTTT--GAATTATTGTGA
AJ969296    TTATGAAATAAAAAACTCATATCATAATTTATTTTT--GAATTATTGTGA
AB732937    TTATGAAATAAAAAACTCATATCATAATTTATTTTT--GAATTATTGTGA
AF519124    TTATGAAATAAAAAACTCATATCATAATTTATTTTT--GAATTATTGTGA
KF600706    TTATGAAATAAAAAACTCATATCATAATTTATTTTT--GAATTATTGTGA
AF519122    TTATGAAATAAAAAACTCATATCATAATTTATTTTT--TAATTATTGTGA
AY740789    TTATGAAATAAAAAGCTCATATCATAATTTATTTTT--GAATTATTGTGT

AF519169    ATCCACTCCAATCAAATATTGAATAATCAAATTCTTCAATTCAAATTCAA
AF519170    ATCCACTCCAATCAAATATTGAATAATCAAATTCTTCAATTCAAATTCAA
PI564930    ATCCACTCCAATCGAATATTGAATAATCAAATTCTTCAATTCAAATTCAA
PI345585    ATCCACTCCAATCGAATATTGAATAATCAAATTCTTCAATTCAAATTCAA
PI439999    ATCCACTCCAATCGAATATTGAATAATCAAATTCTTCAATTCAAATTCAA
PI531609    ATCCACTCCAATCGAATATTGAATAATCAAATTCTTCAATTCAAATTCAA
PI406467    ATCCACTCCAATCGAATATTGAATAATCAAATTCTTCAATTCAAATTCAA
PI598465    ATCCACTCCAATCGAATATTGAATAATCAAATTCTTCAATTCAAATTCAA
PI564932    ATCCACTCCAATCGAATATTGAATAATCAAATTCTTCAATTCAAATTCAA
KF600685    ATCCACTCCAATCGAATATTGAATAATCAAATTCTTCAATTCAAATTCAA
PI564933    ATCCACTCCAATCGAATATTGAATAATCAAATTCTTCAATTCAAATTCAA
AF519145    ATCCACTCCAATCGAATATTGAATAATCAAATTCTTCAATTCAAATTCAA
EU617289    ATCCACTCCAATCGAATATTGAATAATCAAATTCTTCAATTCAAATTCAA
EU617255    ATCCACTCCAATCGAATATTGAATAATCAAATTCTTCAATTCAAATTCAA
EU617284    ATCCACTCCAATCGAATATTGAATAATCAAATTCTTCAATTCAAATTCAA
EU617269    ATCCACTCCAATCGAATATTGAATAATCAAATTCTTCAATTCAAATTCAA
EU617283    ATCCACTCCAATCGAATATTGAATAATCAAATTCTTCAATTCAAATTCAA
KF905221    ATCCACTCCAATCGAATATTGAATAATCAAATTCTTCAATTCAAATTCAA
AF519135    ATCCACTCCAATCGAATATTGAATAATCAAATTCTTCAATTCAAATTCAA
AF519141    ATCCACTCCAATCGAATATTGAATAATCAAATTCTTCAATTCAAATTCAA
AF519133    ATCCACTCCAATCGAATATTGAATAATCAAATTCTTCAATTCAAATTCAA
AF519128    ATCCACTCCAATCGAATATTGAATAATCAAATTCTTCAATTCAAATTCAA
AF519156    ATCCACTCCAATCGAATATTGAATAATCAAATTCTTCAATTCAAATTCAA
PI406448    ATCCACTCCAATCGAATATTGAATAATCAAATTCTTCAATTCAAATTCAA
AF519139    ATCCACTCCAATCGAATATTGAATAATCAAATTCTTCAATTCAAATTCAA
AF519147    ATCCACTCCAATCGAATATTGAATAATCAAATTCTTCAATTCAAATTCAA
```

```
DQ912410    ATCCACTCCAATCGAATATTGAATAATCAAATTCTTCAATTCAAATTCAA
AF519166    ATCCACTCCAATCGAATATTGAATAATCAAATTCTTCAATTCAAATTCAA
AF519165    ATCCACTCCAATCGAATATTGAATAATCAAATTCTTCAATTCAAATTCAA
AB732928    ATCCGCTCCAATCGAATATTGAATAATTTGATTATTCAATTCAAATTCAA
AF519138    ATCCACTCCAATCGAATATTGAATAATCAAATTCTTCAATTAAAATTCAA
AF519148    ATCTACTCCAATCGAATATTGAATAATAAAATTCTTCAATTCAAATTCAA
AF519151    ATCTACTCCAATCGAATATTGAATAATAAAATTCTTCAATTCAAATTCAA
AF519117    ATCTACTCCAATCGAATATTGAATAATAAAATTCTTCAATTCAAATTCAA
AF519115    ATCTACTCCAATCGAATATTGAATAATAAAATTCTTCAATTCAAATTCAA
AF519152    ATCTACTCCAATCGAATATTGAATAATAAAATTCTTCAATTCAAATTCAA
AF519118    ATCTACTCCAATCGAATATTGAATAATAAAATTCTGCAATTCAAATTCAA
AF519162    ATCCACTCCAATCGAATATTGAATAATCAAATTCTTCAATTCAAATTCAA
AF519153    ATCCACTCCAATCGAATATTGAATAATGAAATTCTTCAATTCAAATTCAA
AF519112    ATCCACTCCAATCGAATATTGAATAATCAAATTCTTCAATTCAAATTCAA
AF519164    ATCCACTCCAATCGAATATTGAATAATCAAATTCTTCAATTCAAATTCAA
EU013659    ATCCACTCCAATCGAATATTGAATAATCAAATTCTTCAATTCAAATTCAA
AF519140    ATCCACTCCAATCGAATATTGAATAATAAAATTCTTCAATTCAAATTCAA
DQ159291    ATCCACTCCAATCGAATATTGAATAATCAAATTCTTCAATTCAAATTCAA
DQ159290    ATCCACTCCAATCGAATATTGAATAATCAAATTCTTCAATTCAAATTCAA
AF519158    ATCCACTCCAATCGAATATTGAATAATCAAATTCTTCAATTCAAATTCAA
AF519160    ATCCACTCCAATCGAATATTGAATAATCAAATTCTTCAATTCAAATTCAA
AB732935    ATCCACTCCAATCGAATATTGAATAATCAAATTCTTCAATTCAAATTCAA
AF519125    ATCCACTCCAATCGAATATTGAATAATCAAATTCTTCAATTAAAATTCAA
AJ969336    ATCCACTCCATTCGAATATTGAATAATCAAATTCTTCAATTCAAATTCAA
AJ969330    ATCCACTCCATTCGAATATTGAATAATCAAATTCTTCAATTCAAATTCAA
AJ969362    ATCCACTCCATTCGAATATTGAATAATCAAATTCTTCAATTCAAATTCAA
FM163499    ATCCACTCCATTCGAATATTGAATAATCAAATTCTTCAATTCAAATTCAA
AJ969351    ATCCACTCCATTCGAATATTGAATAATCAAATTCTTCAATTCAAATTCAA
AJ969314    ATCCACTCCATTCGAATATTGAATAATCAAATTCTTCAATTCAAATTCAA
AJ969271    ATCCACTCCATTCGAATATTGAATAATCAAATTCTTCAATTCAAATTCAA
AJ969295    ATCCACTCCATTCGAATATTGAATAATCAAATTCTTCAATTCAAATTCAA
AJ969296    ATCCACTCCATTCGAATATTGAATAATCAAATTCTTCAATTCAAATTCAA
AB732937    ATCCACTCCATTCGAATATTGAATAATCAAATTCTTCAATTCAAATTCAA
AF519124    ATCCAC------CGAATATTGAATAATCAAATTCTTCAATTCAAATTCAA
KF600706    ATCCACTCCATTCGAATATTGAATAATCAAATTCTTCAATTCAAATTCAA
AF519122    ATCCACTCCATTCGAATATTGAATAATCAAATTCTTCAATTCAAATTCAA
AY740789    ATCCACTCCATTCGAATATTGAATAATCAAATTCTTCAATTCAAATTCAA

AF519169    AGTTTTCGAGATCTTTAAAAAAGTGGATTAATCGGACGAGGACAAAGAGA
AF519170    AGTTTTCGAGATCTTTAAAAAAGTGGATTAATCGGACGAGGACAAAGAGA
PI564930    AGTTTTCGAGATCTTTAAAAAAGTGGATTAATCGGACGAGGACAAAGAGA
PI345585    AGTTTTCGAGATCTTTAAAAAAGTGGATTAATCGGACGAGGACAAAGAGA
PI439999    AGTTTTCGAGATCTTTAAAAAAGTGGATTAATCGGACGAGGACAAAGAGA
PI531609    AGTTTTCGAGATCTTTAAAAAAGTGGATTAATCGGACGAGGACAAAGAGA
PI406467    AGTTTTCGAGATCTTTAAAAAAGTGGATTAATCGGACGAGGACAAAGAGA
PI598465    AGTTTTCGAGATCTTTAAAAAAGTGGATTAATCGGACGAGGACAAAGAGA
PI564932    AGTTTTCGAGATCTTTAAAAAAGTGGATTAATCGGACGAGGACAAAGAGA
KF600685    AGTTTTCGAGATCTTTAAAAAAGTGGATTAATCGGACGAGGACAAAGAGA
PI564933    AGTTTTCGAGATCTTTAAAAAAGTGGATTAATCGGACGAGGACAAAGAGA
AF519145    AGTTTTCGAGATCTTTAAAAAAGTGGATTAATCGGACGAGGACAAAGAGA
EU617289    AGTTTTCGAGATCTTTAAAAAAGTGGATTAATCGGACGAGGACAAAGAGA
EU617255    AGTTTTCGAGATCTTTAAAAAAGTGGATTAATCGGACGAGGACAAAGAGA
EU617284    AGTTTTCGAGATCTTTAAAAAAGTGGATTAATCGGACGAGGACAAAGAGA
EU617269    AGTTTTCGAGATCTTTAAAAAAGTGGATTAATCGGACGAGGACAAAGAGA
```

```
EU617283    AGTTTTCGAGATCTTTAAAAAAGTGGATTAATCGGACGAGGACAAAGAGA
KF905221    AGTTTTCGAGATCTTTAAAAAAGTGGATTAATCGGACGAGGACAAAGAGA
AF519135    AGTTTTCGAGATCTTTAAAAAAGTGGATTAATCGGACGAGGACAAAGAGA
AF519141    AGTTTTCGAGATCTTTAAAAAAGTGGATTAATCGGACGAGGACAAAGAGA
AF519133    AGTTTTCGAGATCTTTAAAAAAGTGGATTAATCGGACGAGGACAAAGAGA
AF519128    AGTTTTCGAGATCTTTAAAAAAGTGGATTAATCGGACGAGGACAAAGAGA
AF519156    AGTTTTCGAGATCTTTAAAAAAGTGGATTAATCGGACGAGGACAAAGAGA
PI406448    AGTTTTCGAGATCTTTAAAAAAGTGGATTAATCGGACGAGGACAAAGAGA
AF519139    AGTTTTCGAGATCTTTAAAAAAGTGGATTAATCGGACGAGGACAAAGAGA
AF519147    AGTTTTCGAGATCTTTAAAAAAGTGGATTAATCGGACGAGGACAAAGAGA
DQ912410    AGTTTTCGAGATCTTTAAAAAAGTGGATTAATCGGACGAGGACAAAGAGA
AF519166    AGTTTTCGAGATCTTTAAAAAAGTGGATTAATCGGACGAGGACAAAGAGA
AF519165    AGTTTTCGAGATCTTTAAAAAAGTGGATTAATCGGACGAGGACAAAGAGA
AB732928    AGTTTGCGAGATCTTTAAAAAAGTGGATTAATCGGACGAGGACAAAGAGA
AF519138    AGTTTTCGAGATCTTTAAAAAAGTGGATTAATCGGACGAGGACAAAGAGA
AF519148    AGTTTTCGAGATCTTTAAAAAAGTGGATTAATCGGACGAGGACAAAGAGA
AF519151    AGTTTTCGAGATCTTTAAAAAAGTGGATTAATCGGACGAGGACAAAGAGA
AF519117    AGTTTTCGAGATCTTTAAAAAAGTGGATTAATCGGACGAGGACAAAGAGA
AF519115    AGTTTTCGAGATCTTTAAAAAAGTGGATTAATCGGACGAGGACAAAGAGA
AF519152    AGTTTTCGAGATCTTTAAAAAAGTGGATTAATCGAACGAGGACAAAGAGA
AF519118    AGTTTTCGAGATCTTTTAAAAAGTGGATTAATCGGACGAGGACAAAGAGA
AF519162    AGTTTTCGAGATCTTTAAAAAAGCGGATTAATCGGACGAGGACAAAGAGA
AF519153    AGTTTTCGAGATTTTTAAAAAAGTGGATTAATCGGACGAGGACAAAGAGA
AF519112    AGTTTTCGAGATCTTTAAAAAAGTGGATTAATCGGACGAGGACAAAGAGA
AF519164    AGTTTTCGAGATCTTTAAAAAAGTGGATTAATCGGACGAGGACAAAGAGA
EU013659    AGTTTTCGAGATCTTTAAAAAAGTGGATTAATCGGACGAGGACAAAGAGA
AF519140    AGTTTTCGAGATCTTTAAAAAAGTGGATTAATCGGACGAGGACAAAGAGA
DQ159291    AGTTTTCGAGATCTTTAAAAAAGTGGATTAATCGGACGAGGACAAAGAGA
DQ159290    AGTTTTCGAGATCTTTAAAAAAGTGGATTAATCGGACGAGGACAAAGAGA
AF519158    AGTTTTCGAGATCTTTAAAAAAGTGGATTAATCGGACGAGGACAAAGAGA
AF519160    AGTTTTCGAGATCTTTAAAAAAGTGGATTAATCGGACGAGGACAAAGAGA
AB732935    AG-TTTTGAGAACTTTAAAAAAGTGGATTAATCGGACGAGGACAAAGAGA
AF519125    AG-TTTTGAGAACTTTAAAAAAGTGGATTAATCGGACGAGGACAAAGAGA
AJ969336    AG-TTTTGAGAACTTTAAAAAAGTGGATTAATCGGACGAGGACAAAGAGA
AJ969330    AG-TTTTGAGAACTTTAAAAAAGTGGATTAATCGGACGAGGACAAAGAGA
AJ969362    AG-TTTTGAGAACTTTAAAAAAGTGGATTAATCGGACGAGGACAAAGAGA
FM163499    AG-TTTTGAGAACTTTAAAAAAGTGGATTAATCGGACGAGGACAAAGAGA
AJ969351    AG-TTTTGAGAACTTTAAAAAAGTGGATTAATCGGACGAGGACAAAGAGA
AJ969314    AG-TTTTGAGAACTTTGAAAAAGTGGATTAATCGGACGAGGACAAAGAGA
AJ969271    AG-TTTTGAGAACTTTAAAAAAGTGGATTAATCGGACGAGGACAAAGAGA
AJ969295    AG-TTTTGAGAACTTTTAAAAAGAGGATTAATCGGACGAGGACAAAGAGA
AJ969296    AG-TTTTGAGAACTTTTAAAAAGAGGATTAATCGGACGAGGACAAAGAGA
AB732937    AG-TTTTGAGAACTTTAAAAAAGTGGATTAATCGGACGAGGACAAAGAGA
AF519124    AG-TTTTGAGAACTTTCAAAAAGTGGATTAATCGGACGAGGACAAAGAGA
KF600706    AG-TTTTGAGAACTTTAAAAAAGTGGATTAATCGGACGAGGACAAAGAGA
AF519122    AG-TTTTGAGAACTTTTAAAAAGTGGATTAATCGGACGAGGACAAAGAGA
AY740789    AG-TTTTGAGAACTTTAAAAAAGTGGATTAATCGGACGAGGACAAAGAGA

AF519169    GAGTCCCATTCTACATGTCAATACTGACAACAATGAAATTTCTAGTAAAA
AF519170    GAGTCCCATTCTACATGTCAATACTGACAACAATGAAATTTCTAGTAAAA
PI564930    GAGTCCCATTCTACATGTCAATACTGACAACAATGAAATTTCTAGTAAAA
PI345585    GAGTCCCATTCTACATGTCAATACTGACAACAATGAAATTTCTAGTAAAA
PI439999    GAGTCCCATTCTACATGTCAATACTGACAACAATGAAATTTCTAGTAAAA
PI531609    GAGTCCCATTCTACATGTCAATACTGACAACAATGAAATTTCTAGTAAAA
```

```
PI406467    GAGTCCCATTCTACATGTCAATACTGACAACAATGAAATTTCTAGTAAAA
PI598465    GAGTCCCATTCTACATGTCAATACTGACAACAATGAAATTTCTAGTAAAA
PI564932    GAGTCCCATTCTACATGTCAATACTGACAACAATGAAATTTCTAGTAAAA
KF600685    GAGTCCCATTCTACATGTCAATACTGACAACAATGAAATTTCTAGTAAAA
PI564933    GAGTCCCATTCTACATGTCAATACTGACAACAATGAAATTTCTAGTAAAA
AF519145    GAGTCCCATTCTACATGTCAATACTGACAACAATGAAATTTCTAGTAAAA
EU617289    GAGTCCCATTCTACATGTCAATACTGACAACAATGAAATTTCTAGTAAAA
EU617255    GAGTCCCATTCTACATGTCAATACTGACAACAATGAAATTTCTAGTAAAA
EU617284    GAGTCCCATTCTACATGTCAATACTGACAACAATGAAATTTCTAGTAAAA
EU617269    GAGTCCCATTCTACATGTCAATACTGACAACAATGAAATTTCTAGTAAAA
EU617283    GAGTCCCATTCTACATGTCAATACTGACAACAATGCAATTTCTAGTAAAA
KF905221    GAGTCCCATTCTACATGTCAATACTGACAACAATGAAATTTCTAGTAAAA
AF519135    GAGTCCCATTCTACATGTCAATACTGACAACAATGAAATTTCTAGTAAAA
AF519141    GAGTCCCATTCTACATGTCAATACTGACAACAATGAAATTTCTAGTAAAA
AF519133    GAGTCCCATTCTACATGTCAATACTGACAACAATGAAATTTCTAGTAAAA
AF519128    GAGTCCCATTCTACATGTCAATACTGACAACAATGAAATTTCTAGTAAAA
AF519156    GAGTCCCATTCTACATGTCAATACTGACAACAATGAAATTTCTAGTAAAA
PI406448    GAGTCCCATTCTACATGTCAATACTGACAACAATGAAATTTCTAGTAAAA
AF519139    GAGTCCCATTCTACATGTCAATACTGACAACAATGAAATTTCTAGTAAAA
AF519147    GAGTCCCATTCTACATGTCAATACTGACAACAATGAAATTTCTAGTAAAA
DQ912410    GAGTCCCATTCTACATGTCAATACTGACAACAATGAAATTTCTAGTAAAA
AF519166    GAGTCCCATTCTACATGTCAATACTGACAACAATGAAATTTTTAGTAAAA
AF519165    GAGTCCCATTCTACATGTCAATACTGACAACAATGAAATTTCTAGTAAAA
AB732928    GAGTCCCATTCTACATGTCAATACTGACAACAATGAAATTTCTAGTAAAA
AF519138    GAGTCCCATTCTACATGTCAATACTGACAACAATGAAATTTCTAGTAAAA
AF519148    GAGTCCCATTCTACATGTCAATACTGACAACAATGAAATTTCTAGTAAAA
AF519151    GAGTCCCATTCTACATGTCAATACTGACAACAATGAAATTTCTAGTAAAA
AF519117    GAGTCCCATTCTACATGTCAATACTGACAACAATGAAATTTCTAGTAAAA
AF519115    GAGTCCCATTCTACATGTCAATACTGACAACAATGAAATTTCTAGTAAAA
AF519152    GAGTCCCATTCTACATGTCAATACTGACAACAATGAAATTTCTAGTAAAA
AF519118    GAGTCCCATTCTACATGTCAATACTGACAACAATGAAATTTCTAGTAAAA
AF519162    GAGTCCCATTCTACATGTCAATACTGACAACAATGAAATTTCTAGTAAAA
AF519153    GAGTCCCATTCTACATGTCAATACTGACAACAATGAAATTTCTAGTAAAA
AF519112    GAGTCCCATTCTACATGTCAATACTGACAACAATGAAATTTCTAGTAAAA
AF519164    GAGTCCCATTCTACATGTCAATACTGACAACAATGAAATTTCTAGTAAAA
EU013659    GAGTCCCATTCTACATGTCAATACTGACAACAATGAAATTTCTAGTAAAA
AF519140    GAGTCCCATTCTACATGTCAATACTGACAACAATGAAATTTCTAGTAAAA
DQ159291    GAGTCCCATTCTACATGTCAATACTGACAACAATGAAATTTCTAGTAAAA
DQ159290    GAGTCCCATTCTACATGTCAATACTGACAACAATGAAATTTCTAGTAAAA
AF519158    GAGTCCCATTCTACATGTCAATACTGACAACAATGAAATTTCTAGTAAAA
AF519160    GAGTCCCATTCTACATGTCAATACTGACAACAATGAAATTTCTAGTAAAA
AB732935    GAGTCCCATTCTACATGTCAATACTGACAACAATGAAATTTCTAGTAAAA
AF519125    GAGTCCCATTCTACATGTCAATACTGACAACAATGAAATTTCTAGTAAAA
AJ969336    GAGTCCCATTCTACATGTCAATACTGACAACAATGAAATTTCTAGTAAAA
AJ969330    GAGTCCCATTCTACATGTCAATACTGACAACAATGAAATTTCTAGTAAAA
AJ969362    GAGTCCCATTCTACATGTCAATACTGACAACAATGAAATTTCTAGTAAAA
FM163499    GAGTCCCATTCTACATGTCAATACTGACAACAATGAAATTTCTAGTAAAA
AJ969351    GAGTCCCATTCTACATGTCAATACTGACAACAATGAAATTTCTAGTAAAA
AJ969314    GAGTCCCATTCTACATGTCAATACTGACAACAATGAAATTTCTAGTAAAA
AJ969271    GAGTCCCATTCTACATGTCAATACTGACAACAATGAAATTTCTAGTAAAA
AJ969295    GAGTCCCATTCTACATGTCAATACTGACAACAATGAAATTTCTAGTAAAA
AJ969296    GAGTCCCATTCTACATGTCAATACTGACAACAATGAAATTTCTAGTAAAA
AB732937    GAGTCCCATTCTACATGTCAATACTGACAACAATGAAATTTCTAGTAAAA
AF519124    GAGTCCCATTCTACATGTCAATACTGACAACAATGAAATTTCTAGTAAAA
```

```
KF600706    GAGTCCCATTCTACATGTCAATACTGACAACAATGAAATTTCTAGTAAAA
AF519122    GAGTCCCATTCTACATGTCAATACTGACAACAATGAAATTTCTAGTAAAA
AY740789    GAGTCCCATTCCACATGTCAATACTGACAACAATGAAATTTCTAGTAAAA

AF519169    GGAAAATCCGTCGACTTTATAAGTTGTGAGGGTTCAAGTCCCTCTATCCC
AF519170    GGAAAATCCGTCGACTTTATAAGTTGTGAGGGTTCAAGTCCCTCTATCCC
PI564930    GGAAAATCCGTCGACTTTATAAGTTGTGAGGGTTCAAGTCCCTCTATCCC
PI345585    GGAAAATCCGTCGACTTTATAAGTTGTGAGGGTTCAAGTCCCTCTATCCC
PI439999    GGAAAATCCGTCGACTTTATAAGTTGTGAGGGTTCAAGTCCCTCTATCCC
PI531609    GGAAAATCCGTCGACTTTATAAGTTGTGAGGGTTCAAGTCCCTCTATCCC
PI406467    GGAAAATCCGTCGACTTTATAAGTTGTGAGGGTTCAAGTCCCTCTATCCC
PI598465    GGAAAATCCGTCGACTTTATAAGTTGTGAGGGTTCAAGTCCCTCTATCCC
PI564932    GGAAAATCCGTCGACTTTATAAGTTGTGAGGGTTCAAGTCCCTCTATCCC
KF600685    GGAAAATCCGTCGACTTTATAAGTTGTGAGGGTTCAAGTCCCTCTATCCC
PI564933    GGAAAATCCGTCGACTTTATAAGTTGTGAGGGTTCAAGTCCCTCTATCCC
AF519145    GGAAAATCCGTCGACTTTATAAGTTGTGAGGGTTCAAGTCCCTCTATCCC
EU617289    GGAAAATCCGTCGACTTTATAAGTTGTGAGGGTTCAAGTCCCTCTATCCC
EU617255    GGAAAATCCGTCGACTTTATAAGTTGTGAGGGTTCAAGTCCCTCTATCCC
EU617284    GGAAAATCCGTCGACTTTATAAGTTGTGAGGGTTCAAGTCCCTCTATCCC
EU617269    GGAAAATCCGTCGACTTTATAAGTTGTGAGGGTTCAAGTCCCTCTATCCC
EU617283    GGAAAATCCGTCGACTTTATAAGTTGTGAGGGTTCAAGTCCCTCTATCCC
KF905221    GGAAAATCCGTCGACTTTATAAGTTGTGAGGGTTCAAGTCCCTCTATCCC
AF519135    GGAAAATCCGTCGACTTTATAAGTTGTGAGGGTTCAAGTCCCTCTATCCC
AF519141    GGAAAATCCGTCGACTTTATAAGTTGTGAGGGTTCAAGTCCCTCTATCCC
AF519133    GGAAAATCCGTCGACTTTATAAGTTGTGAGGGTTCAAGTCCCTCTATCCC
AF519128    GGAAAATCCGTCGACTTTATAAGTTGTGAGGGTTCAAGTCCCTCTATCCC
AF519156    GGAAAATCCGTCGACTTTATAAGTTGTGAGGGTTCAAGTCCCTCTATCCC
PI406448    GGAAAATCCGTCGACTTTATAAGTTGTGAGGGTTCAAGTCCCTCTATCCC
AF519139    GGAAAATCCGTCGACTTTATAAGTTGTGAGGGTTCAAGTCCCTCTATCCC
AF519147    GGAAAATCCGTCGACTTTATAAGTTGTGAGGGTTCAAGTCCCTCTATCCC
DQ912410    GGAAAATCCGTCGACTTTATAAGTTGTGAGGGTTCAAGTCCCTCTATCCC
AF519166    GGAAAATCCGTCGACTTTATAAGTTGTGAGGGTTCAAGTCCCTCTATCCC
AF519165    GGAAAATCCGTCGACTTTATAAGTTGTGAGGGTTCAAGTCCCTCTATCCC
AB732928    GGAAAATCCGTCGACTTTATAAGTTGTGAGGGTTCAAGTCCCTCTATCCC
AF519138    GGAAAATCCGTCGACTTTATAAGTTGTGAGGGTTCAAGTCCCTCTATCCC
AF519148    GGAAAATCCGTCGACTTTATAAGTTGTGAGGGTTCAAGTCCCTCTATCCC
AF519151    GGAAAATCCGTCGACTTTATAAGTTGTGAGGGTTCAAGTCCCTCTATCCC
AF519117    GGAAAATCCGTCGACTTTATAAGTTGTGAGGGTTCAAGTCCCTCTATCCC
AF519115    GGAAAATCCGTCGACTTTATAAGTTGTGAGGGTTCAAGTCCCTCTATCCC
AF519152    GGAAAATCCGTCGACTTTATAAGTTGTGAGGGTTCAAGTCCCTCTATCCC
AF519118    GGAAAATCCGTCGACTTTATAAGTTGTGAGGGTTCAAGTCCCTCTATCCC
AF519162    GGAAAATCCGTCGACTTTATAAGTTGTGAGGGTTCAAGTCCCTCTATCCC
AF519153    GGAAAATCCGTCGACTTTATAAGTTGTGAGGGTTCAAGTCCCTCTATCCC
AF519112    GGAAAATCCGTCGACTTTATAAGTTGTGAGGGTTCAAGTCCCTCTATCCC
AF519164    GGAAAATCCGTCGACTTTATAAGTTGTGAGGGTTCAAGTCCCTCTATCCC
EU013659    GGAAAATCCGTCGACTTTATAAGTTGTGAGGGTTCAAGTCCCTCTATCCC
AF519140    GGAAAATCCGTCGACTTTATAAGTTGTGAGGGTTCAAGTCCCTCTATCCC
DQ159291    GGAAAATCCGTCGACTTTATAAGTTGTGAGGGGTCAAGTCCCTCTATCCC
DQ159290    GGAAAATCCGTCGACTTTATAAGTTGTGAGGGTTCAAGTCCCTNNATCCC
AF519158    GGAAAATCCGTCGACTTTATAAGTTGTGAGGGTTCAAGTCCCTCTATCCC
AF519160    GGAAAATCCGTCGACTTTATAAGTTGTGAGGGTTCAAGTCCCTCTATCCC
AB732935    GGAAAATCCGTCGACTTTATAAGTTGTGAGGGTTCAAGTCCCTCTATCCC
AF519125    GGAAAATCCGTCGACTTTATAAGTTGTGAGGGTTCAAGTCCCTCTATCCC
AJ969336    GGAAAATCCGTCGACTTTATAAGTTGTGAGGGTTCAAGTCCCTCTATCCC
```

AJ969330	GGAAAATCCGTCGACTTTATAAGTTGTGAGGGTTCAAGTCCCTCTATCCC
AJ969362	GGAAAATCCGTCGACTTTATAAGTTGTGAGGGTTCAAGTCCCTCTATCCC
FM163499	GGAAAATCCGTCGACTTTATAAGTTGTGAGGGTTCAAGTCCCTCTATCCC
AJ969351	GGAAAATCCGTCGACTTTATAAGTTGTGAGGGTTCAAGTCCCTCTATCCC
AJ969314	GGAAAATCCGTCGACTTTATAAGTTGTGAGGGTTCAAGTCCCTCTATCCC
AJ969271	GGAAAATCCGTCGACTTTATAAGTTGTGAGGGTTCAAGTCCCTCTATCCC
AJ969295	GGAAAATCCGTCGACTTTTTAAGTTGTGAGGGTTCAAGTCCCTCTATCCC
AJ969296	GGAAAATCCGTCGACTTTTTAAGTTGTGAGGGTTCAAGTCCCTCTATCCC
AB732937	GGAAAATCCGTCGACTTTATAAGTTGTGAGGGTTCAAGTCCCTCTATCCC
AF519124	GGAAAATCCGTCGACTTTATAAGTTGTGAGGGTTCAAGTCCCTCTATCCC
KF600706	GGAAAATCCGTCGACTTTATAAGTTGTGAGGGTTCAAGTCCCTCTATCCC
AF519122	GGAAAATCCGTCGACTTTATAAGTTGTGAGGGTTCAAGTCCCTCTATCCC
AY740789	GGAAAATCCGTCGACTTTATAAGTTGTGAGGGTTCAAGTCCCTCTATCCC

AF519169	CAAATCCTCTTTTATCCCCTAACTATA-CTATAGTATTTATCCT----CT
AF519170	CAAATCCTCTTTTATCCCCTAACTATA-CTATAGTATTTATCCT----CT
PI564930	CAAATCCTCTTTTATTCCCTAACTATA-CTATATTATTTATCCTCTTTTT
PI345585	CAAATCCTCTTTTATTCCCTAACTATA-CTATATTATTTATCCTCTTTTT
PI439999	CAAATCCTCTTTTATTCCCTAACTATA-CTATATTATTTATCCTCTTTTT
PI531609	CAAATCCTCTTTTATTCCCTAACTATA-CTATATTATTTATCCTCTTTTT
PI406467	CAAATCCTCTTTTATTCCCTAACTATA-CTATATTATTTATCCTCTTTTT
PI598465	CAAATCCTCTTTTATTCCCTAACTATA-CTATATTATTTATCCTCTTTTT
PI564932	CAAATCCTCTTTTATTCCCTAACTATA-CTATATTATTTATCCTCTTTTT
KF600685	CAAATCCTCTTTTATTCCCTAACTATA-CTATATTATTTATCCTCTTTTT
PI564933	CAAATCCTCTTTTATTCCCTAACTATA-CTATATTATTTATCCTCTTTTT
AF519145	CAAATCCTCTTTTATTCCCTAACTATA-CTATATTATTTATCCTCTTTTT
EU617289	CAAATCCTCTTTTATTCCCTAACTATAGCTATATTATTTATCCTCTTTTT
EU617255	CAAATCCTCTTTTATTCCCTAACTATAGCTATATTATTTATCCTCTTTTT
EU617284	CAAATCCTCTTTTATTCCCTAACTATAGCTATATTATTTATCCTCTTTTT
EU617269	CAAATCCTCTTTTATTCCCTAACTATAGCTATATTATTTATCCTCTTTTT
EU617283	CAAATCCTCTTTTATTCCCTAACTATAGCTATATTATTTATCCTCTTTTT
KF905221	CAAATCCTCTTTTATTCCCTAACTATA-CTATATTATTTATCCTCTTTTT
AF519135	CAAATCCTCTTTTATTCCCTAACTATA-CTATATTATTTATCCTCTTTTT
AF519141	CAAATCCTCTTTTATTCCCTAACTATA-CTATATTATTTATCCTCTTTTT
AF519133	CAAATCCTCTTTTATTCCCTAACTATA-CTATATTATTTATCCTCTTTTT
AF519128	CAAATCCTCTTTTATTCCCTAACTATA-CTATATTATTTATCCTCTTTTT
AF519156	CAAATCCTCTTTTATTCCCTAACTATA-CTATATTATTTATCCTCTTTTT
PI406448	CAAATCCTCTTTTATTCCCTAACTATA-CTATATTATTTATCCTCTTTTT
AF519139	CAAATCCTCTTTTATTCCCTAACTATA-CTATATTATTTATCCTCTTTTT
AF519147	CAAATCCTCTTTTATTCCCTAACTATA-CTATATTATTTATCCTCTTTTT
DQ912410	CAAATCCTCTTTTATTCCCTAACTATA-CTATATTATTTATCCTCTTTTT
AF519166	CAAATCCTCTTTTATTCCCTAACTATA-CTATATTATTTATCCTCTTTTT
AF519165	CAAATCCTCTTTTATTCCCTAACTATA-CTATATTATTTATCCTCTTTTT
AB732928	CAAATCCTCTTTTATTCCCTAACTATA-TTAT--TAATCCTC-TTTTTTT
AF519138	CAAATCCTCTTTTATTCCCTAACTATA-CTATATTATTTATCCTCTTTTT
AF519148	CAAATCCTCTTTTATTCCCTAACTATA------TTATTTATCCTCTTTTT
AF519151	CAAATCCTCTTTTATTCCCTAACTATA------TTATTTATCCTCTTTTT
AF519117	CAAATCCTCTTTTATTCCCTAACTATA------TTATTTATCCTCTTTTT
AF519115	CAAATCCTCTTTTATTCCCTAACTATA------TTATTTATCCTCTTTTT
AF519152	CAAATCCTCTTTTATTCCCTAACTATA------TTATTTATCCTCTTTTT
AF519118	CAAATCCTCTTTTATTCCTTAACTATA------TTATTTATCCTCTTTTT
AF519162	CAAATCCTCTTTTATTCCCTAACTATAACTATACTATTTATCCTCTTTT-
AF519153	CAAATCCTATTTTATTCCCTAACTATAACTATATTTTTATCCTCTTTTT
AF519112	CAAATCCTCTTTTATTCCCTAACTATA-CTATATTATTTATCCTCTTTTT

```
AF519164    CAAATCCTCTTTTATTCCCTAACTATA-CTATATTATTTATCCTC-TTTT
EU013659    CAAATCCTCTTTTATTCCCTAACTATA-CTATATTATTTATCCTCTTTTT
AF519140    CAAATCCTCTTTTATTCCCTAACTATA-CTATATTATTTATCCTCTTTTT
DQ159291    CAAATCCTNTTTTATTCCCTAACTATA-CTATATTATTTATCCTCTTTTT
DQ159290    CAAATCCTTTTTTATTCCCTAACTATA-CTATATTATTTATCCTCTTTTT
AF519158    CAAATCCTCTTTTATTCCCTAACTATA-CTATATTATTTATCCTCTTTTT
AF519160    CAAATCCTCTTTTATTCCCTAACTATA-CTATATTATTTATCCTCTTTTT
AB732935    CAAATCCTCTTTT-----------------------------------TT
AF519125    CAAATCCTCTTTT-----------------------------------TT
AJ969336    CAAATCCTCTTTT-----------------------------------NN
AJ969330    CAAATCCTCTTTT-----------------------------------NN
AJ969362    CAAATCCTCTTTT-----------------------------------TN
FM163499    CAAATCCTCTTTT-----------------------------------TT
AJ969351    CAAATCCTCTTTT-----------------------------------TT
AJ969314    CAAATCCTCTTTT-----------------------------------TT
AJ969271    CAAATCCTCTTTT-----------------------------------TT
AJ969295    CAAATCCTCTTTT-----------------------------------TT
AJ969296    CAAATCCTCTTTT-----------------------------------TT
AB732937    CAAATCCTCTTTT-----------------------------------TT
AF519124    CAAATCCTCTTTT-----------------------------------TT
KF600706    CAAATCCTCTTTT-----------------------------------TT
AF519122    CAAATCCTCTTTT-----------------------------------TT
AY740789    CAAATCCTCTTTT-----------------------------------TT

AF519169    TTTTTTTTTATCAATGGGTTTAAGATTCATTAGCTTTCTCATTCTACTCT
AF519170    TTTTTTTT-ATCCATGGGTTTAAGATTCATTAGCTTTCTCATTCTACTCT
PI564930    TTTCTTTTTATCAATGGGTTTAAGATTCATTAGCTTTCTCATTCTACTCT
PI345585    TTTCTTTTTATCAATGGGTTTAAGATTCATTAGCTTTCTCATTCTACTCT
PI439999    TTTCTTTTTATCAATGGGTTTAAGATTCATTAGCTTTCTCATTCTACTCT
PI531609    TTTCTTTTTATCAATGGGTTTAAGATTCATTAGCTTTCTCATTCTACTCT
PI406467    TTTCTTTTTATCAATGGGTTTAAGATTCATTAGCTTTCTCATTCTACTCT
PI598465    TTTCTTTTTATCAATGGGTTTAAGATTCATTAGCTTTCTCATTCTACTCT
PI564932    TTTCTTTTTATCAATGGGTTTAAGATTCATTAGCTTTCTCATTCTACTCT
KF600685    TTTCTTTTTATCAATGGGTTTAAGATTCATTAGCTTTCTCATTCTACTCT
PI564933    TTTCTTTTTATCAATGGGTTTAAGATTCATTAGCTTTCTCATTCTACTCT
AF519145    TTTCTTTTTATCAATGGGTTTAAGATTCATTAGCTTTCTCATTCTACTCT
EU617289    TTTCTTTTTATCAATGGGTTTAAGATTCATTAGCTTTCTCATTCTACTCT
EU617255    TTTCTTTTTATCAATGGGTTTAAGATTCATTAGCTTTCTCATTCTACTCT
EU617284    TTTCTTTTTATCAATGGGTTTAAGATTCATTAGCTTTCTCATTCTACTCT
EU617269    TTTCCTTTTATCAATGGGTTTAAGATTCATTAGCTTTCTCATTCTACTCT
EU617283    TTTCTTTTTATCAATGGGTTTAAGATTCATTAGCTTTCTCATTCTACTCT
KF905221    TTTCTTTTTATCAATGGGTTCAAGATTCATTAGCTTTCTCATTCTACTCT
AF519135    TTTCTTTTTATCAATGGGTTTAAGATTCATTAGCTTTCTCATTCTACTCT
AF519141    TTTCTTTTTATCAATGGGTTTAAGATTCATTAGCTTTCTCATTCTACTCT
AF519133    TTTCTTTTTATCAATGGGTTTAAGATTCATTAGCTTTCTCATTCTACTCT
AF519128    TTT----CTTTTAATGGGTTTAAGATTCATTAGCTTTCTCATTCTACTCT
AF519156    TTTCTTTTTATCAATGGGTTTAAGATTCATTAGCTTTCTCATTCTACTCT
PI406448    TTTCTTTTTATCAATGGGTTTAAGATTCATTAGCTTTCTCATTCTACTCT
AF519139    TTTCTTTTTATCAATGGGTTTAAGATTCATTAGCTTTCTCATTCTACTCT
AF519147    TTTCTTTTTATCAATGGGTTTAAGATTCATTAGCTTTCTCATTCTACTCT
DQ912410    TTTCTTTTTATCAATGGGTTTAAGATTCATTAGCTTTCTCATTCTACTCT
AF519166    TTTCTTTTTATCAATGGGTTTAAGATTCATTAGCTTTCTCATTCTACTCT
AF519165    TTTCTTTTTATCAATGGGTTTAAGATTCATTAGCTTTCTCATTCTACTCT
AB732928    TTTCTTTTTATCAATGGGTTTAAGATTCATTAGCTTTCTCATTCTACTCT
```

AF519138	TTTCTTTTTATCAATGGGTTTAAGATTCATTAGCTTTCTCATTCTACTCT
AF519148	TTTCTTTTTATCAATGGGTTTAAGATTCATTAGCTTTCTCATTCTACTCT
AF519151	TTTCTTTTTATCAATGGGTTTAAGATTCATTAGCTTTCTCATTCTACTCT
AF519117	TTTCTTTTTATCAATGGGTTTAAGATTCATTAGCTTTCTCATTCTACTCT
AF519115	TTTCTTTTTATCAATGGGTTTAAGATTCATTAGCTTTCTCATTCTACTCT
AF519152	TTTCTTTTTATCAATGGGTTTAAGATTCATTAGCTTTCTCATTCTACTCT
AF519118	TTTCTTTTTATCAATGGGTTTAAGATTCATTAGCTTTCTCATTCTACTAT
AF519162	----TTTTTATCAATGGGTTTAAGATTCATTAGCTTTCTCATTCTACTCT
AF519153	TTTCTTTTTATCAATGGGTTTAAGATTCATTAGCTTTCTCATTCTACTCT
AF519112	TTTCTTTTTATCAATGGGTTTAAGATTCATTAGCTTTCTCATTCTACTCT
AF519164	TTTCTTTTTATCAATGGGTTTAAGATTCATTAGCTTTCTCATTCTACTCT
EU013659	TTTCTTTTTATCAATGGGTTTAAGATTCATTAGCTTTCTCATTCTACTCT
AF519140	TTTCTTTTTATCAATGGGTTTAAGATTCATTAGCTTTCTCATTCTACTCT
DQ159291	TTTCTTTTTATCAATGGGTTTAAGATTCATTAGCTTTCTCATTCTACTCT
DQ159290	TTTCTTTTTATCAATGGGTTTAAGATTCATTAGCTTTCTCATTCTACTCT
AF519158	TTTCTTTTTATCAATGGGTTTAAGATTCATTAGCTTTCTCATTCTACTCT
AF519160	TTTCTTTTTATCAATGGGTTTAAGATTCATTAGCTTTCTCATTCTACTCT
AB732935	TT-CTTTTTATCAATGGGTTTAAGATTCATTAGCTTTCTCATTCTACTCT
AF519125	TTTCTTTTTATCAATGGGTTTAAGATTCATTAGCTTTCTCATTCTACTCT
AJ969336	NNNNNTTTTATCAATGGGTTTAAGATTCATTAGCTTTCTCATTCTACTCT
AJ969330	NNNNTTTTTATCAATGGGTTTAAGATTCATTAGCTTTCTCATTCTACTCT
AJ969362	NNNNTTTTTATCAATGGGTTTAAGATTCATTAGCTTTCTCATTCTACTCT
FM163499	NNNNNTTTTATCAATGGGTTTAAGATTCATTAGCTTTCTCATTCTACTCT
AJ969351	NNNNTTTTTATCAATGGGTTTAAGATTCATTAGCTTTCTCATTCTACTCT
AJ969314	NNNNNNNTTATCAATGGGTTTAAGATTCATTAGCTTTCTCATTCTACTCT
AJ969271	NNNNNTTTTATCAATGGGTTTAAGATTCATTAGCTTTCTCATTCTACTCT
AJ969295	NNNNNTTTTATCAATGGGTTTAAGATTCATTAGCTTTCTCATTCTACTCT
AJ969296	NNNNNTTTTATCAATGGGTTTAAGATTCATTAGCTTTCTCATTCTACTCT
AB732937	TT-CTTTTTATCAATGGGTTTAAGATTCATTAGCTTTCTCATTCTACTCT
AF519124	TTTCTTTTTATCAATGGGTTTAAGATTCATTAGCTTTCTCATTCTACTCT
KF600706	TTTCTTTTTATCAATGGGTTTAAGATTCATTAGCTTTCTCATTCTACTCT
AF519122	TTTCTTTTTATCAATGGGTTTAAGATTCATTAGCTTTCTCATTCTACTCT
AY740789	TT-CTTTTTATCAATGGGTTTAAGATTCATTAGCTTTCTCATTCTACTCT

AF519169	TTCACAAAGGAATGCGAAGAGAACTCAATGGATCTTATCCTATTCATTGA
AF519170	TTCACAAAGGAATGCGAAGAGAACTCAATGGATCTTATCCTATTCATTGA
PI564930	TTCACAAAGGAATGCGAAGAGAACTCAATGGATCTTATCCTATTCATTGA
PI345585	TTCACAAAGGAATGCGAAGAGAACTCAATGGATCTTATCCTATTCATTGA
PI439999	TTCACAAAGGAATGCGAAGAGAACTCAATGGATCTTATCCTATTCATTGA
PI531609	TTCACAAAGGAATGCGAAGAGAACTCAATGGATCTTATCCTATTCATTGA
PI406467	TTCACAAAGGAATGCGAAGAGAACTCAATGGATCTTATCCTATTCATTGA
PI598465	TTCACAAAGGAATGCGAAGAGAACTCAATGGATCTTATCCTATTCATTGA
PI564932	TTCACAAAGGAATGCGAAGAGAACTCAATGGATCTTATCCTATTCATTGA
KF600685	TTCACAAAGGAATGCGAAGAGAACTCAATGGATCTTATCCTATTCATTGA
PI564933	TTCACAAAGGAATGCGAAGAGAACTCAATGGATCTTATCCTATTCATTGA
AF519145	TTCACAAAGGAATGCGAAGAGAACTCAATGGATCTTATCCTATTCATTGA
EU617289	TTCACAAAGGAATGCGAAGAGAACTCAATGGATCTTATCCTATTCATTGA
EU617255	TTCACAAAGAAATGCGAAGAGAACTCAATGGATCTTATCCTATTCATTGA
EU617284	TTCACAAAGGAATGCGAAGAGAACTCAATGGATCTTATCCTATTCATTGA
EU617269	TTCACAAAGGAATGCGAAGAGAACTCAATGGATCTTATCCTATTCATTGA
EU617283	TTCACAAAGGAATGCGAAGAGAACTCAATGGATCTTATCCTATTCATTGA
KF905221	TTCACAAAGGAATGCGAAGAGAACTCAATGGATCTTATCCTATTCATTGA
AF519135	TTCACAAAGGAATGCGAAGAGAACTCAATGGATCTTATCCTATTCATTGA
AF519141	TTCACAAAGGAATGCGAAGAGAACTCAATGGATCTTATCCTATTCATTGA

```
AF519133    TTCACAAAGGAATGCGAAGAGAACTCAATGGATCTTATCCTATTCATTGA
AF519128    TTCACAAAGGAATGCGAAGAGAACTCAATGGATCTTATCCTATTCATTGA
AF519156    TTCACAAAGGAATGCGAAGAGAACTCAATGGATCTTATCCTATTCATTGA
PI406448    TTCACAAAGGAATGCGAAGAGAACTCAATGGATCTTATCCTATTCATTGA
AF519139    TTCACAAAGGAATGCGAAGAGAACTCAATGGATCTTATCCTATTCATTGA
AF519147    TTCACAAAGGAATGCGAAGAGAACTCAATGGATCTTATCCTATTCATTGA
DQ912410    TTCACAAAGGAATGCGAAGAGAACTCAATGGATCTTATCCTATTCATTGA
AF519166    TTCACAAAGGAATGCGAAGAGAACTCAATGGATCTTATCCTATTCATTGA
AF519165    TTCACAAAGGAATGCGAAGAGAACTCAATGGATCTTATCCTATTCATTGA
AB732928    TTCACAAAGGAATGCGAAGAGAACTCAATGGATCTTATCCTATTCATTGA
AF519138    TTCACAAAGGAATGCGAAGAGAACTCAATGGATCTTATCCTATTCATTGA
AF519148    TTCACAAAGGAATGCGAAGAGAACTCAATGGATCTTATCCTATTCATTGA
AF519151    TTCACAAAGGAATGCGAAGAGAACTCAATGGATCTTATCCTATTCATTGA
AF519117    TTCACAAAGGAATGCGAAGAGAACTCAATGGATCTTATCCTATTCATTGA
AF519115    TTCACAAAGGAATGCGAAGAGAACTCAATGGATCTTATCCTATTCATTGA
AF519152    TTCACAAAGGAATGCGAAGAGAACTCAATGGATCTTATCCTATTCATTGA
AF519118    TTCACAAAGGAATGCGAAGAGAACTCAATGGATCTTATCCTATTCATTGA
AF519162    TTCACAAAGGAATGCGAAGAGAACTCAATGGATCTTATCCTATTCATTGA
AF519153    TTCACAAAGGAATGCGAAGAGAACTCAATGGATCTTATCCTATTCATTGA
AF519112    TTCACAAAGGAATGCGAAGAGAACTCAATGGATCTTATCCTATTCATTGA
AF519164    TTCACAAAGGAATGCGAAGAGAACTCAATGGATCTTATCCTATTCATTGA
EU013659    TTCACAAAGGAATGCGAAGAGAACTCAATGGATCTTATCCTATTCATTGA
AF519140    TTCACAAAGGAATGCGAAGAGAACTCAATGGATCTTATCCTATTCATTGA
DQ159291    TTCACAAAGGAATGCGAAGAGAACTCAATGGATCTTATCCTATTCATTGA
DQ159290    TTCACAAAGGAATGCGAAGAGAACTCAATGGATCTTATCCTATTCATTGA
AF519158    TTCACAAAGGAATGCGAAGAGAACTCAATGGATCTTATCCTATTCATTGA
AF519160    TTCACAAAGGAATGCGAAGAGAACTCAATGGATCTTATCCTATTCATTGA
AB732935    TTCACAAAGGAATGCGAAGAGAACTCAATGGATCTTATCCTATTCATTGA
AF519125    TTCACAAAGGAATGCGAAGAGAACTCAATGGATCTTATCCTATTCATTGA
AJ969336    TTCACAAAGGAATGCGAAGAGAACTCAATGGATCTTATCCTATTCATTGA
AJ969330    TTCACAAAGGAATGCGAAGAGAACTCAATGGATCTTATCCTATTCATTGA
AJ969362    TTCACAAAGGAATGCGAAGAGAACTCAATGGATCTTATCCTATTCATTGA
FM163499    TTCACAAAGGAATGCGAAGAGAACTCAATGGATCTTATCCTATTCATTGA
AJ969351    TTCACAAAGGAATGCGAAGAGAACTCAATGGATCTTATCCTATTCATTGA
AJ969314    TTCACAAAGGAATGCGAAGAGAACTCAATGGATCTTATCCTATTCATTGA
AJ969271    TTCACAAAGGAATGCGAAGAGAACTCAATGGATCTTATCCTATTCATTGA
AJ969295    TTCACAAAGGAATGCGAAGAGAACTCAATGGATCTTATCCTATTCATTGA
AJ969296    TTCACAAAGGAATGCGAAGAGAACTCAATGGATCTTATCCTATTCATTGA
AB732937    TTCACAAAGGAATGCGAAGAGAACTCAATGGATCTTATCCTATTCATTGA
AF519124    TTCACAAAGGAATGCGAAGAGAACTCAATGGATCTTATCCTATTCATTGA
KF600706    TTCACAAAGGAATGCGAAGAGAACTCAATGGATCTTATCCTATTCATTGA
AF519122    TTCACAAAGGAATGCGAAGAAAACTCAATGGATCTTATCCTATTCATTGA
AY740789    TTCACAAAGGAATGCGAAGAGAACTCAATGGATCTTATCCTATTCATTGA

AF519169    ATAGATTACTTTTTT-ATTAGAGTATCGGCAAGAAATCTTGGTTATT-CA
AF519170    ATAGATTACTTTTTT-ATTAGAGTATCGGCAAGAAATCTTGGTTATT-CA
PI564930    ATAGATTTCTTTTTT-ATTAGAGTATCGGCAAGAAATCTTGGTTATT--C
PI345585    ATAGATTTCTTTTTT-ATTAGAGTATCGGCAAGAAATCTTGGTTATTCAC
PI439999    ATAGATTTCTTTTTTTATTAGAGTATCGGCAAGAAATCTTGGTTATT--C
PI531609    ATAGATTTCTTTTTT-ATTAGAGTATCGGCAAGAAATCTTGGTTATT--A
PI406467    ATAGATTTCTTTTTT-ATTAGAGTATCGGCAAGAAATCTTGGTTTTT--A
PI598465    ATAGATTTCTTTTTT-ATTAGAGTATCGGCAAGAAATCTTGGTTATT--A
PI564932    ATAGATTTCTTTTTT-ATTAGAGTATCGGCAAGAAATCTTGGTTATT--A
KF600685    ATAGATTTCTTTTTT-ATTAGAGTATCGGCAAGAAATCTTGGTTATT--A
```

PI564933	ATAGATTTCTTTTTT-ATTAGAGTATCGGCAAGAAATCTTGGTTATT--A
AF519145	ATAGATTTCTTTTTT-ATTAGAGTATCGGCAAGAAATCTTGGTTATT-CA
EU617289	ATAGATTTCTTTTTT-ATTAGAGTATCGGCAAGAAATCTTGGTTATT-CA
EU617255	ATAGATTTCTTTTTT-ATTAGAGTATCGGCAAGAAATCTTGGTTATT-CA
EU617284	ATAGATTTCTTTTTT-ATTAGAGTATCGGCAAGAAATCTTGGTTATT-CA
EU617269	ATAGATTTCTTTTTT-ATTAGAGTATCGGCAAGAAATCTTGGTTATT-CA
EU617283	ATAGATTTCTTTTTT-ATTAGAGTATCGGCAAGAAATCTTGGTTATT-CA
KF905221	ATAGATTTCTTTTTT-ATTAGAGTATCGGCAAGAAATCTTGGTTATT-CA
AF519135	ATAGATTTCTTTTTT-ATTAGAGTATCGGCAAGAAATCTTGGTTATT-CA
AF519141	ATAGATTTCTTTTTT-ATTAGAGTATCGGCAAGAAATCTTGGTTATT-CA
AF519133	ATAGATTTCTTTTTT-ATTAGAGTATCGGCAAGAAATCTTGGTTATT-CA
AF519128	ATAGATTTCTTTTTT-ATTAGAGTATCGGCAAGAAATCTTGGTTATT-CA
AF519156	ATAGATTTCTTTTTT-ATTAGAGTATCGGCAAGAAATCTTGGTTATT-CA
PI406448	ATAGATTTCTTTTTT-ATTAGAGTATCGGCAAGAAATCTTGGTTATT-CA
AF519139	ATAGATTTCTTTTTT-ATTAGAGTATCGGCAAGAAATCTTGGTTATT-CA
AF519147	ATAGATTTCTTTTTT-ATTAGAGTATCGGCAAGAAATCTTGGTTATT-CA
DQ912410	ATAGATTTCTTTTTT-ATTAGAGTATCGGCAAGAAATCTTGGTTATT-CA
AF519166	ATAGATTTCTTTTTT-ATTAGAGTATCGGCAAGAAATCTTGGTTATT-CA
AF519165	ATAGATTTCTTTTTT-ATTAGAGTATCGGCAAGAAATCTTGGTTATT-CA
AB732928	ATAGATTTCTTTTTT-ATTAGAGTATCGGCAAGAAATCTTGGTTATTTCA
AF519138	ATAGATTTCTTTTTT-ATTAGAGTATCGGCAAGAAATCTTGGTTATT-CA
AF519148	ATAGATTTCTTTTTT-ATTAGAGTATCGGCAAGTAATCTTGGTTTTT-CA
AF519151	ATAGATTTCTTTTTT-ATTAGAGTATCGGCAAGTAATCTTGGTTATT-CA
AF519117	ATAGATTTCTTTTTT-ATTAGAGTATCGGCAAGTAATCTTGGTTATT-CA
AF519115	ATAGATTTCTTTTTT-ATTAGAGTATCGGCAAGTAATCTTGGTTATT-CA
AF519152	ATAGATTTCTTTTTT-ATTAGAGTATCGGCAAGAAATCTTGGTTATT-CA
AF519118	ATAGATTTCTTTTTT-ATTAGAGTACCGGCAAGAAATCTTGGTTATT-CA
AF519162	ATAGATTTCTTTTTT-ATTAGAGTATCGGCAAGAAATCTTGGTTATT-CA
AF519153	ATAGATTTCTTTTTT-ATTAGAGTATCGGCAAGAAATCTTGGTTATT-CA
AF519112	ATAGATTTCTTTTTT-ATTAGAGTATCGGCAAGAAATCTTGGTTATT-CA
AF519164	ATAGATTTCTTTTTT-ATTAGAGTATCGGCAAGAAATCTTGGTTATT-CA
EU013659	ATAGATTTCTTTTTT-ATTAGAGTATCGGCGAGAAATCTTGGTTATT-CA
AF519140	ATAGATTTCTTTTTT-ATTAGAGTATCGGCAAGAAATCTTGGTTATT-CA
DQ159291	ATAGATTTCTTTTTT-ATTAGAGTATCGGCAAGAAATCTTGGTTATT-CA
DQ159290	ATAGATTTCTTTTTT-ATTAGAGTATCGGCAAGAAATCTTGGTTATT-CA
AF519158	ATAGATTTCTTTTTT-ATTAGAGTATCGGCAAGAAATCTTGGTTATT-CA
AF519160	ATAGATTTCTTTTTT-ATTAGAGTATCGGCAAGAAATCTTGGTTATT-CA
AB732935	ATAGATTTCTTTTTT-ATTAGAGTATCGGCAAGAAATCTTGGTTATT-CA
AF519125	ATAGATTTCTTTTTT-ATTAGAGTATCGGCAAGAAATCTTGGTTATT-CG
AJ969336	ATAGATTTCTTTTTT-ATTAGAGTATCGGCAAGAAATCTTGGTTATT-CA
AJ969330	ATAGATTTCTTTTTT-ATTAGAGTATCGGCAAGAAATCTTGGTTATT-CA
AJ969362	ATAGATTTCTTTTTT-ATTAGAGTATCGGCAAGAAATCTTGGTTATT-CA
FM163499	ATAGATTTCATTTTT-ATTAGAGTATCGGCAAGAAATCTTGGTTATT-CA
AJ969351	ATAGATTTCTTTTTT-ATTAGAGTATCGGCAAGAAATCTTGGTTATT-CA
AJ969314	ATAGATTTCTTTTTT-ATTAGAGTATCGGCAAGAAATCTTGGTTATT-CA
AJ969271	ATAGATTTCTTTTTT-ATTAGAGTATCGGCAAGAAATCTTGGTTATT-CA
AJ969295	ATAGATTTCTTTTTT-ATTAGAGTATCGGCAAGAAATCTTGGTTATT-CA
AJ969296	ATAGATTTCTTTTTT-ATTAGAGTATCGGCAAGAAATCTTGGTTATT-CA
AB732937	ATAGATTTCTTTTTT-ATTAGAGTATCGGCAAGAAATCTTGGTTATT-CA
AF519124	ATAGATTTCTTTTTT-ATTAGAGTATCGGCAAGAAATCTTGGTTATT-CA
KF600706	ATAGATTTCTTTTTT-ATTAGAGTATTGGCAAGAAATCTTGGTTATT-CA
AF519122	ATAGATTTCTTTTTT-ATTAGAGTATCGGCAAGAAATCTTGGTTATT-CA
AY740789	ATAGATTTCTTTTTT-ATTAGAGTATCGGCAAGAAATCTTGGTTATT-CA

```
AF519169    CTCTAT-TTTTAAGTATTATTTAAGTAAACCATGCACA
AF519170    CTCTAT-TTTTAAGTATTATTTAAGTAAACCATGCACA
PI564930    CTCTAT-TTTTAAGTATTATTTAAGTAAACCATGCACA
PI345585    CTCTAT-TTTTAAGTATTATTTAAGTAAACCATGCACA
PI439999    CTCTATATTTTNAGTATTATTTAAGTAAACCATGCACA
PI531609    CTCTAT-TTTTAAGTATTATTTAAGTAAACCATGCACA
PI406467    CTCTAT-TTTTAAGTATTATTTAAGTAAACCATGCACA
PI598465    CTCTAT-TTTTAAGTATTATTTAAGTAAACCATGCACA
PI564932    CTCTAT-TTTTAAGTATTATTTAAGTAAACCATGCACA
KF600685    CTCTAT-TTTTAAGTATTATTTAAGTAAACCATGCACA
PI564933    CTCTAT-TTTTAAGTATTATTTAAGTAAACCATGCACA
AF519145    CTCTAT-TTTTAAGTATTATTTAAGTAAACCATGCACA
EU617289    CTCTAT-TTTTAAGTATTATTTAAGTAAACCATGCACA
EU617255    CTCTAT-TTTTAAGTATTATTTAAGTAAACCATGCACA
EU617284    CTCTAT-TTTTAAGTATTATTTAAGTAAACCATGCACA
EU617269    CTCTAT-TTTTAAGTATTATTTAAGTAAACCATGCACA
EU617283    CTCTAT-TTTTAAGTATTATTTAAGTAAATCATGCACA
KF905221    CTCTAT-TTTTAAGTATTATTTAAGTAAACCATGCACA
AF519135    CTCTAT-TTTTAAGTATTATTTAAGTAAACCATGCACA
AF519141    CTCTAT-TTTTAAGTATTATTTAAGTAAACCATGCACA
AF519133    CTCTAT-TTTTAAGTATTATTTAAGTAAACCATGCACA
AF519128    CTCTAT-TTTTAAGTATTATTTAAGTAAACCATGCACA
AF519156    CTCTAT-TTTTAAGTATTATTTAAGTAAACCATGCACA
PI406448    CTCTAT-TTTTAAGTATTATTTAAGTAAACCATGCACA
AF519139    CTCTAT-TTTTAAGTATTATTTAAGTAAACCATGCACA
AF519147    CTCTAT-TTTTAAGTATTATTTAAGTAAACCATGCACA
DQ912410    CTCTAT-TTTTAAGTATTATTTAAGTAAACCATGCACA
AF519166    CTCTAT-TTTTAAGTATTATTTAAGTAAACCATGCACA
AF519165    CTCTAT-TTTTAAGTATTATTTAAGTAAACCATGCACA
AB732928    CTCTAT-TTTTAAGTATTGTTTAAGTAAACCATGCACA
AF519138    CTCTAT-TTTTAAGTATTATTTAAGTAAACCATGCACA
AF519148    CTCTAT-TTTAAAGTATTATTTAAGTAAACCATGCACA
AF519151    CTCTAT-TTTAAAGTATTATTTAAGTAAACCATGCACA
AF519117    CTCTAT-TTTCAAGTATTATTTAAGTAAACCATGCACA
AF519115    CTCTAT-TTTCAAGTATTATTTAAGTAAACCATGCACA
AF519152    CTCTAT-TTTTAAGTATTATTTAAGTAAACCATGCACA
AF519118    CTCTAT-TTTTAAGTATTATTTAAGTAAACCATGCACA
AF519162    CTCCAT-TTTTAAGTTTTATTTAAGTAAACCATGCACA
AF519153    CTCTAT-TTTTAAGTTTTATTTAAGTAAACCATGCACA
AF519112    CTCTAT-TTTTAAGTTTTATTTAAGTAAACCATGCACA
AF519164    CTCTAT-TTTTAAGTTTTATTTAAGTAAACCATGCACA
EU013659    CTCTAT-TTTTAAGTTTTATTTAAGTAAACCATGCACA
AF519140    CTCTAT-TTTTAAGTATTATTTAAGTAAACCATGCACA
DQ159291    CTCTAT-TTTTAAGTATTATTTAAGTAAACCATGCACA
DQ159290    CTCTAT-TTTTAAGTATTATTTAAGTAAACCATGCACA
AF519158    CTCTAT-TTTTAAGTATTATTTAAGTAAACCATGCACA
AF519160    CTCTAT-TTTTAAGTATTATTTAAGTAAACCATGCACA
AB732935    CTCTAT-TTTTAAGTATTATTTAAGTAAACCATGCACA
AF519125    CTCTAT-TTTTAAGTATTATTTAAGTAAACCATGCACA
AJ969336    CTCTAT-TTTTAAGTATTATTTAAGTAAACCATGCACA
AJ969330    CTCTAT-TTTTAAGTATTATTTAAGTAAACCATGCACA
AJ969362    CTCTAT-TTTTAAGTATTATTTAAGTAAACCATGCACA
FM163499    CTCTAT-TTTTAAGTATTATTTAAGTAAACCATGCACA
AJ969351    CTCTAT-TTTTAAGTATTATTTAAGTAAACCATGCACA
```

```
AJ969314    CTCTAT-TTTTAAGTATTATTTAAGTAAACCATGCACA
AJ969271    CTCTAT-TTTTAAGTATTATTTAAGTAAACCATGCACA
AJ969295    CTCTAT-TTTTAAGTATTATTTAAGTAAACCATGCACA
AJ969296    CTCTAT-TTTTAAGTATTATTTAAGTAAACCATGCACA
AB732937    CTCTAT-TTTTAAGTATTATTTAAGTAAACCATGCACA
AF519124    CTCTAT-TTTTAAGTATTATTTAAGTAAACCATGCACA
KF600706    CTCTAT-TTTTAAGTATTATTTAAGTAAACCATGCACA
AF519122    CTCTAT-TTTTAAGTATTATTTAAGTAAACCATGCACA
AY740789    CTCTAT-TTTTAAGTATTATTTAAGTAAACCATGCACA
;
end;
```
